# Long-term risks and benefits associated with cesarean delivery for mother, baby, and subsequent pregnancies: Systematic review and meta-analysis

**DOI:** 10.1371/journal.pmed.1002494

**Published:** 2018-01-23

**Authors:** Oonagh E. Keag, Jane E. Norman, Sarah J. Stock

**Affiliations:** 1 NHS Lothian Department of Obstetrics and Gynaecology, Simpson’s Centre for Reproductive Health, Royal Infirmary of Edinburgh, Edinburgh, United Kingdom; 2 Tommy’s Centre for Maternal and Fetal Health, MRC Centre for Reproductive Health, University of Edinburgh Queen’s Medical Research Institute, Edinburgh, United Kingdom; 3 School of Women’s and Infants’ Health, University of Western Australia, Crawley, Australia; University of Manchester, UNITED KINGDOM

## Abstract

**Background:**

Cesarean birth rates continue to rise worldwide with recent (2016) reported rates of 24.5% in Western Europe, 32% in North America, and 41% in South America. The objective of this systematic review is to describe the long-term risks and benefits of cesarean delivery for mother, baby, and subsequent pregnancies. The primary maternal outcome was pelvic floor dysfunction, the primary baby outcome was asthma, and the primary subsequent pregnancy outcome was perinatal death.

**Methods and findings:**

Medline, Embase, Cochrane, and Cumulative Index to Nursing and Allied Health Literature (CINAHL) databases were systematically searched for published studies in human subjects (last search 25 May 2017), supplemented by manual searches. Included studies were randomized controlled trials (RCTs) and large (more than 1,000 participants) prospective cohort studies with greater than or equal to one-year follow-up comparing outcomes of women delivering by cesarean delivery and by vaginal delivery. Two assessors screened 30,327 abstracts. Studies were graded for risk of bias by two assessors using the Scottish Intercollegiate Guideline Network (SIGN) Methodology Checklist and the Risk of Bias Assessment tool for Non-Randomized Studies. Results were pooled in fixed effects meta-analyses or in random effects models when significant heterogeneity was present (I^2^ ≥ 40%).

One RCT and 79 cohort studies (all from high income countries) were included, involving 29,928,274 participants. Compared to vaginal delivery, cesarean delivery was associated with decreased risk of urinary incontinence, odds ratio (OR) 0.56 (95% CI 0.47 to 0.66; *n* = 58,900; 8 studies) and pelvic organ prolapse (OR 0.29, 0.17 to 0.51; *n* = 39,208; 2 studies). Children delivered by cesarean delivery had increased risk of asthma up to the age of 12 years (OR 1.21, 1.11 to 1.32; *n* = 887,960; 13 studies) and obesity up to the age of 5 years (OR 1.59, 1.33 to 1.90; *n* = 64,113; 6 studies). Pregnancy after cesarean delivery was associated with increased risk of miscarriage (OR 1.17, 1.03 to 1.32; *n* = 151,412; 4 studies) and stillbirth (OR 1.27, 1.15 to 1.40; *n* = 703,562; 8 studies), but not perinatal mortality (OR 1.11, 0.89 to 1.39; *n* = 91,429; 2 studies). Pregnancy following cesarean delivery was associated with increased risk of placenta previa (OR 1.74, 1.62 to 1.87; *n* = 7,101,692; 10 studies), placenta accreta (OR 2.95, 1.32 to 6.60; *n* = 705,108; 3 studies), and placental abruption (OR 1.38, 1.27 to 1.49; *n* = 5,667,160; 6 studies).

This is a comprehensive review adhering to a registered protocol, and guidelines for the Meta-analysis of Observational Studies in Epidemiology were followed, but it is based on predominantly observational data, and in some meta-analyses, between-study heterogeneity is high; therefore, causation cannot be inferred and the results should be interpreted with caution.

**Conclusions:**

When compared with vaginal delivery, cesarean delivery is associated with a reduced rate of urinary incontinence and pelvic organ prolapse, but this should be weighed against the association with increased risks for fertility, future pregnancy, and long-term childhood outcomes. This information could be valuable in counselling women on mode of delivery.

## Abstract 2

In a systematic review and meta-analysis, Sarah Stock exmines the long-term risks and benefits associated with cesarean delivery for mother, baby, and subsequent pregnancies.

## Author summary

**Why was this study done?:**

- Cesarean delivery rates are rising worldwide, and in particular, rates of cesarean delivery without medical indication are increasing.
- The short-term associations of cesarean delivery are well described in the literature, but women are less informed of the long-term risks and benefits of cesarean delivery on themselves, their offspring, and their future pregnancies.
- This review aims to synthesize the available evidence on the long-term associations with cesarean delivery.

**What did the researchers do and find?:**

- We performed a systematic review looking for randomized controlled trials and large prospective cohort studies that assessed long-term outcomes following caesarean delivery compared with vaginal delivery.
- We found that cesarean delivery is associated with reduced urinary incontinence and pelvic organ prolapse in the mother but with increased odds of asthma and obesity in the child.
- Cesarean delivery is associated with future subfertility and several subsequent pregnancy risks such as placenta previa, uterine rupture, and stillbirth.

**What do these findings mean?:**

- These findings might help enhance discussions between clinicians and patients regarding mode of delivery, meaning that patients will be better informed of the potential long-term risks and benefits of cesarean delivery for themselves, their offspring, and any future pregnancies.
- The majority of included data are from observational studies that were performed in high-income countries. This means that the results should be interpreted with caution, and findings may not be generalizable to other healthcare settings.

## Introduction

Rates of cesarean delivery continue to rise worldwide, with recent (2016) reported rates of 24.5% in Western Europe, 32% in North America, and 41% in South America [1,2]. In the presence of maternal or fetal complications, cesarean delivery can effectively reduce maternal and perinatal mortality and morbidity [2]; however, an increasing proportion of babies are delivered by cesarean when there is no medical or obstetric indication [3]. The short-term adverse associations of cesarean delivery for the mother, such as infection, haemorrhage, visceral injury, and venous thromboembolism, have been minimized to the point that cesarean delivery is considered as safe as vaginal delivery in high-income countries [4], though in low- and middle-income countries, there is an increased risk of adverse short-term maternal outcomes even with cesarean delivery without medical indication [1]. This notwithstanding, the long-term risks and benefits of cesarean delivery for mother, baby, and subsequent pregnancies are less frequently discussed with women, and there are few randomized controlled trials (RCTs) addressing the issue [5,6]. Systematic reviews of observational studies investigating the longer-term associations of cesarean delivery provide conflicting results on risks and benefits for mother and baby [7–13].

Maternal preferences are an important influence on decisions about mode of delivery. At present, evidence of longer-term complications of cesarean delivery has not been adequately synthesized to allow fully informed decisions about mode of delivery to be made. The aim of this systematic review and meta-analysis is to summarize the evidence about long-term risks and benefits of cesarean delivery for women, children, and the associations with future pregnancies.

## Methods

We conducted a systematic review of literature according to the recommendations of the Meta-analysis Of Observational Studies in Epidemiology (MOOSE) Guidelines for Meta-Analyses and Systematic Reviews of Observational Studies [14]. The study protocol was registered with the University of York Centre for Reviews and Dissemination International prospective register of systematic reviews (PROSPERO Record CRD42014007006, http://www.crd.york.ac.uk/PROSPERO/).

We developed and tested the search strategy in collaboration with a librarian experienced in literature searching. We searched Cumulative Index to Nursing and Allied Health Literature (CINAHL) and Cochrane library databases. The search terms are described in S1 Table; searches began 23 March 2014, and the last search was 25 May 2017. Additional studies were identified from reference lists of papers. After removal of duplicates, the abstracts were then screened for study inclusion criteria and full-text articles then assessed for eligibility.

We included RCTs and large (more than 1,000 participants) prospective cohort studies (including those with prospectively collected data analysed retrospectively) that assessed outcomes for women with term deliveries (>37 weeks gestation) after cesarean and vaginal delivery (exposures) with follow-up of greater than or equal to one year from the index delivery.

Two assessors (OEK and SJS) independently screened titles and abstracts of studies, then accessed and appraised full texts. Data were extracted onto the RevMan programme (version 5.3) (OEK and SJS). Where available, data for outcomes following operative vaginal delivery were included in the ‘vaginal delivery’ group. In order to detect bias and to grade the quality of studies, we used the Scottish Intercollegiate Guideline Network (SIGN) Methodology checklists for cohort studies and RCTs where appropriate and graded the studies as high quality with little or no risk of bias (++), acceptable with some flaws in the study with an associated risk of bias (+), or low quality with significant flaws (0) (OEK and SJS) [15]. As an additional assessment of bias and study quality, we used the Risk of Bias Assessment tool for Non-randomized Studies (RoBANS), which has shown moderate reliability and promising validity [16]. Studies were excluded if they did not provide sufficient information to assess methods or data analysis. Authors were contacted to clarify ambiguities in published results, in particular figures for outcomes in cesarean delivery and vaginal delivery groups [17–19]. Where there was disagreement over eligibility for inclusion or assessment of study quality, this was referred to a meeting of all authors.

We analysed the data in three groups of prespecified outcomes: maternal, childhood, and subsequent pregnancy outcomes. The primary outcome chosen for each database search was that which we felt patients would be most concerned about. As there were several other relevant outcomes for each database search, we added these as secondary outcomes (see Table 1).

**Table 1:** Primary and secondary outcomes. Table displaying the primary and secondary outcomes specified for database searches of maternal, childhood, and subsequent pregnancy outcomes.

Group	Primary outcome	Secondary outcomes
**Maternal outcomes**	Pelvic floor dysfunction (any of urinary incontinence, fecal incontinence, uterine prolapse, or vaginal prolapse)	Maternal death Chronic pain (including pelvic pain) Dysmenorrhea Menorrhagia Sexual dysfunction (including dyspareunia) Healthcare usage Subfertility
**Childhood outcomes**	Asthma (up to 12 years and from 15 years)	Wheeze (up to 5 years and 6–15 years) Allergy/Atopy/Hypersensitivity/Dermatitis Overweight (3–13 years) Obesity (up to 5 years, 6–15 years, and adulthood) Inflammatory bowel disease (up to 35 years)
**Subsequent pregnancy outcomes**	Perinatal death (from 22 weeks gestation to one week of age)	Placenta previa Placenta accreta Placental abruption Uterine rupture Miscarriage Ectopic pregnancy Stillbirth Hysterectomy Postpartum haemorrhage Antepartum haemorrhage Preterm labour Fetal growth restriction (small for gestational age, low birth weight [<2,500 g]) Neonatal death

Results were pooled in a Mantel–Haenszel fixed effects meta-analysis with ORs, 95% confidence intervals, and two-sided *p*-values. Heterogeneity was assessed using the chi-squared and I-squared tests, with random effects models used when substantial heterogeneity was present, i.e., when I-squared exceeded 40%. Results were summarized in tables and illustrated using forest plots. Planned sensitivity analyses were by study quality, cohort size (>50,000), GDP of country of publication (top two thirds, bottom third of International Monetary Fund list), and study period (cohort pre-1980, post-1980) and were applied where appropriate. This study period cutoff was chosen as cesarean delivery rates and obstetric care have changed significantly since 1980.

### Post hoc protocol changes to methods

Prior to analysis, we made the following changes to our methods from the published protocol. We clarified that the definition of ‘prospective cohort study’ included studies if data had been collected prospectively, even if analysis was retrospective. We changed the threshold of heterogeneity that we would use random effects meta-analysis from chi-squared test *p*-value <0.05 to the more conservative I^2^ > 40%. We added the RoBANS tool for the assessment of bias and study quality to the use of the SIGN checklist. In addition, at the data extraction stage, we made a decision to report both ‘small for gestational age’ and ‘low birth weight’ as secondary subsequent pregnancy outcomes in our analysis rather than ‘fetal growth restriction’ as specified in our protocol.

## Results

Electronic searches provided 30,327 citations and hand-searching of references provided a further 57 papers. After exclusions, 80 studies were included (one RCT and 79 observational studies) (see flow diagrams in S1 Fig, S2 Fig and S3 Fig; of note, three of the 80 studies contributed to both the ‘maternal outcomes’ and ‘subsequent pregnancy outcomes’ meta-analyses and are included in both flowcharts; thus, the sum of all papers in flow diagrams is 83). For the purpose of combining estimates, the RCT was not meta-analysed with the observational studies, but the results were presented separately. Two independent reviewers assessed study quality. Several studies had high or unclear risk of detection bias through inadequate blinding of outcome assessments, and many had a high risk of attrition bias caused by the inadequate handling of incomplete outcome data. The majority of studies were of acceptable quality, and many were adjusted for multiple confounding factors. Of note, in the majority of studies, the adjusted ORs were not substantially different from the crude ORs. All studies were from high-income countries (top third of GDP list); 13 were hospital studies, and 67 were population studies (see S2 Table, S3 Table, S4 Table and S5 Table).

**Fig 1:**
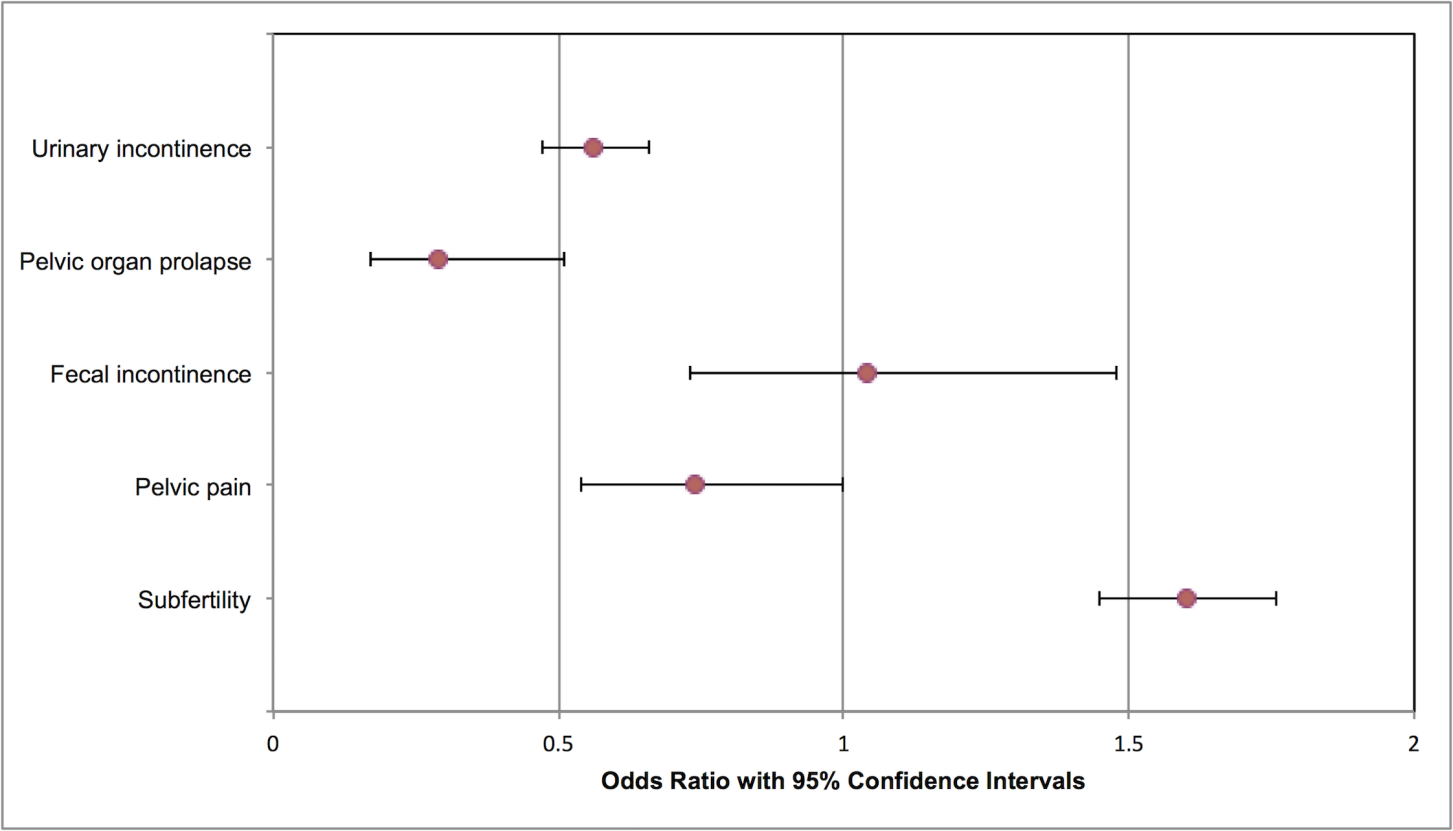
Modified forest plot of maternal outcomes meta-analyses. In addition to the meta-analyses shown, one RCT assessed dysmenorrhea and menorrhagia with no statistically significant associations. One RCT and one cohort study investigated sexual dysfunction, notably dyspareunia, with conflicting results. No studies investigated maternal death or healthcare usage. RCT, randomized controlled trial.

**Fig 2:**
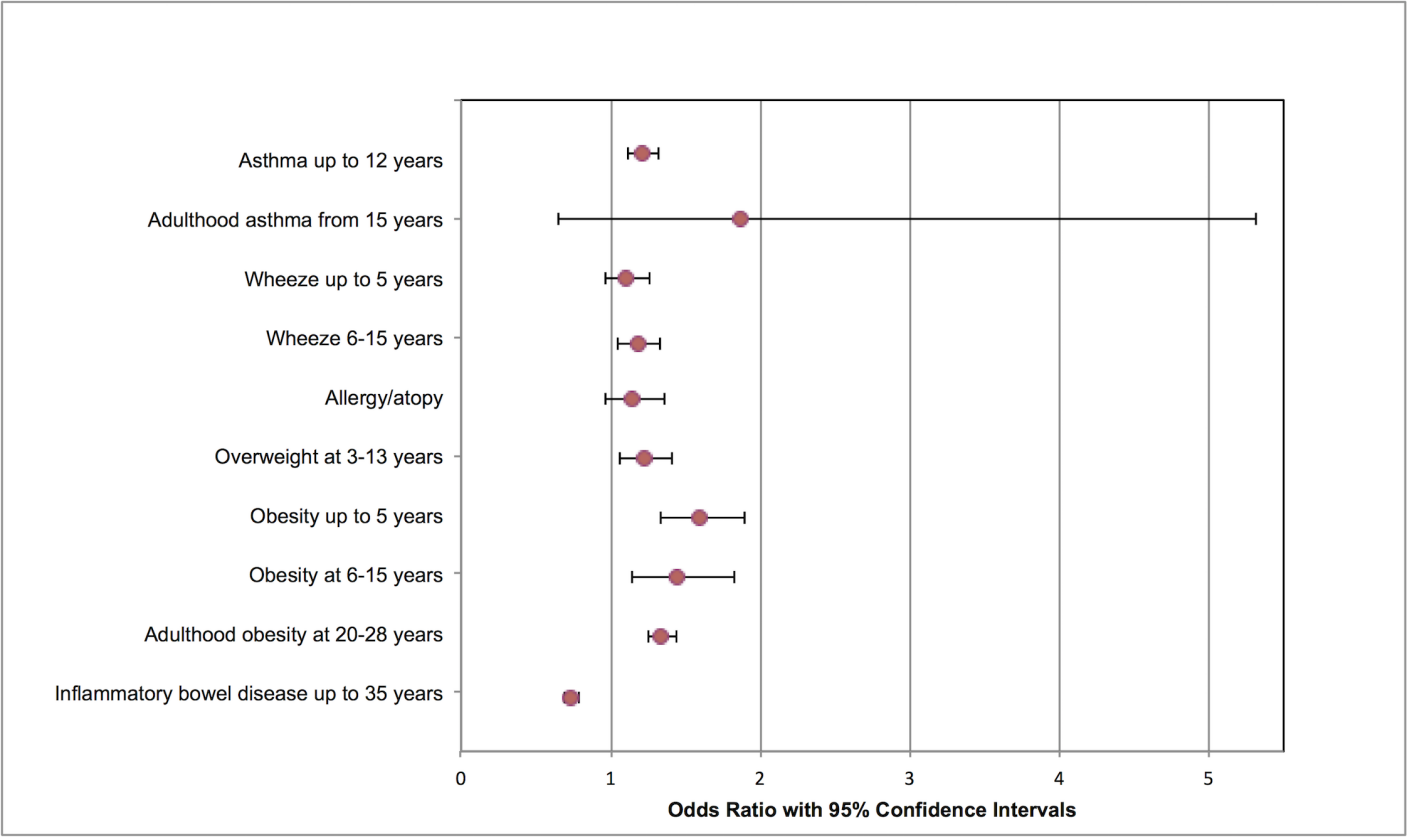
Modified forest plot of childhood outcomes meta-analyses. As studies had multiple cohorts and different follow-up periods, meta-analyses were divided according to age or duration of follow-up.

**Fig 3:**
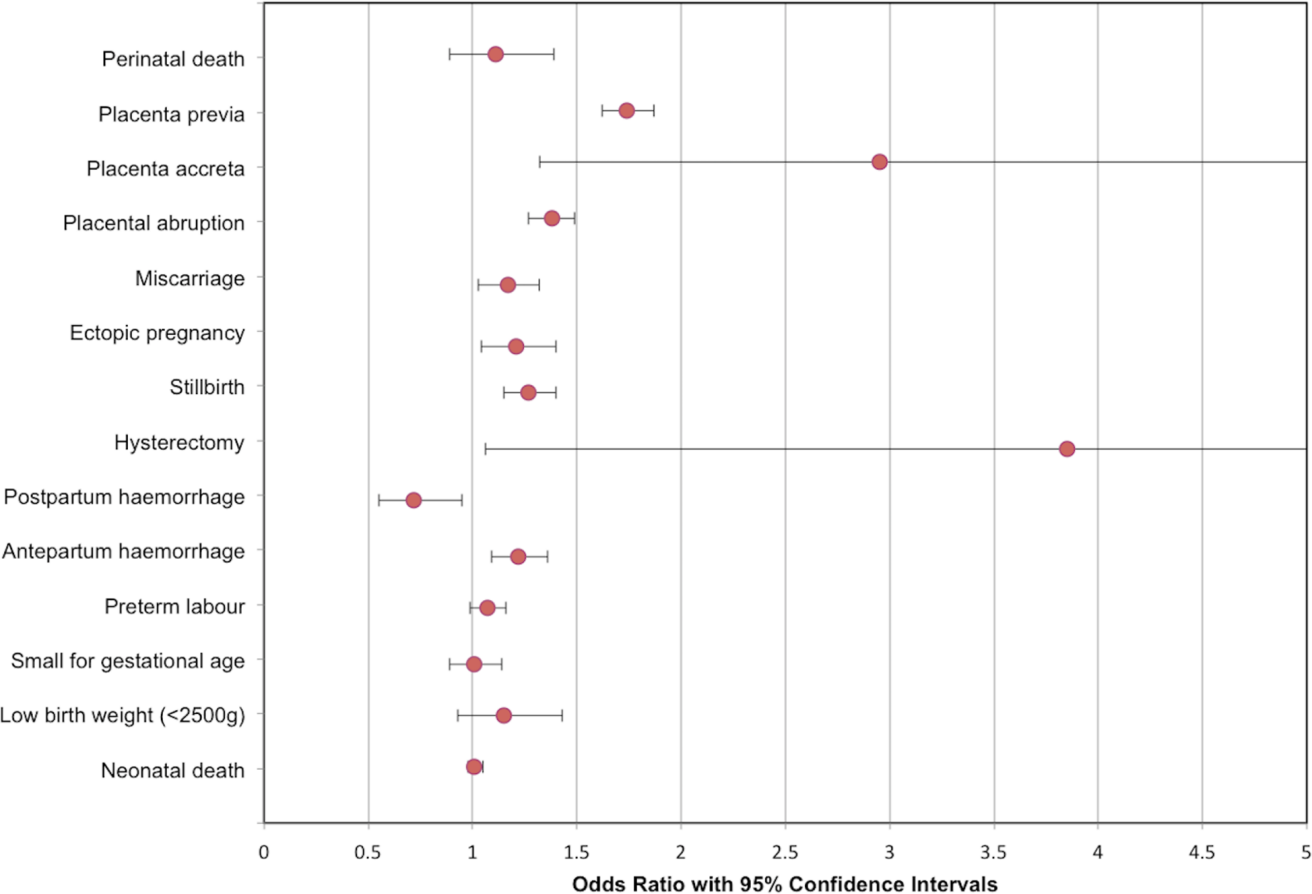
Modified forest plot of subsequent pregnancy outcomes meta-analyses. An additional outcome not included in this modified forest plot is uterine rupture, OR 25.81 (95% confidence intervals 10.96 to 60.76). OR, odds ratio.

Results of meta-analyses are summarized in Table 2 and Figs 1–3.

**Table 2:** Summary of meta-analyses. Table summarizing the meta-analyses performed detailing the number of studies, number of participants, effect estimate of each outcome and statistical method used. As studies had multiple cohorts and different follow-up periods, meta-analyses were divided according to age or duration of follow-up.

Outcome	Studies	Participants	OR [95%CI]	Statistical Method
***Maternal Outcomes***				
**Primary Outcomes**				
Urinary incontinence	8	58900	0.56 [0.47, 0.66]	Mantel-Haenszel Random Effects
Pelvic organ prolapse	2	39208	0.29 [0.17, 0.51]	Mantel-Haenszel Random Effects
Fecal incontinence	5	43260	1.04 [0.73, 1.48]	Mantel-Haenszel Random Effects
**Secondary Outcomes**				
Pelvic pain	2	18308	0.74 [0.54, 1.00]	Mantel-Haenszel Fixed Effects
Subfertility	11	3692014	1.60 [1.45, 1.76]	Mantel-Haenszel Random Effects
***Childhood Outcomes***				
**Primary Outcomes**				
Asthma up to 12 years	13	887960	1.21 [1.11, 1.32]	Mantel-Haenszel Random Effects
Adulthood asthma from 15 years	2	9072	1.87 [0.65, 5.32]	Mantel-Haenszel Random Effects
**Secondary Outcomes**				
Wheeze up to 5 years	5	53686	1.10 [0.96, 1.26]	Mantel-Haenszel Random Effects
Wheeze 6-15 years	4	20815	1.18 [1.05, 1.33]	Mantel-Haenszel Fixed Effects
Allergy/atopy	8	44131	1.15 [0.97, 1.36]	Mantel-Haenszel Random Effects
Overweight at 3-13 years	4	187148	1.22 [1.06, 1.41]	Mantel-Haenszel Random Effects
Obesity up to 5 years	6	64113	1.59 [1.33, 1.90]	Mantel-Haenszel Random Effects
Obesity at 6-15 years	5	35428	1.45 [1.15, 1.83]	Mantel-Haenszel Random Effects
Adult obesity at 20-28 years	5	33101	1.34 [1.25, 1.44]	Mantel-Haenszel Fixed Effects
Inflammatory bowel disease up to 35 years	3	2605129	0.73 [0.69, 0.79]	Mantel-Haenszel Fixed Effects
***Subsequent Pregnancy Outcomes***				
**Primary Outcome**				
Perinatal death	2	91429	1.11 [0.89, 1.39]	Mantel-Haenszel Fixed Effects
**Secondary Outcomes**				
Placenta previa	10	7101692	1.74 [1.62, 1.87]	Mantel-Haenszel Random Effects
Placenta accreta	3	705108	2.95 [1.32, 6.60]	Mantel-Haenszel Random Effects
Placental abruption	6	5667160	1.38 [1.27, 1.49]	Mantel-Haenszel Random Effects
Uterine rupture	4	841209	25.81 [10.96, 60.76]	Mantel-Haenszel Random Effects
Miscarriage	4	151412	1.17 [1.03, 1.32]	Mantel-Haenszel Random Effects
Ectopic pregnancy	3	312026	1.21 [1.04, 1.40]	Mantel-Haenszel Fixed Effects
Stillbirth	8	703562	1.27 [1.15, 1.40]	Mantel-Haenszel Fixed Effects
Hysterectomy	2	167674	3.85 [1.06, 14.02]	Mantel-Haenszel Random Effects
Antepartum hemorrhage	3	116073	2.43 [0.81, 7.34]	Mantel-Haenszel Fixed Effects
Postpartum hemorrhage	2	167674	0.72 [0.55, 0.95]	Mantel-Haenszel Random Effects
Preterm labor	7	10509366	1.07 [0.99, 1.16]	Mantel-Haenszel Random Effects
Small for gestational age	5	10901970	1.01 [0.89, 1.14]	Mantel-Haenszel Random Effects
Low birth weight (<2500g)	4	699499	1.15 [0.93, 1.43]	Mantel-Haenszel Random Effects
Neonatal death	5	10275127	1.01 [0.98, 1.05]	Mantel-Haenszel Fixed Effects

**Abbreviations:** OR = Odds Ratio; CI = Confidence Interval.

### Maternal outcomes

One RCT of 2,088 participants [5] and data from 23 reports of prospective cohort studies (total of 3,849,075 participants) were included [20–42] (see S2 Table for characteristics).

#### Primary outcome: Pelvic floor dysfunction

No studies reported ‘pelvic floor dysfunction’ as an outcome; therefore, the following individual outcomes were used: urinary incontinence, pelvic organ prolapse (to include uterine and/or vaginal prolapse), and fecal incontinence. The RCT did not demonstrate any statistically significant association of cesarean delivery with urinary incontinence (OR 0.78, 95% confidence intervals 0.56 to 1.08) or fecal incontinence (OR 3.07, 95% confidence intervals 0.90 to 10.49) [5]. In total, data from 11 manuscripts were eligible for meta-analysis, with follow-up ranging from 12 months postnatal to age 80 years [5,20,22,25–28,32,38,39,42,43]. Compared to vaginal delivery, cesarean delivery was associated with reduced odds of urinary incontinence (1,024/7,306 cesarean delivery versus 7,713/51,594 vaginal delivery; OR 0.56, 95% confidence intervals 0.47 to 0.66, *p* < 0.000011; I^2^ = 71%; 8 studies) (S4 Fig) [5,20,25,28,32,33,38,39,42]. Similar results were seen when sensitivity analysis was performed, excluding two low-quality studies [32,39] (955/6,883 cesarean delivery versus 7,129/49,319 vaginal delivery; OR 0.59, 95% confidence intervals 0.49 to 0.70, *p* < 0.00001; I^2^ = 72%; 6 studies).

Compared to vaginal delivery, cesarean delivery was associated with reduced odds of pelvic organ prolapse (116/4,898 cesarean delivery versus 2,055/34,310 vaginal delivery; OR 0.29, 95% confidence intervals 0.17 to 0.51, *p* = 0.005, I^2^ = 87%; 2 studies) (S5 Fig) [20,27]. There was no statistically significant difference in rates of fecal incontinence (234/6,449 cesarean delivery versus 705/36,811 vaginal delivery; OR 1.04, 95% confidence intervals 0.73 to 1.48, *p* = 0.82, I^2^ = 72%; 5 studies) (S6 Fig) [5,20,22,26,33,42]. Similar results were seen when sensitivity analysis was performed, excluding one low-quality study [22] (187/6,087 cesarean delivery versus 663/36,534 vaginal delivery; OR 1.09, 95% confidence intervals 0.71 to 1.67, *p* = 0.69, I^2^ = 77%; 4 studies).

#### Secondary outcomes: Menorrhagia and dysmenorrhea; chronic pain (including pelvic pain) and sexual dysfunction (including dyspareunia); and subfertility

Data from the one RCT showed no association between mode of delivery and heavy menstrual bleeding (menorrhagia) or painful menstrual bleeding (dysmenorrhea) [5].

Two studies investigated pelvic pain [21,42]. There was no statistically significant association of mode of delivery with pelvic pain (33/2,449 cesarean delivery versus 313/15,512 vaginal delivery; OR 0.74, 95% confidence intervals 0.54 to 1.00, *p* = 0.05, I^2^ = 0%) (S7 Fig).

When compared with vaginal delivery, cesarean delivery was associated with increased odds of dyspareunia in one cohort study (OR 1.49, 95% confidence intervals 1.11 to 2.00) [34], but there was no statistically significant effect demonstrated in the RCT (OR 0.96, 95% confidence intervals 0.61 to 1.50) [5].

There were no studies found investigating maternal death or healthcare usage as a long-term association of cesarean delivery.

Meta-analysis of 11 studies (3,692,014 women) showed an association between cesarean delivery and increased odds of subfertility when compared to vaginal delivery (246,096/567,155 previous cesarean delivery versus 995,022/3,124,859 previous vaginal delivery; OR 1.60, 95% confidence intervals 1.45 to 1.76, *p* < 0.00001) (S8 Fig) [23,24,29–31,35–37,40,41]. Between-study heterogeneity was high in this meta-analysis (I^2^ = 99%) due to the varying follow-up periods, varying cohort numbers, and study periods. Sensitivity analysis excluding four studies with <50,000 participants [29,30,35,36] did not alter these results (243,260/560,190 previous cesarean delivery versus 978,990/3,075,271 previous vaginal delivery; OR 1.64, 95% confidence intervals 1.46 to 1.84, *p* < 0.00001; I^2^ = 100%; 7 studies).

### Childhood outcomes

Thirty-five manuscripts met the inclusion criteria (see S3 Table for characteristics) [17,19,44–76]. As studies had multiple cohorts and different follow-up periods, meta-analyses were divided according to age or duration of follow-up.

#### Primary outcome: Asthma

Meta-analysis of 13 studies (887,960 participants) [17,45,49,55,58,59,61,63,67,69,72,73,76] showed an association between cesarean delivery and increased odds of asthma in children aged up to 12 years compared to vaginal delivery (4,788/124,668 cesarean delivery versus 23,308/763,292 vaginal delivery; OR 1.21, 95% confidence intervals 1.11 to 1.32, *p* < 0.00001) (S9 Fig). There was significant heterogeneity between the studies (I^2^ = 75%). Planned sensitivity analysis excluding the single low-quality study [72] did not change findings (4,743/124,068 cesarean delivery versus 23,092/760,142 vaginal delivery; OR 1.22, 95% confidence intervals 1.11 to 1.33, *p* < 0.0001; I^2^ = 77%). Cesarean delivery was associated with increased risk of childhood asthma in another study that could not be included in the meta-analysis because results were not subdivided by duration of follow up [71]. Two studies (9,072 participants) investigated the development of adulthood asthma in children delivered by cesarean section (from 15 years) [74,75], and no statistically significant association between cesarean delivery and adulthood asthma was seen, although one of these studies was graded as low quality [74]; excluding this study changed the association to an increased odds of adulthood asthma following cesarean delivery (OR 3.31, 95% confidence intervals 1.81 to 6.05) (S10 Fig).

#### Secondary outcomes: Wheeze; hypersensitivity/dermatitis/allergy/atopy; overweight/obesity; and inflammatory bowel disease

There was no statistically significant association of mode of delivery with the development of childhood wheeze at up to 5 years [58,62,63,72], but at 6–15 years follow-up, cesarean delivery was associated with increased odds of wheeze in children when compared with those delivered vaginally (416/3,450 cesarean delivery versus 1,603/17,365 vaginal delivery; OR 1.18, 95% confidence intervals 1.05 to 1.33, *p* = 0.006, I^2^ = 0%) (S11 Fig, S12 Fig) [59,62,69,72]. Following sensitivy analysis, excluding two low-quality studies [59,72], there was no statistically significant association between mode of delivery and wheeze at this age (251/1,848 cesarean delivery versus 640/6,318 vaginal delivery; OR 1.14, 95% confidence intervals 0.97 to 1.34; *p* = 0.11, I^2^ = 0%).

Eight studies (*n* = 44,131) assessed allergies, hypersensitivity, dermatitis, or atopic conditions, evaluating a variety of outcomes [51,59,61,63,67,69,75,77]. In order to enable a meta-analysis, a single outcome from each study was chosen. All studies had follow-up of up to 8 years except one [75], which had 31 years follow-up. There was no statistically significant association between mode of delivery and odds of hypersensitivity/allergy/dermatitis/atopy in the meta-analysis (S13 Fig). There was moderate heterogeneity between the studies (I^2^ = 51%).

Compared with vaginal delivery, cesarean delivery was associated with increased odds of childhood overweight (3,221/39,866 cesarean delivery versus 9,792/147,282 vaginal delivery; OR 1.22, 95% confidence intervals 1.06 to 1.41, *p* = 0.007; 4 studies; I^2^ = 47%) [56,57,64,70]. In performing planned sensitivity analyses, we excluded one low-quality study [70], which did not alter results (3,191/39,721 cesarean delivery versus 9,587/145,740 vaginal delivery; OR 1.19, 95% confidence intervals 1.04 to 1.35; *p* = 0.01, I^2^ = 42%). Cesarean delivery was also associated with increased odds of childhood obesity at up to 5 years when compared with vaginal delivery (834/6,645 cesarean delivery versus 5,295/57,468 vaginal delivery; OR 1.59, 95% confidence intervals 1.33 to 1.90, *p* < 0.00001, I^2^ = 68%; 6 cohorts) [17,19,54,64], at 6–15 years (655/5,728 cesarean delivery versus 2,716/29,700 vaginal delivery; OR 1.45, 95% confidence intervals 1.15 to 1.83, *p* = 0.002, I^2^ = 63%; 5 cohorts) [19,44,53,64], and at 20–28 years (1,250/7,759 cesarean delivery versus 3,105/25,342 vaginal delivery; OR 1.34, 95% confidence intervals 1.25 to 1.44, *p* < 0.0001, I^2^ = 0%; 5 studies) [19,48,53,60,66] (S14 Fig, S15 Fig, S16 Fig, and S17 Fig).

In a meta-analysis of 3 studies, cesarean delivery was associated with reduced odds of inflammatory bowel disease when compared with vaginal delivery (878/319,164 cesarean delivery versus 7,806/2,285,965 vaginal delivery; OR 0.73, 95% confidence intervals 0.69 to 0.79, *p* < 0.00001, I^2^ = 0%) (S18 Fig) [10,17,68].

### Subsequent pregnancy outcomes

There were 24 cohort studies assessing outcomes for pregnancy following cesarean delivery (see S4 Table for characteristics) [29,35,40,78–98].

#### Primary outcome: Perinatal death

The primary outcome of perinatal death (defined as the combination of stillbirth [as defined by the authors] and neonatal death [as defined by the authors]) was assessed in 2 studies (*n* = 91,429) [81,86,90,91,94,97]. There was no statistically significant association of mode of delivery with perinatal mortality (98/17,259 previous cesarean delivery versus 385/74,170 previous vaginal delivery; OR 1.11, 95% confidence intervals 0.89 to 1.39, *p* = 0.22; I^2^ = 34%) (S19 Fig).

#### Secondary outcomes

Women with previous cesarean delivery had increased odds of having placenta previa compared to women with a previous vaginal delivery (5,039/1,025,692 previous cesarean delivery versus 16,679/6,076,000 previous vaginal delivery; OR 1.74, 95% confidence intervals 1.62 to 1.87, *p* < 0.00001; I^2^ = 55%; 10 studies) (S20 Fig) [79,80,82,84–89,95]. Similar results were seen when prespecified sensitivity analysis was performed, omitting studies of <50,000 participants (OR 1.73, 95% confidence intervals 1.59 to 1.88, *p* < 0.00001; I^2^ = 68%) [80,85,86]. When pre-1980 cohorts were omitted, there was little impact on results (OR 1.77, 95% confidence intervals 1.62 to 1.94, *p* < 0.00001; I^2^ = 64%) [79,88,95].

Women with previous cesarean delivery also had increased odds of having placenta accreta compared to women with a previous vaginal delivery (44/66,241 previous cesarean delivery versus 188/638,867 previous vaginal delivery; OR 2.95, 95% confidence intervals 1.32 to 6.60, *p* = 0.008; I^2^ = 47%; 3 studies) (S21 Fig) [79,85,86,88,95]. In a sensitivity analysis excluding one study with a pre-1980 cohort [79], the association was no longer statistically significant (OR 5.32, 95% confidence intervals 0.67 to 44.26; *p* = 0.11, I^2^ = 68%).

When compared with women with previous vaginal delivery, women with a previous cesarean delivery also had increased odds of placental abruption (6,047/858,208 previous cesarean delivery versus 23,855/4,808,952 previous vaginal delivery; OR 1.38, 95% confidence intervals 1.27 to 1.49, *p* < 0.00001; I^2^ = 54%; 6 studies) [82,85–87,89,95] and uterine rupture (215/91,837 previous cesarean delivery versus 56/749,372 previous vaginal delivery; OR 25.81, 95% confidence intervals 10.96 to 60.76, *p* < 0.00001; I^2^ = 80%; 4 studies) (S22 Fig, S23 Fig) [79,85,86,97].

When compared with women with previous vaginal delivery, women with previous cesarean delivery had increased odds of miscarriage (2,060/19,106 previous cesarean delivery versus 12,663/132,306 previous vaginal delivery; OR 1.17, 95% confidence intervals 1.03 to 1.32, *p* = 0.01; I^2^ = 79%; 4 studies) [29,35,40,85], ectopic pregnancy (223/71,040 previous cesarean delivery versus 772/240,986 previous vaginal delivery; OR 1.21, 95% confidence intervals 1.04 to 1.40, *p* = 0.02; I^2^ = 0%; 3 studies) [35,78,85], and stillbirth (496/118,192 previous cesarean delivery versus 1,905/585,370 previous vaginal delivery; OR 1.27, 95% confidence intervals 1.15 to 1.40, *p* < 0.00001; I^2^ = 34%; 8 studies) (S24 Fig, S25 Fig, S26 Fig) [83,85,86,92,93,96–98].

Women with previous cesarean delivery had increased odds of hysterectomy (19/29,626 previous cesarean delivery versus 31/138,048 previous vaginal delivery; OR 3.85, 95% confidence intervals 1.06 to 14.02, *p* = 0.04; I^2^ = 69%; 2 studies) [85,97] and antepartum haemorrhage (413/17,259 previous cesarean delivery versus 1,237/74,170 previous vaginal delivery; OR 1.22, 95% confidence intervals 1.09 to 1.36, *p* = 0.0007; I^2^ = 0%; 2 studies) [86,90] but reduced odds of postpartum haemorrhage (1,087/29,626 previous cesarean delivery versus 7,455/138,048 previous vaginal delivery; OR 0.72, 95% confidence intervals 0.55 to 0.95, *p* = 0.02; I^2^ = 88%; 2 studies) [85,97] (S27 Fig, S28 Fig, S29 Fig). There was no statistically significant association between previous mode of delivery and preterm labour [85,86,90,91,94,97,98], small for gestational age [79,86,91,94,97], low birth weight (<2,500 g) [86,90,94,98] or neonatal death [81,86,91,94,97] (S30 Fig, S31 Fig, S32 Fig, S33 Fig).

### Non-prespecified outcomes

Whilst searching for the outcomes defined in our protocol, we identified studies looking at the risk of additional outcomes, including childhood type 1 diabetes [17,99–102] and celiac disease [99,103]. These were not defined as outcome variables in our protocol, and we did not therefore systematically review the risks of these events. However, the results of these studies are summarized in S6 Table.

## Discussion

This systematic review and meta-analysis has highlighted the long-term risks and benefits of cesarean delivery for mother, baby, and subsequent pregnancies when compared to vaginal delivery in term (>37 weeks gestation) pregnancies. We found that cesarean delivery is associated with reduced rates of urinary incontinence and pelvic organ prolapse but has adverse associations with fertility, future pregnancy outcome, future pregnancy complications, and long-term childhood outcomes.

We attempted to minimize bias in the review by adhering to a registered protocol and following the MOOSE guidelines [14]. We only included studies with a large number of participants. In order to minimize publication bias, the database searches were comprehensive, without language or date restrictions, and efforts were made to include unpublished data through contacting authors. However, as with all systematic reviews, publication bias is a possibility. Despite the strengths of this systematic review, we recognize that the associations are based on predominantly observational data, which itself may be vulnerable to bias.

We chose our outcomes a priori. Whilst this minimized bias, we have been unable to include some data from well-conducted prospective randomized trials. Examples include [6] and [104], both of which looked at neurodevelopmental outcomes at two years of age in children delivered by planned cesarean delivery versus planned vaginal delivery. Neither study demonstrated statistically significant differences in the two delivery groups; therefore, including these would not have substantially altered the conclusions of our review.

Two independent reviewers assessed study quality using two bias assessment tools that correlated well. Any bias was mainly due to attrition bias or detection bias. These biases are likely to have operated in different directions, with attrition bias reducing the observed difference between the treatment groups and detection bias magnifying it. Importantly, excluding studies of low quality did not change findings, suggesting that any bias will have had minimal effect. However, as with all meta-analyses of observational studies, some caution must be exercised in the interpretation of results. This is especially true in analyses where high levels of between-study heterogeneity were observed (pelvic organ prolapse, subfertility, placenta previa, uterine rupture, preterm labour), likely to reflect differences in the definitions of outcomes and confounders, follow-up times, and parity in cohorts, or where there the range of confidence intervals were very wide (placenta accreta, uterine rupture, hysterectomy, antepartum haemorrhage).

Observational studies of the risks and benefits of cesarean delivery have multiple potential confounding factors. The majority of included studies adjusted for at least some of these (S2 Table, S3 Table, S4 Table). Maternal age, parity, and BMI were commonly adjusted-for variables. Studies assessing childhood outcomes frequently also adjusted for birth weight, breastfeeding, maternal education, and maternal smoking. Studies assessing the association of cesarean delivery with subsequent pregnancy outcomes additionally adjusted for a range of maternal complications in previous pregnancy such as hypertension, diabetes and preterm labour. In this systematic review and summary meta-analysis of mainly observational data we were unable to adjust for confounding factors. However, it is worth noting that in the majority of studies included, multivariable analysis did not significantly alter findings of univariable analysis. Nevertheless, our findings must be interpreted with caution.

We were unable to analyse results by the indication for cesarean delivery or the category of cesarean delivery—planned (elective) or emergency. Nevertheless, several studies did assess outcomes by classification of cesarean delivery (elective or emergency) or timing of cesarean delivery (pre-labour, intrapartum, or second stage of labour) without significant changes in the ORs of complications [25,27,54,56,58,60,71]. Cesarean delivery rates varied depending on the country where the study was performed and the cohort dates; for example, the [75] study cohort in 1966 had a cesarean delivery rate of 5%. This may affect generalizability of the findings to modern practice, but temporal differences in obstetric practice are unavoidable in studies of long-term complications.

Although previous systematic reviews have assessed individual outcomes [8–12,101,105–109], we have found no other published reviews synthesizing the evidence for all long-term risks and benefits of cesarean delivery relating to mother, baby, and subsequent pregnancies. There is a lack of documented evidence about medium- to long-term outcomes in women and their babies after a planned cesarean delivery or a planned vaginal birth [4]. Therefore, the findings of this review will form a valuable and necessary addition to discussions about mode of delivery and consenting for planned cesarean delivery. Patients may attribute different weight to the outcomes; for example, some might prioritize minimizing the risk of stillbirth in a future pregnancy, while others might prioritize minimizing the risk of respiratory morbidity for their baby. The information included in this review will allow women (and their caregivers) to make more personally relevant decisions.

Although we cannot conclude that cesarean delivery causes certain outcomes, patients and clinicians should be aware that cesarean delivery is associated with long-term risks for the baby and for subsequent pregnancies and a reduced risk of urinary incontinence and pelvic organ prolapse for the mother. The significance that women attribute to these individual risks is likely to vary, but it is imperative that clinicians take care to ensure that women are made aware of any risk that they are likely to attach significance to. Women and clinicians thus should be aware of both the short- and long-term risks and benefits of cesarean delivery and discuss these when deciding on mode of delivery.

If the associations between cesarean delivery and outcomes were known to be causal, the key significant associations in this review could be summarized using ‘numbers needed to treat (NNT) for benefit or harm’. We have calculated the NNT for benefit and harm for each statistically significant outcome from the meta-analyses and displayed this in S7 Table. These are aimed to help put the risks and benefits of cesarean delivery into context and could be used as a basis for a tool to help counselling and consenting for cesarean delivery in the antenatal period, keeping in mind these figures are based on observational data. The estimates suggest that around 17 cesareans would be needed to prevent one case of urinary incontinence (NNT for benefit 17 95% CI 14,22), but for every 1,500 cesareans performed, there would be approximately nine additional cases of childhood asthma (NNT for harm 162 95% CI 107-308), and in subsequent pregnancies, an additional 166 women with subfertility (NNT for harm 9 95% CI 8-12), three women with placenta praevia (NNT for harm 494 95% CI 420, 589), two women with uterine rupture (NNT for harm 538 95% CI 224-1340), 21 miscarriages (NNT for harm 69 95% CI 37-386), and one stillbirth (NNT for harm 1144 95% CI 773-2059).

## Conclusion

We have synthesised the evidence for the long-term risks and benefits of cesarean section. This information should help inform discussions about mode of delivery and may facilitate appropriate personalized delivery planning and shared decision-making. Further research into the long-term risks and benefits of cesarean delivery on maternal request will be beneficial. Whilst randomized trials might be the gold standard in this regard, one that addressed all relevant outcomes would have to be so large and with such a long follow-up so as to be likely to be unfeasible.

## Supporting information

S1 Protocol (PDF)

S1 Moose Checklist (DOC)

S1 Table Search strategy. (DOCX)

S2 Table Maternal outcomes—Study characteristics. (DOCX)

S3 Table Childhood outcomes—Study characteristics. (DOCX)

S4 Table Subsequent pregnancy outcomes—Study characteristics. (DOCX)

S5 Table Risk of bias assessment tool for Non-randomized studies. (DOCX)

S6 Table Non-prespecified childhood outcomes after cesarean delivery compared to vaginal delivery. (DOCX)

S7 Table Summary of associations and numbers needed to treat for benefit or harm. (DOCX)

S1 Fig Study flow diagram of maternal outcomes database search. (DOCX)

S2 Fig Study flow diagram of childhood outcomes database search. (DOCX)

S3 Fig Study flow diagram of subsequent pregnancy outcomes database search. (DOCX)

S4 Fig A random effects meta-analysis of urinary incontinence after cesarean delivery compared to vaginal delivery. (PDF)

S5 Fig A random effects meta-analysis of pelvic organ prolapse after cesarean delivery compared to vaginal delivery. (PDF)

S6 Fig A random effects meta-analysis of fecal incontinence after cesarean delivery compared to vaginal delivery. (PDF)

S7 Fig A fixed effects meta-analysis of pelvic pain after cesarean delivery compared to vaginal delivery. (PDF)

S8 Fig A random effects meta-analysis of no further pregnancy up to 28 years after cesarean delivery compared with vaginal delivery. (PDF)

S9 Fig A random effects meta-analysis of asthma in children up to 12 years old after cesarean delivery compared to vaginal delivery. (PDF)

S10 Fig A random effects meta-analysis of asthma in adults after cesarean delivery compared to vaginal delivery. (PDF)

S11 Fig A random effects meta-analysis of wheeze in children up to 5 years old after cesarean delivery compared to vaginal delivery. (PDF)

S12 Fig A fixed effects meta-analysis of wheeze in children 6–15 years old after cesarean delivery compared to vaginal delivery. (PDF)

S13 Fig A random effects meta-analysis of hypersensitivity or allergy or dermatitis or atopy in children after cesarean delivery compared to vaginal delivery. (PDF)

S14 Fig A random effects meta-analysis of children being overweight at 3–8 years old after cesarean delivery compared to vaginal delivery. (PDF)

S15 Fig A random effects meta-analysis of obesity in children up to 5 years old after cesarean delivery compared to vaginal delivery. (PDF)

S16 Fig A random effects meta-analysis of obesity in children 6–15 years old after cesarean delivery compared to vaginal delivery. (PDF)

S17 Fig A fixed effects meta-analysis of adulthood obesity after cesarean delivery compared to vaginal delivery. (PDF)

S18 Fig A fixed effects meta-analysis of inflammatory bowel disease in children and adults up to age 35 years after cesarean delivery compared to vaginal delivery. (PDF)

S19 Fig A fixed effects meta-analysis of perinatal death in pregnancy after cesarean delivery compared to pregnancy after vaginal delivery. (PDF)

S20 Fig A random effects meta-analysis of placenta previa in pregnancy after cesarean delivery compared to pregnancy after vaginal delivery. (PDF)

S21 Fig A random effects meta-analysis of placenta accreta in pregnancy after cesarean delivery compared to pregnancy after vaginal delivery. (PDF)

S22 Fig A random effects meta-analysis of placental abruption in pregnancy after cesarean delivery compared to pregnancy after vaginal delivery. (PDF)

S23 Fig A random effects meta-analysis of uterine rupture in pregnancy after cesarean delivery compared to pregnancy after vaginal delivery. (PDF)

S24 Fig A random effects meta-analysis of miscarriage in pregnancy after cesarean delivery compared to pregnancy after vaginal delivery. (PDF)

S25 Fig A fixed effects meta-analysis of ectopic pregnancy after cesarean delivery compared to pregnancy after vaginal delivery. (PDF)

S26 Fig A fixed effects meta-analysis of stillbirth in pregnancy after cesarean delivery compared to pregnancy after vaginal delivery. (PDF)

S27 Fig A random effects meta-analysis of hysterectomy in pregnancy after caesarean delivery compared to pregnancy after vaginal delivery. (PDF)

S28 Fig A fixed effects meta-analysis of having antepartum haemorrhage in pregnancy after cesarean delivery compared to pregnancy after vaginal delivery. (PDF)

S29 Fig A random effects meta-analysis of postpartum haemorrhage in pregnancy after cesarean delivery compared to pregnancy after vaginal delivery. (PDF)

S30 Fig A random effects meta-analysis of preterm labour in pregnancy after cesarean delivery compared to pregnancy after vaginal delivery. (PDF)

S31 Fig A random effects meta-analysis of having a small for gestational age baby in pregnancy after cesarean delivery compared to pregnancy after vaginal delivery. (PDF)

S32 Fig A random effects meta-analysis of having a baby with low birthweight (<2,500 g) in pregnancy after cesarean delivery compared to pregnancy after vaginal delivery. (PDF)

S33 Fig A fixed effects meta-analysis of neonatal death following a pregnancy after cesarean delivery compared to pregnancy after vaginal delivery. (PDF)

## Abbreviations

CINAHL: Cumulative Index to Nursing and Allied Health Literature
MOOSE: Meta-analysis Of Observational Studies in Epidemiology
NNT: numbers needed to treat
OR: odds ratio
RCT: randomized controlled trial
RoBANS: Risk of Bias Assessment tool for Non-randomized Studies
SIGN: Scottish Intercollegiate Guideline Network
