# Supplementary material for: Long-term risks and benefits associated with cesarean delivery for mother, baby, and subsequent pregnancies: Systematic review and meta-analysis
Source: PLoS Med. 2018 Jan 23;15(1):e1002494. doi: 10.1371/journal.pmed.1002494 (PMC5779640; doi:10.1371/journal.pmed.1002494)
Supplement: S1 Protocol — (PDF) [file pmed.1002494.s001.pdf]

UNIVERSITY of York
Centre for Reviews and Dissemination

NHS
National Institute for Health Research

PROSPERO International prospective register of systematic reviews

The long-term consequences of caesarean section for mother, baby and subsequent pregnancies: a systematic review and meta-analysis

Oonagh Keag, Sarah Stock, Jane Norman

Citation

Oonagh Keag, Sarah Stock, Jane Norman. The long-term consequences of caesarean section for mother, baby and subsequent pregnancies: a systematic review and meta-analysis. PROSPERO 2014:CRD42014007006 Available from http://www.crd.york.ac.uk/PROSPERO_REBRANDING/display_record.asp?ID=CRD42014007006

Review question(s)

What are the long-term consequences of caesarean section for mother, baby and subsequent pregnancies when compared to vaginal delivery?

Searches

We will search the following electronic bibliographic databases: MEDLINE, EMBASE, The Cochrane Library (Cochrane Central Register of Controlled Trials (CENTRAL), Cochrane Database of Systematic Reviews, Database of Abstracts of Reviews of Effects (DARE)) and the Cumulative Index to Nursing and Allied Health Literature (CINAHL).

The search strategy will be developed and tested by the review team in collaboration with a librarian experienced in literature searching. The search will be limited to human studies, randomised controlled trials and cohort studies. There will be no date or language restrictions. The searches will be re-run just before the final analyses and further studies retrieved for inclusion. A manual search of all appropriate references (identified as relevant from their citation within the article) cited in included studies and identified review articles will be performed. Abstracts for all studies will be obtained for assessment and inclusion will be determined using the criteria outlined below.

Types of study to be included

Inclusion will be restricted to all randomized controlled trials and large (more than 1000 participants) prospective cohort studies with a follow-up time of greater than one year following delivery.

Condition or domain being studied

Rates of caesarean section (CS) continue to rise in the UK. For nulliparous women the rate of CS is 25%, and an increasing proportion of women are being delivered by CS for maternal request when there is no medical or obstetric reason. NICE have synthesized evidence on the short-term adverse effects of CS for mother and baby and endorse the concept of CS for maternal request. However, the long-term consequences of CS for mother, baby and for subsequent pregnancies are less frequently discussed with women. Future pregnancy risks include increased rates of placenta praevia, accreta, uterine rupture and stillbirth, and for children born by CS, increased rates of obesity and asthma. The aim of this review is to bring all the evidence together so that clinicians can provide appropriate information for women to make an informed decision about CS.

References:

Bragg F et al. Variation in rates of caesarean section among English NHS trusts after accounting for maternal and clinical risk: cross sectional study. BMJ 2010; 341: 5065

RCOG Clinical Effectiveness Support Unit. National Sentinel Caesarean Section Audit Report. London: RCOG; 2001

Gurol-Urganci I et al. Risk of placenta previa in second birth after first birth cesarean section: a population-based study and meta-analysis. BMC Pregnancy Childbirth 2011; 11: 95

Jauniaux E, Jurkovic K. Placenta accrete: pathogenesis of a 20th century iatrogenic uterine disease. Placenta 2012; 33(4): 244-51

Fitzpatrick KE et al. Uterine rupture by intended mode of delivery in the UK: a national case-control study. PLoS Med 2012; 9(3): e1001184

Smith GC, Pell JP, Dobbie R. Caesarean section and risk of unexplained stillbirth in subsequent pregnancy. Lancet 2003; 362(9398): 1779-84

Huh SY et al. Delivery by caesarean section and risk of obesity in preschool age children: a prospective cohort study. Arch Dis Child Fetal Neonatal Ed 2012

Magnus MC et al. Delivery by cesarean section and early childhood respiratory symptoms and disorders: the Norwegian mother and child cohort study. Am J Epidemiol 2011; 174 (11): 1275-85

Participants/ population

Women with a delivery at term (>37 weeks gestational age) with outcomes assessed in one or more of the following categories:

1. Maternal outcomes at 1 year or more following delivery.

2. Childhood outcomes at 1 year or more following delivery.

3. Outcomes in subsequent pregnancies (to the end of the neonatal period).

Intervention(s), exposure(s)

Delivery by caesarean section.

Comparator(s)/ control

Vaginal delivery.

Context

The review will include all eligible studies identified that report long-term outcomes ie those occurring more than 12 months after the index delivery. Three areas will be looked at:

1. Long-term maternal outcomes after caesarean section.

2. Long-term outcomes in children delivered by caesarean section.

3. Outcomes in subsequent pregnancy after caesarean section.

Outcome(s)

Primary outcomes

For mother: Pelvic floor dysfunction (any of urinary incontinence, fecal incontinence, uterine prolapse, vaginal prolapse).

For baby: Asthma.

For subsequent pregnancies: Perinatal death.

Secondary outcomes

For mother: Death, chronic pain, dysmenorrhoea, menorrhagia, sexual dysfunction, healthcare usage, subfertility.

For baby: Obesity, atopy, wheeze, hypersensitivity, dermatitis, inflammatory bowel disease.

For subsequent pregnancies: placenta praevia, placenta accreta, placental abruption, uterine rupture, hysterectomy,

antepartum haemorrhage, postpartum haemorrhage, stillbirth, miscarriage, ectopic pregnancy, fetal growth restriction, preterm labour.

Data extraction, (selection and coding)
Titles and abstracts (where available) of studies identified from the initial searches will be independently assessed by two reviewers for possible inclusion [OK and SS]. The full text of all potentially eligible studies that have been identified will then be appraised by two assessors independently [OK and SS]. Where there is disagreement over eligibility for inclusion, this will be referred to a meeting of all authors. Data will be extracted independently onto the RevMan programme by both assessors [OK and SS]. Quality of the included studies will be assessed using standard criteria, looking for potential bias [OK and SS].

The following data will be extracted for each study: study design, setting, dates and size of cohort, details of intervention (mode of delivery), exclusion criteria, recruitment and study completion rates, outcome measurements, length of follow-up, definition of the outcome used, list of confounders adjusted for.

Missing data will be requested from study authors by OK.

Risk of bias (quality) assessment
Each study will be assessed independently by OK and SS using the Scottish Intercollegiate Guidelines Network Methodology Checklist. Discrepancies will be resolved by group discussion. Studies will be ranked as good (++), fair (+) or poor (0).

Strategy for data synthesis
Data collected will be systematised into a table. All studies identified in the systematic review will be included in the table whether or not they are included in the meta-analysis. Where studies are comparable on the basis of study population, intervention and outcome measure, the results will be pooled in a fixed-effects meta-analysis, with mean differences, 95% confidence intervals and two sided p-values. Heterogeneity will be assessed using the Chi-squared test and calculation of I-squared, an estimate of the proportion of variance due to between-study heterogeneity. Where evidence of heterogeneity is present (p=value from Chi-squared test <0.05) a random effects meta-analysis will be used. Where between-study heterogeneity is very high (I-squared over 80%) and is not explained through differences in study characteristics we will re-consider whether quantitative data synthesis is appropriate.

Analysis of subgroups or subsets
Sensitivity analysis where applicable by:

1. study quality (good or fair quality only).

2. by cohort size (> 50 000).

3. by GDP of country of publication (top two thirds, bottom third of International Monetary Fund list).

4. by study period (post-1980).

Dissemination plans
The results of this review will be presented at meetings of relevant societies and interest groups. It will be written up for peer-reviewed publication.

Contact details for further information
Oonagh Keag

C/O Dr Sarah Stock

MRC Centre for Reproductive Health

Room W1.17

Queen's Medical Research Institute

Little France

Edinburgh

EH16 4SA

oonaghkeag@nhs.net

Organisational affiliation of the review
Simpson Centre for Reproductive Health, Edinburgh

Review team
Dr Oonagh Keag, Simpson Centre for Reproductive Health
Dr Sarah Stock, MRC Centre for Reproductive Health, University of Edinburgh Queen's Medical Research Institute
Professor Jane Norman, MRC Centre for Reproductive Health, University of Edinburgh Queen's Medical Research Institute

Collaborators
Mrs Sheila Fisken, University of Edinburgh

Anticipated or actual start date
31 January 2014

Anticipated completion date
31 July 2014

Funding sources/sponsors
Supported by Tommy's

Conflicts of interest
The authors are writing a Scientific Impact Paper for the Royal College of Obstetricians and Gynaecologists on this topic.

Language
English

Country
Scotland

Subject index terms status
Subject indexing assigned by CRD

Subject index terms
Cesarean Section; Female; Humans; Maternal Age; Pregnancy; Risk Factors; Surgical Procedures, Elective

Stage of review
Ongoing

Date of registration in PROSPERO
12 February 2014

Date of publication of this revision
12 February 2014

DOI
10.15124/CRD42014007006

Stage of review at time of this submission	Started	Completed
Preliminary searches	Yes	No
Piloting of the study selection process	No	No
Formal screening of search results against eligibility criteria	No	No
Data extraction	No	No
Risk of bias (quality) assessment	No	No
Data analysis	No	No

PROSPERO
International prospective register of systematic reviews
The information in this record has been provided by the named contact for this review. CRD has accepted this information in good faith and registered the review in PROSPERO. CRD bears no responsibility or liability for the content of this registration record, any associated files or external websites.
