# Supplementary material for: Long-term risks and benefits associated with cesarean delivery for mother, baby, and subsequent pregnancies: Systematic review and meta-analysis
Source: PLoS Med. 2018 Jan 23;15(1):e1002494. doi: 10.1371/journal.pmed.1002494 (PMC5779640; doi:10.1371/journal.pmed.1002494)
Supplement: S1 Table — (DOCX) [file pmed.1002494.s003.docx]

**S1 Table: Search strategy**

| **Databases searched**  Medline, Embase, Cochrane, CINAHL |
| --- |
| All searches limited to human studies. No date or language restrictions. |
| **Maternal outcomes** |
| 1. cesarean section/ or cesarean section, repeat/  2. (c?esarean adj2 section*).ti,ab.  3. 1 or 2  4. exp Urinary Incontinence/ or exp Fecal Incontinence/ or exp Urinary Incontinence, Stress/  5. menstruation disturbances/ or amenorrhea/ or dysmenorrhea/ or menorrhagia/ or oligomenorrhea/ or premenstrual syndrome/  6. Sexual Dysfunction, Physiological/  7. (follow adj2 up).ti,ab.  8. Randomized Controlled Trial/  9. cohort studies/ or follow-up studies/ or longitudinal studies/ or prospective studies/ or retrospective studies/  10. long?term.ti,ab.  11. Uterine Prolapse/  12. 7 or 8 or 9 or 10  13. 8 or 9  14. female urogenital diseases/ or genital diseases, female/ or adnexal diseases/ or salpingitis/ or oophoritis/ or pelvic inflammatory disease/ or endometritis/ or parametritis/ or endometriosis/ or infertility/ or reproductive tract infections/ or sexual dysfunction, physiological/ or dyspareunia/ or vaginismus/ or adenomyosis/ or uterine hemorrhage/ or menorrhagia/ or uterine prolapse/ or pelvic floor disorders/ or urinary fistula/ or urinary bladder fistula/ or vesicovaginal fistula/ or maternal death/  15. pelvic pain/ or dysmenorrhea/  16. exp Chronic Pain/  17. 4 or 5 or 6 or 11 or 14 or 15 or 16  18. 3 and 17  19. cohort.tw.  20. 12 or 19  21. 3 and 17 and 20 |
| **Childhood outcomes** |
| 1. cesarean section/ or cesarean section, repeat/  2. (c?esarean adj2 section*).ti,ab.  3. (follow adj2 up).ti,ab.  4. exp Randomized Controlled Trial/  5. cohort studies/ or follow-up studies/ or longitudinal studies/ or prospective studies/ or retrospective studies/  6. long?term.ti,ab.  7. cohort.tw.  8. 3 or 4 or 5 or 6 or 7  9. exp Asthma/  10. Respiratory Sounds/  11. hypersensitivity/ or hypersensitivity, delayed/ or dermatitis, allergic contact/ or dermatitis, atopic/ or food hypersensitivity/ or respiratory hypersensitivity/ or rhinitis, allergic, perennial/ or rhinitis, allergic, seasonal/ or exp urticaria/  12. Eczema/  13. Obesity/ or Pediatric Obesity/  14. Diabetes Mellitus/  15. exp Cardiovascular Diseases/  16. inflammatory bowel diseases/ or colitis, ulcerative/ or crohn disease/  17. 9 or 10 or 11 or 12 or 13 or 14 or 15 or 16  18. limit 17 to ("infant (1 to 23 months)" or "preschool child (2 to 5 years)" or "child (6 to 12 years)" or "adolescent (13 to 18 years)")  19. 1 or 2  20. 8 and 18 and 19  21. 18 and 19  22. 17 and 19  23. limit 22 to ("infant (1 to 23 months)" or "preschool child (2 to 5 years)" or "child (6 to 12 years)" or "adolescent (13 to 18 years)") |
| **Subsequent pregnancy outcomes** |
| 1. Cesarean Section/  2. c?esarean section*.ti,ab.  3. 1 or 2  4. Pregnancy/ or exp Pregnancy Outcome/  5. exp Obstetric Labor Complications/  6. placenta.ti,ab.  7. Hysterectomy/  8. exp Pregnancy Complications/  9. exp Infant, Low Birth Weight/  10. abortion, spontaneous/ or abortion, habitual/ or uterine cervical incompetence/ or embryo loss/  11. Uterine Rupture/  12. exp Pregnancy, Ectopic/  13. (miscarriage* or abort*).ti,ab.  14. Fetal Death/  15. fetal mortality/ or maternal mortality/ or perinatal mortality/  16. infant, small for gestational age/ or infant, very low birth weight/ or infant, extremely low birth weight/ or infant, premature/ or infant, extremely premature/  17. 4 or 5 or 6 or 7 or 8 or 9 or 10 or 11 or 12 or 13 or 14 or 15 or 16  18. cohort studies/ or follow-up studies/ or longitudinal studies/ or prospective studies/ or retrospective studies/  19. Randomized Controlled Trial/  20. (cohort or longitudinal or prospective).ti,ab.  21. 18 or 19 or 20  22. 3 and 17 and 21  23. limit 22 to humans |
