# Supplementary material for: Long-term risks and benefits associated with cesarean delivery for mother, baby, and subsequent pregnancies: Systematic review and meta-analysis
Source: PLoS Med. 2018 Jan 23;15(1):e1002494. doi: 10.1371/journal.pmed.1002494 (PMC5779640; doi:10.1371/journal.pmed.1002494)
Supplement: S2 Table — (DOCX) [file pmed.1002494.s004.docx]

**S2 Table: Maternal outcomes - study characteristics**

| **Study** | **Design** | **Setting** | **Country** | **Period** | **Participants** | **Exclusions** | **Intervention** | **Outcomes** | **Follow-up** | **Risk adjustment** | **Study quality** |
| --- | --- | --- | --- | --- | --- | --- | --- | --- | --- | --- | --- |
| Abdel-Fattah (2011)  [1] | Register linkage study | Population | UK | 1950-68  linked to 2010 | 34 631 | Women over 80 years, no information on deliveries | Cesarean delivery | Pelvic organ prolapse surgery,  Urinary incontinence surgery,  Rectal prolapse/fecal incontinence surgery | Variable, up to age 80 years | Maternal age at first delivery, parity, twin delivery, time interval between deliveries, perineal wounds | + |
| Bjelland (2016)  [2] | Prospective cohort | Population | Norway | 1999-2008 | 20 248 | None | Cesarean delivery | Pelvic pain | 18 months | Maternal age, parity, educational level, birthweight, history of pain, emotional distress during pregnancy | + |
| Brown (2012)  [3] | Prospective cohort | Hospital | Australia | 2003-05 | 1507 | Multiple births | Cesarean delivery | Fecal incontinence | 12 months |  | 0 |
| Elvander (2015)  [4] | Prospective cohort | Population | Sweden | 1992-2010 | 771 690 | Multiple births, stillbirths, forceps delivery | Cesarean delivery | Interpregnancy interval,  Subsequent childbirth | 5 years | Time, maternal age, BMI, height, maternal morbidity, infertility, birthweight | + |
| Fussing-Clausen (2014) [5] | Prospective cohort | Population | Denmark | 1987-2010 | 642 052 | Parous women, multiple births, stillbirths, preterm deliveries, age <15 years | Cesarean delivery | Subsequent livebirth | >1year | Maternal age, calendar time | + |
| Gartland (2012)  [6] | Prospective cohort | Hospital | Australia | 2003-05 | 1507 | Multiple births | Cesarean delivery | Urinary incontinence | 18 months | Maternal age, infant birthweight, subsequent pregnancy, hospital delivery | + |
| Gyhagen (2013) [7] | Prospective cohort | Population | Sweden | 1985-88 | 5236 |  | CS | Symptomatic pelvic organ prolapse | 20 years | Current BMI, infant birthweight, maternal age | + |
| Gyhagen (2013) [8] | Prospective cohort | Population | Sweden | 1985-88 | 5236 |  | CS | Urinary incontinence | 21 years | BMI current and at delivery, maternal age, gestational weeks, infant birthweight, head circumference | + |
| Gyhagen (2015) [9] | Prospective cohort | Population | Sweden | 1985-88 | 5127 | >1 birth, multiparity, ongoing pregnancy | CS | Urinary incontinence  Symptomatic pelvic organ prolapse  Faecal incontinence*  Co-occurring pelvic floor disorders | 20 years | Current BMI, birthweight, maternal age | + |
| Hall (1989)  [10] | Prospective cohort | Population | UK | 1964-83 | 22 948 | Multiple births, stillbirths | Cesarean delivery | Subsequent pregnancy, Miscarriage | 1-19 years | Maternal age, height, social class | + |
| Hannah (2004)  [11] | Multi-centre RCT | Population | Multi-centre | 1997-2000 | 2088 | Evidence of fetopelvic disproportion, estimated fetal weight >4000g, hyperextension of fetal head, lethal anomaly, contraindication to labour or vaginal delivery | Planned cesarean delivery | Urinary Incontinence*,  Fecal Incontinence*, Dysmenorrhea, Menorrhagia,  Dyspareunia*,Subsequent Pregnancy | 2 years |  | ++ |
| Huttly (1990)  [12] | Prospective cohort | Hospital | Brazil | 1982 | 4978 | Previous stillbirth, sterilization | Cesarean delivery | Further pregnancy within follow-up period | Mean follow-up 43 months | Income, age, education, parity | + |
| Kjerulff (2013) [13] | Retrospective analysis of prospectively collected data | Hospital | USA | 2000 | 52 498 | Women whose infant died first 12months, or with any procedure during first childbirth that would impair fertility (sterilization, oophorectomy, hysterectomy, ablation) | Cesarean delivery | Subsequent livebirth | 8 years | Maternal age, race, education, marital status, insurance, complications in first pregnancy | + |
| Liang (2013)  [14] | Prospective cohort | Hospital | Taiwan | 2005-06 | 1501 | Severe cardiopulmonary diseases or renal diseases, pre-eclampsia, IDDM, neurogenic diseases, previous surgery for POP or UI | Cesarean delivery | Urinary stress incontinence*, urinary urge incontinence | 5 years |  | 0 |
| MacArthur (2011)  [15] | Prospective cohort | Hospital | Multi-centre: UK, NZ | 1993-94 | 3763 |  | Cesarean delivery | Urinary incontinence*  Faecal incontinence* | 12 years | Age at first birth, total number of births, ethnicity, BMI | + |
| McDonald 2015  [16] | Prospective cohort | Hospital | Australia | 2003-05 | 1507 | Para1 or more, age <18years, poor English | Cesarean delivery | Dyspareunia | 18 months | Pre-pregnancy dyspareunia, maternal age, educational level, maternal fatigue, depression, intimate partner abuse | + |
| Mollison (2005)  [17] | Retrospective analysis of prospectively collected data | Population | UK | 1980-97 | 25 471 | Multiple births, stillbirths | Cesarean delivery | First subsequent pregnancy following index delivery*, miscarriage*, ectopic pregnancy* | 4-20 years | Partner’s social class, maternal age, gestation | ++ |
| Murphy (2002) [18] | Retrospective analysis of prospectively collected data | Population | UK | 1991-92 | 14 541 |  | Cesarean delivery | Subfertility | 1 and 3 years | Maternal age, smoking, alcohol consumption, educational level, ethnicity, maternal BMI | ++ |
| O’Neill (2014)  [19] | Retrospective analysis of prospectively collected data (record linkage) | Population | Denmark | 1982-2010 | 832 996 | Parous women | Cesarean delivery | Subsequent live birth | Up to 28 years | Maternal age, country of origin, history of stillbirth, miscarriage or ectopic pregnancy, educational level, gross income, marital status, birth year, medical complications index birth, diabetes, placental complications, hypertensive complications, gestational age, birthweight, infant sex, length  Smoking, BMI, previous fertility treatment, psychiatric history | ++ |
| Rortveit (2003)  [20] | Retrospective analysis of prospectively collected data (record linkage from 1967) | Population | Norway | 1995-97 | 15 307 | Delivery before 1967, multiple pregnancies, mixed modes of delivery, age >65 years, >4 children | Cesarean delivery | Any urinary incontinence | Up to age 65 years | Age, BMI, parity, years since last delivery, birthweight, gestational age | + |
| Schytt (2004)  [21] | Prospective cohort | Population | Sweden | 1999-2000 | 2390 | Miscarriages and multiple pregnancy | Cesarean delivery | Urinary stress incontinence | 12 months | Maternal age, parity, BMI, constipation after childlbirth, incontinence after childbirth, language, marital status | 0 |
| Smith (2006)  [22] | Retrospective analysis of prospectively collected data | Population | UK | 1980-84 | 109 991 | Multiple pregnancy, perinatal deaths, births outwith 37-43 weeks gestation, missing values | Cesarean delivery | No second pregnancy*  Miscarriage* | 15 years | Marital status, deprivation category, birthweight, infant gender, maternal age, height, method of induction | ++ |
| Tollanes (2007)  [23] | Retrospective analysis of prospectively collected data | Population | Norway | a. 1967-81  b. 1982-96 | 719 544 | Women who died before age 50, mothers with new partners between pregnancies, multiple pregnancies | Cesarean delivery | Subsequent birth | Up to 21 years | Maternal level of education, maternal age.  Separate analyses for obstetric low-risk group, pre-eclampsia and breech presentation (results not altered) | + |
| Woolhouse (2012)  [24] | Prospective cohort | Hospital | Australia | 2003-05 | 1507 | Requiring interpreter | Cesarean delivery | Urinary incontinence*, Fecal incontinence*, Pelvic pain* | 18 months | Maternal age, infant birthweight | + |

S2 Table: Table showing the characteristics of included studies from maternal outcomes database search. *Where there is more than one outcome assessed, this outcome was used for meta-anlaysis. Study quality as assessed using SIGN guidelines, ++ good, + fair, 0 poor. POPQ – pelvic organ prolapse quantification examination – competent examiners used. IDDM – insulin-dependent diabetes mellitus. POP – pelvic organ prolapse. UI – urine incontinence.

**References**

1. Abdel-Fattah M, Familusi A, Fielding S, Ford J, Bhattacharya S. Primary and repeat surgical treatment for female pelvic organ prolapse and incontinence in parous women in the UK: a register linkage study. BMJ Open. 2011;1:e000206. doi: 10.1136/bmjopen-2011-00206.

2. Bjelland EK, Owe KM, Pingel R, Kristiansson P, Vangen S, Eberhard-Gran M. Pelvic pain after childbirth: a longitudinal population study. Pain. 2016;157(3):710-6. doi: 10.1097/j.pain.0000000000000427. PubMed PMID: WOS:000378258800022.

3. Brown SJ, Gartland D, Donath S, MacArthur C. Fecal Incontinence During the First 12 Months Postparum. Obstet Gynecol. 2012;119:240-9. doi: 10.1097/AOG.0b013e318242b1f7.

4. Elvander C, Dahlberg J, Andersson G, Cnattingius S. Mode of delivery and the probability of subsequent childbearing: a population-based register study. Bjog-Int J Obstet Gy. 2015;122(12):1593-600. doi: 10.1111/1471-0528.13021. PubMed PMID: WOS:000363729300032.

5. Fussing-Clausen C, Geirsson RT, Hansen T, Rasmussen S, Lidegaard O, Hedegaard M. Mode of delivery and subsequent reproductive patterns. A national follow-up study. Acta Obstetricia Et Gynecologica Scandinavica. 2014;93(10):1034-41. doi: 10.1111/aogs.12469. PubMed PMID: WOS:000342582800012.

6. Gartland D, Donath S, MacArthur C, Brown SJ. The onset, recurrence and associated obstetric risk factors for urinary incontinence in the first 18 months after a first birth: an Australian nulliparous cohort study. BJOG. 2012;119(11):1361-9. doi: 10.1111/j.1471-0528.2012.03437.x. PubMed PMID: 22827735.

7. Gyhagen M, Bullarbo M, Nielsen TF, Milsom I. Prevalence and risk factors for pelvic organ prolapse 20 years after childbirth: a national cohort study in singleton primiparae after vaginal or caesarean delivery. BJOG. 2013;120(2):152-60. doi: 10.1111/1471-0528.12020. PubMed PMID: 23121158.

8. Gyhagen M, Bullarbo M, Nielsen TF, Milsom I. The prevalence of urinary incontinence 20 years after childbirth: a national cohort study in singleton primiparae after vaginal or caesarean delivery. BJOG. 2013;120(2):144-51. doi: 10.1111/j.1471-0528.2012.03301.x. PubMed PMID: 22413831.

9. Gyhagen M, Akervall S, Milsom I. Clustering of pelvic floor disorders 20 years after one vaginal or one cesarean birth. Int Urogynecol J. 2015;26(8):1115-21. doi: 10.1007/s00192-015-2663-3. PubMed PMID: WOS:000361229500005.

10. Hall MH, Campbell D, Fraser C, Lemon J. Mode of delivery and future fertility. British Journal of Obstetrics and Gynaecology. 1989;96:1297-303.

11. Hannah ME, Whyte H, Hannah WJ, Hewson S, Amankwah K, Cheng M, et al. Maternal outcomes at 2 years after planned cesarean section versus planned vaginal birth for breech presentation at term: The international randomized Term Breech Trial. American Journal of Obstetrics and Gynecology. 2004;191(3):917-27. doi: 10.1016/j.ajog.2004.08.004.

12. Huttly SRA, Barros FC, Victora CG, Lombardi C, Vaughan JP. Subsequent pregnancies: Who has them and who wants them? Observations from an urban center in Southern Brazil. Rev Saude publ. 1990;24(3):212-6.

13. Kjerulff KH, Zhu J, Weisman CS, Ananth CV. First birth Caesarean section and subsequent fertility: a population-based study in the USA, 2000-2008. Hum Reprod. 2013;28(12):3349-57. doi: 10.1093/humrep/det343. PubMed PMID: 24021550; PubMed Central PMCID: PMC3829579.

14. Liang CC, Wu MP, Lin SJ, Lin YJ, Chang SD, Wang HH. Clinical impact of and contributing factors to urinary incontinence in women 5 years after first delivery. Int Urogynecol J. 2013;24(1):99-104. doi: 10.1007/s00192-012-1855-3. PubMed PMID: 22777581.

15. MacArthur C, Glazener C, Lancashire R, Herbison P, Wilson D. Exclusive caesarean section delivery and subsequent urinary and faecal incontinence: a 12-year longitudinal study. BJOG: An International Journal of Obstetrics & Gynaecology. 2011;118(8):1001-7. doi: 10.1111/j.1471-0528.2011.02964.x.

16. McDonald EA, Gartland D, Small R, Brown SJ. Dyspareunia and Childbirth: A Prospective Cohort Study. Obstet Gynecol Surv. 2015;70(5):319-20. PubMed PMID: WOS:000354725300013.

17. Mollison J, Porter M, Campbell D, Bhattacharya S. Primary mode of delivery and subsequent pregnancy. BJOG. 2005;112(8):1061-5. doi: 10.1111/j.1471-0528.2005.00651.x. PubMed PMID: 16045518.

18. Murphy DJ, Stirrat GM, Heron J, Team AS. The relationship between Caesarean section and subfertility in a population-based sample of 14 541 pregnancies. Hum Reprod. 2002;17(7):1914-7.

19. O'Neill SM, Khashan AS, Henriksen TB, Kenny LC, Kearney PM, Mortensen PB, et al. Does a Caesarean section increase the time to a second live birth? A register-based cohort study. Human Reproduction. 2014;29(11):2560-8. doi: 10.1093/humrep/deu217. PubMed PMID: WOS:000344675700026.

20. Rortveit G, Daltveit AK, Hannestad YS, Hunskaar S, Study NE. Urinary incontinence after vaginal delivery or cesarean section. New Engl J Med. 2003;348(10):900-7. doi: DOI 10.1056/NEJMoa021788. PubMed PMID: WOS:000181341100005.

21. Schytt E, Linkmark G, Waldenstrom U. Symptoms of stress incontinence 1 year after childbirth: prevalence and predictors in a national Swedish sample. Acta Obstet Gynecol Scand. 2004;83:928-36.

22. Smith GC, Wood AM, Pell JP, Dobbie R. First cesarean birth and subsequent fertility. Fertil Steril. 2006;85(1):90-5. Epub 2006/01/18. doi: S0015-0282(05)03433-3 [pii]

10.1016/j.fertnstert.2005.07.1289. PubMed PMID: 16412736.

23. Tollanes MC, Melve KK, Irgens LM, Skjaerven R. Reduced Fertility After Cesarean Delivery. Obstet Gynecol. 2007;110:1256-63.

24. Woolhouse H, Perlen S, Gartland D, Brown SJ. Physical Health and Recovery in the First 18 Months Postpartum: Does Cesarean Section Reduce Long-Term Morbidity? BIRTH. 2012;39(3):221-9.
