# Supplementary material for: Long-term risks and benefits associated with cesarean delivery for mother, baby, and subsequent pregnancies: Systematic review and meta-analysis
Source: PLoS Med. 2018 Jan 23;15(1):e1002494. doi: 10.1371/journal.pmed.1002494 (PMC5779640; doi:10.1371/journal.pmed.1002494)
Supplement: S3 Table — (DOCX) [file pmed.1002494.s005.docx]

**S3 Table: Childhood outcomes - study characteristics**

| **Study** | **Design** | **Setting** | **Country** | **Period** | **Participants** | **Exclusions** | **Intervention** | **Outcomes** | **Follow-up** | **Risk adjustment** | **Study quality** |
| --- | --- | --- | --- | --- | --- | --- | --- | --- | --- | --- | --- |
| Ajslev (2011)  [1] | Prospective cohort | Population | Denmark | 1997-2002 | 28 354 | Multiple pregnancies, deliveries before 37 weeks or after 43 weeks | Cesarean Delivery | Childhood overweight | 7 years | Maternal age, socioeconomic status, pre-pregnancy BMI, gestational weight gain, smoking, paternal BMI, parity, birth weight, sex breastfeeding, age at 7-year follow-up | + |
| Almqvist (2012) [2] | Prospective cohort | Population | Sweden | 1993-99 | 175 110 | Twins, pairs where one sibling deceased, or outwith medical birth register, unclear information on mode of delivery | Cesarean Delivery | Diagnosis of asthma previous 12 months | 10-13 years | Child’s gender, birth order, birth weight, gestational age, Apgar score at 5mins  Maternal age, smoking, mother living with father, mother’s birth country, maternal BMI | + |
| Andersen (2013) [3] | Prospective cohort | Population | Denmark | 1977-2009 | 1 984 758 |  | Cesarean Delivery | Inflammatory bowel disease | 0-9 years  10-19 years  20+ years | Maternal age at delivery, SGA, gestational age, birth order, hospital admissions due to infection, maternal smoking, parental history of IBD, antibiotic treatment during pregnancy | + |
| Bager (2012) [4] | Prospective cohort | Population | Denmark | 1973-2008 | 2 098 039 |  | Cesarean Delivery | Inflammatory bowel disease | 4-5 year intervals up to 35 years | Age, sex, birthweight, birth order, country of birth | + |
| Barros (2012) [5] | Prospective cohort | Population | Brazil | 3 cohorts  a. 1982  b. 1993  c. 2004 | a. 5914  b. 5249  c. 4232 |  | Cesarean Delivery | Obesity | a. 4 and 23 years  b. 4, 11 and 15years  c. 4 years | Sex, birthweight, physical activity, smoking, schooling, income, maternal schooling, smoking during  pregnancy, maternal family income, type of payment for delivery, skin colour, parity, age, pre-pregnancy weight and  height | + |
| Black (2015) [6] | Retrospective analysis of prospectively collected data | Population | UK | 1993-2007 | 321 287 | Missing data | Planned Cesarean Delivery | Asthma*  Inflammatory bowel disease*  Type 1 diabetes  Cancer  Death  Obesity (at 5 years only, 2004-07 cohort) | Up to 21 years | Maternal age, gestational age, social class, year of delivery, offspring sex, birthweight, breastfeeding, maternal salbutamol prescription, maternal diabetes, maternal BMI | ++ |
| Changzheng[7] (2016) | Prospective cohort | Population | US | 1996-2012 | 22 068 | Missing data, multiple pregnancy | Cesarean Delivery | Obesity | 20-28 years | Maternal age, race, year of birth, prepregnancy BMI, maternal height, gestational diabetes, preeclampsia, gestational age, birthweight, pregpregnancy smoking, gender, birth order | + |
| Davidson (2010) [8] | Record linkage | Population | UK | 1970-89 | 248 612 | Birthweight <1000g, missing data | Cesarean Delivery | Asthma | 2-11 years | Maternal asthma, social class, sex, birthweight, gestational age, smoking, parity | ++ |
| Eggesbo (2003) [9] | Prospective cohort | Population | Norway | 1992-93 | 2803 |  | Cesarean Delivery | Egg, nut or fish allergy | 2 years | Maternal age and education, birth weight, gestation, pre-eclampsia, high BP, pregnancy bleeding, IUGR, hyperemesis, threatened PTL, maternal smoking, breast-feeding, pregnancy complications | + |
| Eggesbo (2005) [10] | Prospective cohort | Population | Norway |  | 2656 | Children with reactions to egg, fish or nuts and non-responders | Cesarean Delivery | Cow’s milk allergy | 2 years | Maternal age, education, smoking, child’s birthweight, gestation, pregnancy complications, breast-feeding <1mth, firstborn child, maternal antibiotics during pregnancy, infant antibiotic use first 6mths | + |
| Goldani (2011)  [11] | Prospective cohort | Population | Brazil | 1978-79 | 2057 |  | Cesarean Delivery | Obesity | 23-25 years | Gender, birthweight, income, smoking, schooling, physical activity, maternal schooling, smoking during pregnancy | ++ |
| Goldani (2013)  [12] | Prospective cohort | Population | Brazil | a. 1994  b. 1997-98 | a. 2858  b. 2443 | Multiple births and stillbirths | Cesarean Delivery | Obesity | a. 10-11 years  b. 9 years | Maternal schooling, smoking, breastfeeding, newborn gender, birthweight, gestational age, type of school. Prepregnancy maternal weight (cohort b only) | + |
| Huh (2012) [13] | Prospective cohort | Hospital | USA | 1999-2002 | 1255 | Multiple pregnancy, delivery <34 weeks | Cesarean Delivery | Obesity | 3 years | Maternal age, education, race/ethnicity, child age and sex, maternal BMI, birthweight. | + |
| Kero (2002) [14] | Prospective cohort | Population | Finland | 1987 | 59 927 |  | Cesarean Delivery | Asthma | 7 years | Not specified | + |
| Li (2013) [15] | Prospective cohort | Population | China | 1993-96 | 181 380 | Unknown delivery mode, unkown birthweight or gender | Cesarean Delivery  Maternal request Cesarean Delivery | Overweight | 3-7 years | Maternal age, height, weight at first prenatal visit, BMI, weight gain during pregnancy, education, occupation, parity, folic acid supplementation, child’s gender, birth length, birthweight, gestational age, age at follow-up | + |
| Lin (2013) [16] | Prospective cohort | Population | Hong Kong | 1997 | 7809 | Premature birth, unknown gestational age | Cesarean Delivery | Obesity or  overweight (figures taken together) | Up to 13 years | Sex, gestational age, birthweight, parity, maternal BMI, maternal age, gestational diabetes, pre-eclampsia, mother’s birthplace, breastfeeding | + |
| McKeever (2002) [17] | Retrospective analysis of prospectively collected data | Population | UK |  | 24 690 |  | Cesarean Delivery | Asthma*  Wheeze  Eczema  Hay fever* | 0-11 years (median 2.9 years) | Child’s gender, prematurity, consulting behavior, parental atopy, year of birth, general practice, maternal age, parental smoking | + |
| Magnus (2011) [18] | Prospective cohort | Population | Norway | 1999-2008 | 37 171 | Multiple births | Cesarean Delivery | a. Wheeze  b. Asthma  c. LRTIs  d. Recurrent LRTIs (3 or more) | a. 18 mths  b. 36 mths  c. 18-36 mths  d. 18-36 mths | Maternal age, marital status, parity, child’s gender, maternal education level, prepregnancy BMI, smoking, preference for Cesarean Delivery, previous Cesarean Delivery, chronic conditions, pregnancy complications, duration membrane rupture, birthweight, gestational age, breastfeeding 6mths, day care 18mths, maternal atopy | + |
| Maitra (2004) [19] | Prospective cohort | Population | Bristol, UK | 1991-92 | 8299 |  | Cesarean Delivery | a. Asthma  b. Wheeze*  c. physician-diagnosed asthma*  d. atopy* | a. 5-7 years  b. 5-7 years  c. 91 mths  d. 91 mths | Maternal education, smoking, maternal hayfever, ethnicity, number of other children in household, financial difficulties, damp housing, child’s contact with cats, duration breastfeeding, smoke exposure, birthweight, sex, gestational age, maternal age at delivery | 0 |
| Mamun (2013)  [20] | Prospective cohort | Hospital | Australia | 1981-83 | 2625 |  | Cesarean Delivery | Obesity | 5, 14 and 21 years | Maternal age at birth, offspring sex, race, maternal education, smoking, gestation, birthweight, prepregnancy BMI, gestational weight gain, hypertensive disorder | + |
| Menezes (2010) [21] | Prospective | Population | Brazil | 2 cohorts  a. 1993  b. 2004 | a. 5249  b. 4288 |  | Cesarean Delivery | Wheeze | a. 4, 11 and 15 years  b. 4 years | Child’s gender, birthweight, gestational age, IUGR, skin colour, birth order, maternal age, education, smoking wheeze, paternal smoking, wheeze, socio-economic position | + |
| Mesquita (2013)  [22] | Prospective cohort | Population | Brazil | 1978-79 | 2063 | Those who did not reside in the city at time of delivery | Cesarean Delivery | Anthropometric measurements: height, WC*, waist-height ratio, waist-hip ratio, tricipital and subscapular skinfolds | 23-25 years | Birthweight, type of delivery, sex, maternal schooling, maternal smoking, parity, maternal age, gestational age | + |
| Negele (2004) [23] | Prospective cohort | Population | Germany | 1997-99 | 2500 | Described elsewhere, first twin used only | Cesarean Delivery | Physician-diagnosed asthma*  Atopy  Atopic dermatitis  Atopic rhinoconjunctivitis  Wheeze*  Specific IgE* (range of food and inhalant allergens) | 2 years | Gender, study area, parental atopy, maternal smoking, birthweight, maternal education, breastfeedin  g, other  household children | + |
| Pei (2014)  [24] | Prospective cohort | Population | Germany | 1997-99 | 1734 | Congenital disorders, perinatal problems. Gestational age <37weeks and birthweight <2500g. | Cesarean Delivery | Overweight  Obesity | 2*, 6 and 10* years | Parental education, city of recruitment, birthweight, duration gestation, head circumference, maternal age, prepregnancy BMI, smoking during pregnancy, early feeding variables | + |
| Ponsonby (2009) [25] | Record linkage | Population | Australia | 1983-98 | 998 599 |  | Cesarean Delivery | Crohn’s disease | Up to 16 years | Sex, congenital abnormality, urban area at birth, married mother, socioeconomic status, maternal age, year of birth | ++ |
| Pyrhonen (2013) [26] | Prospective cohort  Record linkage | Population | Finland | 2001-06 | 3181 |  | Cesarean Delivery | a. asthma*, allergic conjunctivitis, urticaria, eczema, hay fever*, allergy for good/animal/pollen  b. allergy to food/animal/pollen/any allergen* | 1-4 years | Gender, birth order, gestation, birthweight, parental allergy, duration breastfeeding, smoking during pregnancy, child’s age at questionnaire | + |
| Roberts (2011) [27] | Record linkage | Population | UK | 1970-89 | 248 659 | Abortions, stillbirths, early deaths within 30 days of birth, birthweight <1000g | Cesarean Delivery | Inflammatory bowel disease | Up to 29 years | Year of birth, sex, number of babies, gestational age, birthweight, head circumference, breastfeeding, Apgar scores | ++ |
| Roduit (2009) [28] | Prospective cohort | Population | Holland | 1996-97 | 2917 | Twins, missing data for mode of delivery, parental history of allergy | Cesarean Delivery | a. Asthma*, wheeze*  b. Any specific IgE positive (to food or inhalant)* | 8 years | Gender, birthweight, maternal education, breastfeeding, maternal BMI, parental allergy, IUGR, Apgar score, breathing problems after delivery | + |
| Steur (2011)  [29] | Prospective cohort | Population | Holland | 1996-97 | 1687 |  | Cesarean Delivery | Overweight | 8 years |  | 0 |
| Tollanes (2008) [30] | Prospective cohort | Population | Norway | 1967-98 | 1 756 700 | Children who died <1yr, birth defects, multiple pregnancy | Cesarean Delivery | Asthma | Up to 18 years or until 2002 | Maternal age, birth order, maternal asthma, education, year of birth, child’s sex, gestational age | ++ |
| Van Berkel (2015) [31] | Prospective cohort | Population | Holland | 2002-2006 | 6128 | Missing data on mode of delivery, wheezing patterns, asthma | Cesarean Delivery | a. early wheeze*  b. persistent wheeze*  c. asthma* | a. up to 3 years  b. up to 6 years  c. 6 years | Maternal age, pre-pregnancy BMI, educational level, parity, history of asthma or atopy, pet keeping, maternal smoking, gestational diabetes, pre-eclampsia, pregnancy-induced hypertension, child’s sex, ethnicity, gestational age, birthweight | 0 |
| Van Nimwegen (2011) van [32] | Prospective cohort | Population | Holland | 2002 | 1926 | Congential abnormalities, birth <37weeks | Cesarean Delivery | a. Asthma*  b. Wheeze  c. Eczema  d.Specific IgE levels | 7 years | Recruitment (conventional/alternative lifestyle), sex, birthweight, breastfeeding, maternal smoking, maternal age, education level, siblings atopy, parental atopy | + |
| Werner (2006) [33] | Prospective cohort | Population | Denmark | 1984-87 | 7119 | Birthweight <2500g, gestational age <37 weeks, Apgars <7 at 5mins | Cesarean Delivery | Asthma | 15-18 years | Breastfeeding, maternal smoking, previous deliveries, child’s sex | 0 |
| Xu (2000) [34] | Prospective cohort | Population | Finland | 1985-86 | 8088 |  | Cesarean Delivery | Asthma | 7 years | Gender, birthweight, gestational age, maternal allergic disorder | + |
| Xu (2001) [35] | Prospective cohort | Population | Finland | 1966 | 6025 |  | Cesarean Delivery | Asthma*  Hay fever*  Eczema  Allergy | 31 years | Maternal age, maternal BMI, age at menarche, parity, smoking, paternal social class, birthweight, current BMI, physical activities, vocational training, gestational age, sex, parental allergy | + |

S3 Table: Table showing the characteristics of included studies from childhood outcomes database search. *Where more than one outcome assessed, this was used in meta-analysis. BAZ – BMI-for-age Z score. IUGR – intrauterine growth restriction. PTL – preterm labour. SGA – small for gestational age. WC – waist circumference

**References**

1. Ajslev TA, Andersen CS, Gamborg M, Sorensen TI, Jess T. Childhood overweight after establishment of the gut microbiota: the role of delivery mode, pre-pregnancy weight and early administration of antibiotics. Int J Obes (Lond). 2011;35(4):522-9. doi: 10.1038/ijo.2011.27. PubMed PMID: 21386800.
2. Almqvist C, Cnattingius S, Lichtenstein P, Lundholm C. The impact of birth mode of delivery on childhood asthma and allergic diseases--a sibling study. Clin Exp Allergy. 2012;42(9):1369-76. doi: 10.1111/j.1365-2222.2012.04021.x. PubMed PMID: 22925323; PubMed Central PMCID: PMC3564396.
3. Andersen V, Erichsen R, Froslev T, Sorensen HT, Ehrenstein V. Differential risk of ulcerative colitis and Crohn's disease among boys and girls after cesarean delivery. Inflamm Bowel Dis. 2013;19(1):E8-E10. doi: 10.1002/ibd.22841. PubMed PMID: 22147542.
4. Bager P, Simonsen J, Nielsen NM, Frisch M. Cesarean section and offspringʼs risk of inflammatory bowel disease: A national cohort study. Inflammatory Bowel Diseases. 2012;18(5):857-62. doi: 10.1002/ibd.21805.
5. Barros FC, Matijasevich A, Hallal PC, Horta BL, Barros AJ, Menezes AB, et al. Cesarean section and risk of obesity in childhood, adolescence, and early adulthood: evidence from 3 Brazilian birth cohorts. Am J Clin Nutr. 2012;95(2):465-70. doi: 10.3945/ajcn.111.026401. PubMed PMID: 22237058; PubMed Central PMCID: PMC3260073.
6. Black M, Bhattacharya S, Philip S, Norman JE, McLernon DJ. Planned Cesarean Delivery at Term and Adverse Outcomes in Childhood Health. Jama-J Am Med Assoc. 2015;314(21):2271-9. doi: 10.1001/jama.2015.16176. PubMed PMID: WOS:000365515700019.
7. Changzheng Y, Gaskins AJ, Blaine AI, Zhang C, Gillman MW, Missmer SA, et al. Association Between Cesarean Birth and Risk of Obesity in Offspring in Childhood, Adolescence, and Early Adulthood. JAMA Pediatrics. 2016;170(11):e162385.
8. Davidson R, Roberts SE, Wotton CJ, Goldacre MJ. Influence of maternal and perinatal factors on subsequent hospitalisation for asthma in children: evidence from the Oxford record linkage study. Bmc Pulm Med. 2010;10. doi: Artn 14

10.1186/1471-2466-10-14. PubMed PMID: WOS:000208592700014.

1. Eggesbø M, Botten G, Stigum H, Nafstad P, Magnus P. Is delivery by cesarean section a risk factor for food allergy? Journal of Allergy and Clinical Immunology. 2003;112(2):420-6. doi: 10.1067/mai.2003.1610.
2. Eggesbo M, Botten G, Stigum H, Samuelsen SO, Brunekreef B, Magnus P. Cesarean delivery and cow milk allergy/intolerance. Allergy. 2005;60(9):1172-3. doi: 10.1111/j.1398-9995.2005.00857.x. PubMed PMID: 16076303.
3. Goldani HA, Bettiol H, Barbieri MA, Silva AA, Agranonik M, Morais MB, et al. Cesarean delivery is associated with an increased risk of obesity in adulthood in a Brazilian birth cohort study. Am J Clin Nutr. 2011;93(6):1344-7. doi: 10.3945/ajcn.110.010033. PubMed PMID: 21508088.
4. Goldani MZ, Barbieri MA, Moura da Silva AA, Pereria Gutierrez MR, Bettiol H, Goldani HA. Cesarean section and increased body mass index in school children: two cohort studies from distinct socioeconomic background areas in Brazil. Nutrition Journal. 2013;12.
5. Huh SY, Rifas-Shiman SL, Zera CA, Edwards JW, Oken E, Weiss ST, et al. Delivery by caesarean section and risk of obesity in preschool age children: a prospective cohort study. Archives of disease in childhood. 2012;97(7):610-6. Epub 2012/05/25. doi: 10.1136/archdischild-2011-301141. PubMed PMID: 22623615; PubMed Central PMCID: PMC3784307.
6. Kero J, Gissler M, Minna-Maija G, Kero P, Koskinen P, Hemminki E, et al. Mode of Delivery and Asthma - Is There a Connection? Pediatr Res. 2002;52(1):6-11. doi: 10.1023/01.PDR.0000017262.01840.F0.
7. Li HT, Ye R, Pei L, Ren A, Zheng X, Liu JM. Caesarean delivery, caesarean delivery on maternal request and childhood overweight: a Chinese birth cohort study of 181 380 children. Pediatr Obes. 2014;9(1). Epub 2013 Mar 19.
8. Lin SL, Schooling CM, Leung GM. Mode of Delivery and Adiposity: Hong Kong's "Children of 1997" Birth Cohort. American Journal of Epidemiology. 2013;177:S8-S. PubMed PMID: WOS:000319870300031.
9. McKeever TM, Lewis SA, Smith C, Hubbard R. Mode of delivery and risk of developing allergic disease. Journal of Allergy and Clinical Immunology. 2002;109(5):800-2. doi: 10.1067/mai.2002.124046.
10. Magnus MC, Haberg SE, Stigum H, Nafstad P, London SJ, Vangen S, et al. Delivery by Cesarean section and early childhood respiratory symptoms and disorders: the Norwegian mother and child cohort study. Am J Epidemiol. 2011;174(11):1275-85. Epub 2011/11/01. doi: 10.1093/aje/kwr242. PubMed PMID: 22038100; PubMed Central PMCID: PMC3254156.
11. Maitra A, Sherriff A, Strachan D, Team AS, Henderson J. Mode of delivery is not associated with asthma or atopy in childhood. Clin Exp Allergy. 2004;34:1349-55.
12. Mamun AA, Sutharsan R, O'Callaghan M, Williams G, Najman J, McIntyre HD, et al. Cesarean delivery and the long-term risk of offspring obesity. Obstet Gynecol. 2013;122(6):1176-83. doi: 10.1097/AOG.0000000000000016. PubMed PMID: 24201680.
13. Menezes AM, Hallal PC, Matijasevich AM, Barros AJ, Horta BL, Araujo CL, et al. Caesarean sections and risk of wheezing in childhood and adolescence: data from two birth cohort studies in Brazil. Clin Exp Allergy. 2011;41(2):218-23. doi: 10.1111/j.1365-2222.2010.03611.x. PubMed PMID: 20840395; PubMed Central PMCID: PMC3505367.
14. Schooling CM, Mesquita DN, Barbieri MA, Goldani HAS, Cardoso VC, Goldani MZ, et al. Cesarean Section Is Associated with Increased Peripheral and Central Adiposity in Young Adulthood: Cohort Study. PLoS ONE. 2013;8(6):e66827. doi: 10.1371/journal.pone.0066827.
15. Negele K, Heinrick J, Borte M, von Berg A, Schaaf B, Lehmann I, et al. Mode of delivery and development of atopic disease during the first 2 years of life. Pediatric Allergy and Immunology. 2004;15:48-54.
16. Pei Z, Heinrich J, Fuertes E, Flexeder C, Hoffmann B, Lehmann I, et al. Cesarean delivery and risk of childhood obesity. J Pediatr. 2014;164(5):1068-73 e2. doi: 10.1016/j.jpeds.2013.12.044. PubMed PMID: 24508442.
17. Ponsonby AL, Catto-Smith AG, Pezic A, Dupuis S, Halliday J, Cameron D, et al. Association between early-life factors and risk of child-onset Crohn's disease among Victorian children born 1983-1998: a birth cohort study. Inflamm Bowel Dis. 2009;15(6):858-66. doi: 10.1002/ibd.20842. PubMed PMID: 19107784.
18. Pyrhonen K, Nayha S, Hiltunen L, Laara E. Caesarean section and allergic manifestations: insufficient evidence of association found in population-based study of children aged 1 to 4 years. Acta Paediatr. 2013;102(10):982-9. doi: 10.1111/apa.12342. PubMed PMID: 23826787.
19. Roberts SE, Wotton CJ, Williams JG, Griffith M, Goldacre MJ. Perinatal and early life risk factors for inflammatory bowel disease. World J Gastroenterol. 2011;17(6):743-9. doi: 10.3748/wjg.v17.i6.743. PubMed PMID: 21390144; PubMed Central PMCID: PMC3042652.
20. Roduit C, Scholtens S, de Jongste JC, Wijga AH, Gerritsen J, Postma DS, et al. Ashtma at 8 years of age in children born by caesarean section. Thorax. 2009;64:107-13. doi: 10.1136/thx.2008.100875.
21. Steur M, Smit HA, Schipper CMA, Scholtens S, Kerkhof M, de Jongste JC, et al. Predicting the risk of newborn children to become overweight later in childhood: the PIAMA birth cohort study. Int J Pediatr Obes. 2011;6:e170-e8.
22. Tollanes MC, Moster D, Daltveit AK, Irgens LM. Cesarean section and risk of severe childhood asthma: a population-based cohort study. J Pediatr. 2008;153(1):112-6. doi: 10.1016/j.jpeds.2008.01.029. PubMed PMID: 18571547.
23. van Berkel AC, den Dekker HT, Jaddoe VWV, Reiss IK, Gaillard R, Hofman A, et al. Mode of delivery and childhood fractional exhaled nitric oxide, interrupter resistance and asthma: the Generation R study. Pediatric Allergy and Immunology. 2015;26(4):330-6. doi: 10.1111/pai.12385. PubMed PMID: WOS:000355149800005.
24. van Nimwegen FA, Penders J, Stobberingh EE, Postma DS, Koppelman GH, Kerkhof M, et al. Mode and place of delivery, gastrointestinal microbiota, and their influence on asthma and atopy. J Allergy Clin Immunol. 2011;128(5):948-55 e1-3. doi: 10.1016/j.jaci.2011.07.027. PubMed PMID: 21872915.
25. Werner A, Ramlau-Hansen CH, Jeppesen SK, Thulstrup AM, Olsen J. Caesarean delivery and risk of developing asthma in the offspring. Acta Paediatr. 2007;96(4):595-6. doi: 10.1111/j.1651-2227.2006.00150.x. PubMed PMID: 17274805.
26. Xu B, Pekkanen J, Jarvelin MR. Obstetric Complications and Asthma in Childhood. Journal of Asthma. 2000;37(7):589-94.
27. Xu B, Pekkanen J, Hartikainen AL, Jarvelin MR. Caesarean section and risk of asthma and allergy in adulthood. J Allergy Clin Immunol. 2001;107(4):732-3. doi: 10.1067/mai.2001.113048. PubMed PMID: 11295666.
