# Supplementary material for: Long-term risks and benefits associated with cesarean delivery for mother, baby, and subsequent pregnancies: Systematic review and meta-analysis
Source: PLoS Med. 2018 Jan 23;15(1):e1002494. doi: 10.1371/journal.pmed.1002494 (PMC5779640; doi:10.1371/journal.pmed.1002494)
Supplement: S4 Table — (DOCX) [file pmed.1002494.s006.docx]

**S4 Table: Subsequent pregnancy outcomes - study characteristics**

| **Study** | **Design** | **Setting** | **Country** | **Period** | **Participants** | **Exclusions** | **Intervention** | **Outcomes** | **Follow-up** | **Risk adjustment** | **Study quality** |
| --- | --- | --- | --- | --- | --- | --- | --- | --- | --- | --- | --- |
| Bowman (2015) [1] | Retrospective analysis of prospectively collected data | Population | USA | 1996-2011 | 255 082 | Incomplete data | Previous Cesarean Delivery | Ectopic pregnancy |  | Maternal age, ethnicity, marital status, education level, prior ectopic pregnancy | + |
| Daltveit (2008)  [2] | Retrospective analysis of prospectively collected data | Population | Norway | 1967-2003 | 637 497 | Birthweight <500g, delivery <20weeks gestation | Previous Cesarean Delivery | Placenta previa*  Placenta accreta*  Placental abruption*  Uterine rupture*  Pre-eclampsia  SGA*  Bleeding during pregnancy |  | Adverse outcomes in previous pregnancy, maternal age, year of birth | + |
| Downes (2015) [3] | Retrospective analysis of prospectively collected data | Population | USA | 2002-10 | 26 987 | Parous women, multiple births | Previous Cesarean Delivery | Placenta previa |  | Maternal age, pregnancy loss, history of previa, insurance, smoking, anemia, pregestational diabetes mellitus, thyroid disease | ++ |
| Galyean (2009)  [4] | Retrospective analysis of prospectively collected data | Population | USA | 2002-03 | 10 654 |  | Previous Cesarean Delivery | Maternal outcomes: blood transfusion, admission to ICU, serious postpartum infections requiring aminogylcoside administration, VTE, hospital readmission. Neonatal outcomes: infection, seizures, encephalopathy, surfactant use, ventilatory support, prolonged hospitalisation, death* |  | Hypertension, diabetes | + |
| Getahun (2006) [5] | Retrospective analysis of prospectively collected data | Population | USA | 1989-97 | 157 831 | Multiple births, delivery <20weeks, birthweight <500g, births missing mode of delivery | Previous Cesarean Delivery | Placenta previa*  Placental abruption* |  | Maternal age, race, education, prentatal care, marital status, interpregnancy interval, smoking, alcohol during pregnancy | ++ |
| Gray (2007)[6] | Retrospective analysis of prospectively collected data | Population | UK | 1968-89 | 81 707 | Delivery <28 weeks or >43 weeks, perinatal death due to congenital anomaly or rhesus isoimmunisation, implausible interpregnancy interval, deliveries with missing data | Previous Cesarean Delivery | Stillbirth: explained and unexplained |  | Socioeconomic status, prepregnancy weight, maternal age, parity, smoking, previous adverse pregnancy outcome | ++ |
| Gurol-Urganci (2011) [7] | Retrospective analysis of prospectively collected data | Hospital | UK | 2000-09 | 399 674 |  | Previous Cesarean Delivery | Placenta previa |  | Maternal age, ethnicity, deprivation, inter-birth interval, placenta previa at first birth, pregnancy complications | ++ |
| Hall (1989)  [8] | Prospective cohort | Population | UK | 1964-83 | 22 948 | Multiple births, stillbirths | Cesarean delivery | Subsequent pregnancy*  Subsequent birth  Miscarriage* | 1-19 years | Maternal age, height, social class | + |
| Hemminki (2005) [9] | Retrospective analysis of prospectively collected data | Population | Finland | 1989-98 | 72 200 | Incomplete data | Previous Cesarean Delivery | Maternal: Fetal malposition during pregnancy, hospitalization during pregnancy, prelabor hemorrhage, placental problems at birth, asphyxia at birth, fetal malposition at birth, induction of labor, and mode of delivery. Neonatal: mean length of gestation, preterm birth (gestational age <37 weeks), mean birth weight, low birth weight (<2500g), perinatal death from 22 weeks of gestation until 1 week of age, mean Apgar score at 1 min, low Apgar-score (0-6) at 1 min, resuscitation with intubation, special care in surveillance unit, intensive care unit, or other hospital |  | Age, smoking, and infant sex at second birth. Adjustment which excluded women with 'persistent problems' in first birth: diabetes mellitus, chronic renal disease, cardiac disease, hypertension, perinatal death, preterm birth (<37 weeks of gestation), child’s birth weight <2500g, cerclage, or placental problems | + |
| Huang (2011) [10] | Retrospective analysis of prospectively collected data | Population | USA | 1995-2002 | 9 643 175 | Unknown mode of first delivery, births <24 weeks, birthweight <500g, women with chronic health problems or adverse birth history | Previous Cesarean Delivery | Preterm delivery <37 weeks, large-for-gestational age, small-for-gestational age, Apgar <7 at 5mins, assisted ventilation, fetal distress, seizure, neonatal death, asphyxia-related neonatal death* |  | Maternal age, marital status, years of schooling, race, smoking, prenatal care in first trimester, infant sex, preterm birth | + |
| Jackson (2012) [11] | Retrospective analysis of prospectively collected data | Population | Denmark | 1994-2010 | 24 839 | Incorrect coding of mode of delivery, first pregnancy ending in miscarriage | Previous Cesarean Delivery | Placenta previa*, placenta accrete*, placental abruption*, uterine rupture*, hysterectomy, anemia, PPH, miscarriage*, ectopic*, stillbirth*, preterm birth* |  | Maternal age, BMI, alcohol use, socioeconomic status | + |
| Kennare (2007) [12] | Retrospective analysis of prospectively collected data | Population | Australia | 1998-2003 | 36 038 |  | Previous Cesarean Delivery | placenta previa*, placental abruption*, antepartum hemorrhage, malpresentation, prolonged labour, Cesarean Delivery, emergency Cesarean Delivery, uterine rupture, placenta accreta, maternal death  Perinatal: preterm birth <37 weeks, very preterm birth <32 weeks, SGA, birth weight <2500g, stillbirth*, unexplained stillbirth, neonatal death* |  | age, indigenous status, smoking, pregnancy interval, medical complications such as hypertension/diabetes/asthma, obstetric complications, hospital category, patient type (public/private), gestation, history of ectopic/miscarriage/stillbirth/termination | + |
| Lydon-Rochelle (2001) [13] | Retrospective analysis of prospectively collected data | Population | USA | 1987-96 | 95 630 | First-birth placental abruption or placenta previa | Previous Cesarean Delivery | Placental abruption*  Placenta previa* |  | Maternal age, medical and pregnancy complications | + |
| Mollison (2005)  [14] | Retrospective analysis of prospectively collected data | Population | UK | 1980-97 | 25 471 | Multiple births, stillbirths | Cesarean delivery | First subsequent pregnancy following index delivery, miscarriage, ectopic pregnancy | 4-20 years | Age, smoking, and infant sex at second birth. Adjustment which excluded women with 'persistent problems' in first birth: diabetes mellitus, chronic renal disease, cardiac disease, hypertension, perinatal death, preterm birth (<37 weeks of gestation), child’s birth weight <2500g, cerclage, or placental problems | ++ |
| Moraitis (2015) [15] | Record linkage | Population | UK | 1999-2008 | 128 585 | Multiple pregnancy, perinatal death secondary to congenital abnormality or rhesus isoimmunisation, delivery outside 24-43 weeks,  birthweight <500g | Cesarean Delivery | Antepartum stillbirth |  | Maternal age, height, smoking status, socio-economic deprivation | + |
| Osborne (2012) [16] | Retrospective analysis of prospectively collected data | Population | USA | 1994-2002 (4x waves of data collection) | 11 581 | Multiple pregnancy, congenital anomalies, gestational age <24 or >43 weeks in index pregnancy | Previous Cesarean Delivery | Antepartum stillbirth |  |  | + |
| Rasmussen (2000) [17] | Retrospective analysis of prospectively collected data | Population | Norway | 1967-92 | 370 374 | Women with only one delivery, or first delivery before 1967, multiple births | Previous Cesarean Delivery | Placenta previa |  | Maternal age, prior abruption, gestation first pregnancy, prior previa, prior perinatal death | ++ |
| Salihu (2006) [18] | Retrospective analysis of prospectively collected data | Population | USA | 1978-97 | 396 441 | Congenital anomalies | Previous Cesarean Delivery | Stillbirth*  Secondary outcomes: anemia, type 1 diabetes, cardiac disease, chronic hypertension, pre-eclampsia, placental abruption*, placenta previa* |  | Maternal age, parity, marital status, educational status, smoking, BMI, adequacy of prenatal care, SGA/preterm first pregnancy, interpregnancy interval, year of birth | + |
| Salihu (2011) [19] | Retrospective analysis of prospectively collected data | Population | USA | 1978-2005 | 450 151 |  | Previous Cesarean Delivery | Neonatal death* (early and late), SGA, LGA, low birthweight, high birthweight, very preterm, moderately preterm |  | Infant sex, maternal age, race, BMI, educational level, marital status, smoking, alcohol use, interpregnancy interval, adequacy of prenatal care, history of SGA or LGA | + |
| Smith (2003) [20] | Retrospective analysis of prospectively collected data | Population | UK | 1980-98 | 103 790 | Multiple pregnancy, delivery <24 weeks or >43 weeks, birthweight <500g, perinatal deaths due to congenital anomaly or rhesus isoimmunisation, missing values | Previous Cesarean Delivery | Stillbirths: antepartum and intrapartum, explained and unexplained  (all causes antepartum stillbirth used in meta-analysis) |  | Socioeconomic deprivation, smoking, maternal age, maternal height | + |
| Smith (2006)  [21] | Retrospective analysis of prospectively collected data | Population | UK | 1980-84 | 109 991 | Multiple pregnancy, perinatal deaths, births outwith 37-43 weeks gestation, missing values | Cesarean delivery | No second pregnancy  Miscarriage | 15 years | Marital status, deprivation category, birthweight, infant gender, maternal age, height, method of induction | ++ |
| Taylor (2005) [22] | Retrospective analysis of prospectively collected data | Population | Australia | 1994-2002 | 136 101 | Multiple pregnancies, brith <24 weeks or >44 weeks, missing values |  | Maternal: stillbirth*, uterine rupture*, hysterectomy*, PPH, postpartum infection, admission to ICU, manual removal of placenta  Neonatal: neonatal death*, infant death, admission to NICU, SGA, RDS, bacterial sepsis, preterm delivery |  | Maternal age, prior uterine curettage, smoking in pregnancy, health insurance status, ethnicity, socio-economic group, pre-existing diabetes, gestational diabetes, pre-existing hypertension, PIH, labour, non-vertex presentation, gestational age, prelabor premature rupture of membranes, prior stillbirth, fetal sex, gestational age, SGA | ++ |
| Wood (2008) [23] | Retrospective analysis of prospectively collected data | Population | Canada | 1991-2004 | 158 502 | Variables missing | Previous Cesarean Delivery | Stillbirth (>24 weeks) :total and unexplained |  | Maternal age, diabetes, hypertension, smoking, weight>91kg | ++ |
| Yang (2007) [24] | Retrospective analysis of prospectively collected data | Population | USA | 1995-2000 | 5 146 742 | Missing information | Previous Cesarean Delivery | Placenta previa*  Placental abruption* |  | Maternal age, race, education, marital status, alcohol and smoking during pregnancy, adequacy of prentatal care, fetal gender | + |

S4 Table: Table showing the characteristics of included studies from fertility and subsequent pregnancy outcomes database search. *Where more than one outcome was assessed, this outcome was included in a meta-analysis. PPH – postpartum hemorrhage. SGA – small for gestational age. RDS – respiratory distress syndrome. ICU – intensive care unit, NICU – neonatal intensive care unit

**References**

1. Bowman ZS SK, Silver RM. Cesarean Delivery and Risk for Subsequent Ectopic Pregnancy. American journal of perinatology 2015;32(9):815-20.
2. Daltveit AK, Tollanes MC, Pihlstrom H, Irgens LM. Cesarean Delivery and Subsequent Pregnancies. Obstet Gynecol. 2008;111:1327-34.
3. Downes KL HS, Sjaarda LA, et al. Previous prelabor or intrapartum cesarean delivery and risk of placenta previa. Am J Obstet Gynecol 2015;212(5):669 e1-6.
4. Galyean AM, Lagrew DC, Bush MC, Kurtzman JT. Previous cesarean section and the risk of postpartum maternal complications and adverse neonatal outcomes in future pregnancies. Journal of Perinatology. 2009;29:726-30.
5. Getahun D, Oyelese Y, Salihu HM, Ananth CV. Previous cesarean delivery and risks of placenta previa and placental abruption. Obstet Gynecol. 2006;107(4):771-8.
6. Gray R, Quigley MA, Hockley C, Kurinczuk JJ, Goldacre M, Brocklehurst P. Caesarean delivery and risk of stillbirth in subsequent pregnancy: a retrospective cohort study in an English population. BJOG. 2007;114(3):264-70. doi: 10.1111/j.1471-0528.2006.01249.x. PubMed PMID: 17261119.
7. Gurol-Urganci I, Cromwell DA, Edozien LC, Smith GC, Onwere C, Mahmood TA, et al. Risk of placenta previa in second birth after first birth cesarean section: a population-based study and meta-analysis. BMC Pregnancy Childbirth. 2011;11:95. doi: 10.1186/1471-2393-11-95. PubMed PMID: 22103697; PubMed Central PMCID: PMC3247856.
8. Hall MH, Campbell D, Fraser C, Lemon J. Mode of delivery and future fertility. British Journal of Obstetrics and Gynaecology. 1989;96:1297-303.
9. Hemminki E, Shelley J, Gissler M. Mode of delivery and problems in subsequent births: a register-based study from Finland. Am J Obstet Gynecol. 2005;193(1):169-77. doi: 10.1016/j.ajog.2004.11.007. PubMed PMID: 16021075.
10. Huang X, Lei J, Tan H, Walker M, Zhou J, Wen SW. Cesarean delivery for first pregnancy and neonatal morbidity and mortality in second pregnancy. European Journal of Obstetrics & Gynaecology and Reproductive Biology. 2011;158:204-8.
11. Jackson S, Fleege L, Fridman M, Gregory K, Zelop C, Olsen J. Morbidity following primary cesarean delivery in the Danish National Birth Cohort. Am J Obstet Gynecol. 2012;206(2):139 e1-5. doi: 10.1016/j.ajog.2011.09.023. PubMed PMID: 22051815.
12. Kennare R, Tucker G, Heard A, Chan A. Risks of Adverse Outcomes in the Next Birth After a First Cesarean Delivery. Obstet Gynecol. 2007;109:270-6.
13. Lydon-Rochelle M, Holt VL, Easterling TR, Martin DP. First-Birth Cesarean and Placental Abruption or Previa at Second Birth. Obstet Gynecol. 2001;97:765-9.
14. Mollison J, Porter M, Campbell D, Bhattacharya S. Primary mode of delivery and subsequent pregnancy. BJOG. 2005;112(8):1061-5. doi: 10.1111/j.1471-0528.2005.00651.x. PubMed PMID: 16045518.
15. Moraitis AA, Oliver-Williams C, Wood AM, Fleming M, Pell JP, Smith GCS. Previous caesarean delivery and the risk of unexplained stillbirth: retrospective cohort study and meta-analysis. Bjog-Int J Obstet Gy. 2015;122(11):1467-74. doi: 10.1111/1471-0528.13461. PubMed PMID: WOS:000362752100009.
16. Osborne C, Ecker JL, Gauvreau K, Lieberman E. First birth cesarean and risk of antepartum fetal death in a subsequent pregnancy. J Midwifery Womens Health. 2012;57(1):12-7. doi: 10.1111/j.1542-2011.2011.00142.x. PubMed PMID: 22251907.
17. Rasmussen S, Albrechtsen S, Dalaker K. Obstetric history and the risk of placenta previa. Acta Obstet Gynecol Scand. 2000;79:502-7.
18. Salihu HM, Sharma PP, Kristensen S, Blot C, Alio AP, Ananth CV, et al. Risk of Stillbirth Following a Cesarean Delivery. Obstet Gynecol. 2006;107:383-90.
19. Salihu HM, Bowen CM, Wilson RE, Marty PJ. The impact of previous cesarean section on the success of future fetal programming pattern. Arch Gynecol Obstet. 2011;284(2):319-26. doi: 10.1007/s00404-010-1665-0. PubMed PMID: 20821225.
20. Smith GC, Pell JP, Dobbie R. Caesarean section and risk of unexplained stillbirth in subsequent pregnancy. Lancet. 2003;362(9398):1779-84. Epub 2003/12/05. doi: S0140673603148969 [pii]. PubMed PMID: 14654315.
21. Smith GC, Wood AM, Pell JP, Dobbie R. First cesarean birth and subsequent fertility. Fertil Steril. 2006;85(1):90-5. Epub 2006/01/18. doi: S0015-0282(05)03433-3 [pii]10.1016/j.fertnstert.2005.07.1289. PubMed PMID: 16412736.
22. Taylor LK, Simpson JM, Roberts CL, Olive EC, Henderson-Smart DJ. Risk of complications in a second pregnancy following caesarean section in the first pregnancy: a population-based study. MJA. 2005;183:515-9.
23. Wood SL, Chen S, Ross S, Sauve R. The risk of unexplained antepartum stillbirth in second pregnancies following caesarean section in the first pregnancy. BJOG: An International Journal of Obstetrics and Gynaecology. 2008;115(6):726-31. doi: 10.1111/j.1471-0528.2008.01705.x.
24. Yang Q, Wen SW, Oppenheimer L, Chen XK, Black D, Gao J, et al. Association of caesarean delivery for first birth with placenta praevia and placental abruption in second pregnancy. BJOG. 2007;114(5):609-13. doi: 10.1111/j.1471-0528.2007.01295.x. PubMed PMID: 17355267.
