# Supplementary material for: Long-term risks and benefits associated with cesarean delivery for mother, baby, and subsequent pregnancies: Systematic review and meta-analysis
Source: PLoS Med. 2018 Jan 23;15(1):e1002494. doi: 10.1371/journal.pmed.1002494 (PMC5779640; doi:10.1371/journal.pmed.1002494)
Supplement: S5 Table — (DOCX) [file pmed.1002494.s007.docx]

**S5 Table: The Risk of Bias Assessment tool for Non-randomized Studies (RoBANS)**

Maternal outcomes studies

| Study | Selection of participants | Confounding variables | Measurement of exposure | Blinding of outcome assessments | Incomplete outcome data | Selective outcome reporting |
| --- | --- | --- | --- | --- | --- | --- |
| Abdel-Fattah (2011)[1] | Low | Low | Low | Unclear | Unclear | Low |
| Bjelland (2016)  [2] | Low | Low | High | Unclear | Low | Low |
| Brown (2012)  [3] | Low | High | High | High | High | Low |
| Elvander (2015)  [4] | Low | Low | Low | Unclear | Unclear | Low |
| Fussing-Clausen (2014) [5] | Low | High | Low | Unclear | Unclear | Low |
| Gartland (2012)  [6] | Low | Low | High | High | Unclear | Low |
| Gyhagen (2013) [7] | Low | Low | High | High | Low | Low |
| Gyhagen (2013) [8] | Low | Low | High | High | Low | Low |
| Gyhagen (2015) [9] | Low | Low | High | High | Low | Low |
| Hall (1989)  [10] | Low | Low | Low | Unclear | Unclear | Low |
| Huttly (1990)  [11] | Low | High | Low | Unclear | Low | Low |
| Kjerulff (2013) [12] | Low | Low | Low | Unclear | Unclear | Low |
| Liang (2013)  [13] | Low | High | Low | Unclear | High | Low |
| MacArthur (2011)  [14] | Low | Low | High | High | Low | Low |
| McDonald (2015)  [15] | Low | Low | Low | High | High | Low |
| Mollison (2005)  [16] | Low | High | Low | Unclear | Low | Low |
| Murphy (2002) [17] | Low | Low | Low | Unclear | Low | Low |
| O’Neill (2014)  [18] | Low | Low | Low | Unclear | Low | Low |
| Rortveit (2003)  [19] | Low | Low | Unclear | Unclear | Unclear | Low |
| Schytt (2004)  [20] | Low | Low | High | High | High | Low |
| Smith (2006)  [21] | Low | Low | Low | Unclear | Low | Low |
| Tollanes (2007)  [22] | Low | High | Low | Unclear | Low | Low |
| Woolhouse (2012)  [23] | Low | Low | High | High | Low | Low |

Childhood outcomes studies

| Study | Selection of participants | Confounding variables | Measurement of exposure | Blinding of outcome assessments | Incomplete outcome data | Selective outcome reporting |
| --- | --- | --- | --- | --- | --- | --- |
| Ajslev (2011)  [24] | Low | Low | High | Unclear | High | Low |
| Almqvist (2012) [25] | Low | Low | Low | Unclear | Unclear | Low |
| Andersen (2013) [26] | Low | Low | Low | Unclear | Unclear | Low |
| Bager (2012) [27] | Low | Low | Low | Unclear | Unclear | Low |
| Barros (2012) [28] | Low | Low | Low | High | Low | Low |
| Black (2015) [29] | Low | Low | Low | Unclear | Low | Low |
| Changzheng (2016) | Low | Low | High | High | Unclear | Low |
| Davidson (2010) [30] | Low | Low | Low | Unclear | Low | Low |
| Eggesbo (2003) [31] | Low | Low | High | Low | Unclear | Low |
| Eggesbo (2005) [32] | Low | Low | Low | Unclear | Unclear | Low |
| Goldani (2011)  [33] | Low | Low | Low | Low | Low | Low |
| Goldani (2013)  [34] | Low | Low | Low | High | High | Low |
| Huh (2012) [35] | Low | Low | Low | High | Low | Low |
| Kero (2002) [36] | Low | High | Low | Unclear | Unclear | Low |
| Li (2013) [37] | Low | Low | Low | High | High | Low |
| Lin (2013) [38] | Low | Low | Low | High | Low | Low |
| McKeever (2002) [39] | Low | Low | Low | Unclear | Unclear | Low |
| Magnus (2011) [40] | Low | Low | High | Unclear | High | Low |
| Maitra (2004) [41] | Low | Low | High | High | High | Low |
| Mamun (2013)  [42] | Low | Low | Low | High | Unclear | Low |
| Menezes (2010) [43] | Low | Low | High | High | Low | Low |
| Mesquita (2013)  [44] | Low | Low | Low | High | High | Low |
| Negele (2004) [45] | Low | Low | Low | Unclear | High | Low |
| Pei (2014)  [46] | Low | Low | Low | High | High | Low |
| Ponsonby (2009) [47] | Low | Low | Low | Low | Low | Low |
| Pyrhonen (2013) [48] | Low | Low | High | Unclear | High | Low |
| Roberts (2011) [49] | Low | Low | Low | Unclear | Low | Low |
| Roduit (2009) [50] | Low | Low | High | Unclear | High | Low |
| Steur (2011)  [51] | Low | High | Low | High | High | Low |
| Tollanes (2008) [52] | Low | Low | Low | Unclear | Low | Low |
| Van Berkel (2015) [53] | Low | Low | High | High | High | Low |
| Van Nimwegen (2011) [54] | Low | Low | High | Unclear | Low | Low |
| Werner (2006) [55] | Low | Low | High | High | High | Low |
| Xu (2000) [56] | Low | Low | Low | High | Unclear | Low |
| Xu (2001) [57] | Low | Low | Unclear | High | Unclear | Low |

Subsequent pregnancies outcomes studies

| Study | Selection of participants | Confounding variables | Measurement of exposure | Blinding of outcome assessments | Incomplete outcome data | Selective outcome reporting |
| --- | --- | --- | --- | --- | --- | --- |
| Bowman (2015) [58] | Low | Low | Low | Unclear | Unclear | Low |
| Daltveit (2008)  [59] | Low | Low | Low | Unclear | Unclear | Low |
| Downes (2015) [60] | Low | Low | Low | Unclear | Low | Low |
| Galyean (2009)  [61] | Low | High | Unclear | Unclear | Low | High |
| Getahun (2006) [62] | Low | Low | Low | Unclear | Low | Low |
| Gray (2007)[63] | Low | Low | Low | Unclear | Low | Low |
| Gurol-Urganci (2011) [64] | Low | Low | Low | Unclear | Low | Low |
| Hall (1989)  [10] | Low | Low | Low | Unclear | Unclear | Low |
| Hemminki (2005) [65] | Low | Low | Low | Unclear | Unclear | Low |
| Huang (2011) [66] | Low | Low | Low | Unclear | Unclear | Low |
| Jackson (2012) [67] | Low | Low | Low | Unclear | Unclear | Low |
| Kennare (2007) [68] | Low | Low | Low | Unclear | Unclear | High |
| Lydon-Rochelle (2001) [69] | Low | Low | Low | Unclear | Unclear | Low |
| Mollison (2005)  [16] | Low | Low | Low | Unclear | Low | Low |
| Moraitis (2015) [70] | Low | Low | Low | Unclear | Unclear | Low |
| Osborne (2012) [71] | Low | High | Low | Unclear | Low | Low |
| Rasmussen (2000) [72] | Low | Low | Low | Unclear | Low | Low |
| Salihu (2006) [73] | Low | Low | Low | Unclear | Unclear | Low |
| Salihu (2011) [74] | Low | Low | Low | Unclear | Unclear | Low |
| Smith (2003) [75] | Low | High | Low | Unclear | Low | Low |
| Smith (2006)  [21] | Low | Low | Low | Unclear | Low | Low |
| Taylor (2005) [76] | Low | Low | Low | Unclear | Low | Low |
| Wood (2008) [77] | Low | Low | Low | Low | Low | Low |
| Yang (2007) [78] | Low | Low | Low | Unclear | Unclear | Low |

References

1. Abdel-Fattah M, Familusi A, Fielding S, Ford J, Bhattacharya S. Primary and repeat surgical treatment for female pelvic organ prolapse and incontinence in parous women in the UK: a register linkage study. BMJ Open. 2011;1:e000206. doi: 10.1136/bmjopen-2011-00206.

2. Bjelland EK, Owe KM, Pingel R, Kristiansson P, Vangen S, Eberhard-Gran M. Pelvic pain after childbirth: a longitudinal population study. Pain. 2016;157(3):710-6. doi: 10.1097/j.pain.0000000000000427. PubMed PMID: WOS:000378258800022.

3. Brown SJ, Gartland D, Donath S, MacArthur C. Fecal Incontinence During the First 12 Months Postparum. Obstet Gynecol. 2012;119:240-9. doi: 10.1097/AOG.0b013e318242b1f7.

4. Elvander C, Dahlberg J, Andersson G, Cnattingius S. Mode of delivery and the probability of subsequent childbearing: a population-based register study. Bjog-Int J Obstet Gy. 2015;122(12):1593-600. doi: 10.1111/1471-0528.13021. PubMed PMID: WOS:000363729300032.

5. Fussing-Clausen C, Geirsson RT, Hansen T, Rasmussen S, Lidegaard O, Hedegaard M. Mode of delivery and subsequent reproductive patterns. A national follow-up study. Acta Obstet Gyn Scan. 2014;93(10):1034-41. doi: 10.1111/aogs.12469. PubMed PMID: WOS:000342582800012.

6. Gartland D, Donath S, MacArthur C, Brown SJ. The onset, recurrence and associated obstetric risk factors for urinary incontinence in the first 18 months after a first birth: an Australian nulliparous cohort study. BJOG. 2012;119(11):1361-9. doi: 10.1111/j.1471-0528.2012.03437.x. PubMed PMID: 22827735.

7. Gyhagen M, Bullarbo M, Nielsen TF, Milsom I. Prevalence and risk factors for pelvic organ prolapse 20 years after childbirth: a national cohort study in singleton primiparae after vaginal or caesarean delivery. BJOG. 2013;120(2):152-60. doi: 10.1111/1471-0528.12020. PubMed PMID: 23121158.

8. Gyhagen M, Bullarbo M, Nielsen TF, Milsom I. The prevalence of urinary incontinence 20 years after childbirth: a national cohort study in singleton primiparae after vaginal or caesarean delivery. BJOG. 2013;120(2):144-51. doi: 10.1111/j.1471-0528.2012.03301.x. PubMed PMID: 22413831.

9. Gyhagen M, Akervall S, Milsom I. Clustering of pelvic floor disorders 20 years after one vaginal or one cesarean birth. Int Urogynecol J. 2015;26(8):1115-21. doi: 10.1007/s00192-015-2663-3. PubMed PMID: WOS:000361229500005.

10. Hall MH, Campbell D, Fraser C, Lemon J. Mode of delivery and future fertility. British Journal of Obstetrics and Gynaecology. 1989;96:1297-303.

11. Huttly SRA, Barros FC, Victora CG, Lombardi C, Vaughan JP. Subsequent pregnancies: Who has them and who wants them? Observations from an urban center in Southern Brazil. Rev Saude publ. 1990;24(3):212-6.

12. Kjerulff KH, Zhu J, Weisman CS, Ananth CV. First birth Caesarean section and subsequent fertility: a population-based study in the USA, 2000-2008. Hum Reprod. 2013;28(12):3349-57. doi: 10.1093/humrep/det343. PubMed PMID: 24021550; PubMed Central PMCID: PMC3829579.

13. Liang CC, Wu MP, Lin SJ, Lin YJ, Chang SD, Wang HH. Clinical impact of and contributing factors to urinary incontinence in women 5 years after first delivery. Int Urogynecol J. 2013;24(1):99-104. doi: 10.1007/s00192-012-1855-3. PubMed PMID: 22777581.

14. MacArthur C, Glazener C, Lancashire R, Herbison P, Wilson D. Exclusive caesarean section delivery and subsequent urinary and faecal incontinence: a 12-year longitudinal study. BJOG: An International Journal of Obstetrics & Gynaecology. 2011;118(8):1001-7. doi: 10.1111/j.1471-0528.2011.02964.x.

15. McDonald EA, Gartland D, Small R, Brown SJ. Dyspareunia and Childbirth: A Prospective Cohort Study. Obstet Gynecol Surv. 2015;70(5):319-20. PubMed PMID: WOS:000354725300013.

16. Mollison J, Porter M, Campbell D, Bhattacharya S. Primary mode of delivery and subsequent pregnancy. BJOG. 2005;112(8):1061-5. doi: 10.1111/j.1471-0528.2005.00651.x. PubMed PMID: 16045518.

17. Murphy DJ, Stirrat GM, Heron J, Team AS. The relationship between Caesarean section and subfertility in a population-based sample of 14 541 pregnancies. Hum Reprod. 2002;17(7):1914-7.

18. O'Neill SM, Khashan AS, Henriksen TB, Kenny LC, Kearney PM, Mortensen PB, et al. Does a Caesarean section increase the time to a second live birth? A register-based cohort study. Human Reproduction. 2014;29(11):2560-8. doi: 10.1093/humrep/deu217. PubMed PMID: WOS:000344675700026.

19. Rortveit G, Daltveit AK, Hannestad YS, Hunskaar S, Study NE. Urinary incontinence after vaginal delivery or cesarean section. New Engl J Med. 2003;348(10):900-7. doi: DOI 10.1056/NEJMoa021788. PubMed PMID: WOS:000181341100005.

20. Schytt E, Linkmark G, Waldenstrom U. Symptoms of stress incontinence 1 year after childbirth: prevalence and predictors in a national Swedish sample. Acta Obstet Gynecol Scand. 2004;83:928-36.

21. Smith GC, Wood AM, Pell JP, Dobbie R. First cesarean birth and subsequent fertility. Fertil Steril. 2006;85(1):90-5. Epub 2006/01/18. doi: S0015-0282(05)03433-3 [pii]

10.1016/j.fertnstert.2005.07.1289. PubMed PMID: 16412736.

22. Tollanes MC, Melve KK, Irgens LM, Skjaerven R. Reduced Fertility After Cesarean Delivery. Obstet Gynecol. 2007;110:1256-63.

23. Woolhouse H, Perlen S, Gartland D, Brown SJ. Physical Health and Recovery in the First 18 Months Postpartum: Does Cesarean Section Reduce Long-Term Morbidity? BIRTH. 2012;39(3):221-9.

24. Ajslev TA, Andersen CS, Gamborg M, Sorensen TI, Jess T. Childhood overweight after establishment of the gut microbiota: the role of delivery mode, pre-pregnancy weight and early administration of antibiotics. Int J Obes (Lond). 2011;35(4):522-9. doi: 10.1038/ijo.2011.27. PubMed PMID: 21386800.

25. Almqvist C, Cnattingius S, Lichtenstein P, Lundholm C. The impact of birth mode of delivery on childhood asthma and allergic diseases--a sibling study. Clin Exp Allergy. 2012;42(9):1369-76. doi: 10.1111/j.1365-2222.2012.04021.x. PubMed PMID: 22925323; PubMed Central PMCID: PMC3564396.

26. Andersen V, Erichsen R, Froslev T, Sorensen HT, Ehrenstein V. Differential risk of ulcerative colitis and Crohn's disease among boys and girls after cesarean delivery. Inflamm Bowel Dis. 2013;19(1):E8-E10. doi: 10.1002/ibd.22841. PubMed PMID: 22147542.

27. Bager P, Simonsen J, Nielsen NM, Frisch M. Cesarean section and offspringʼs risk of inflammatory bowel disease: A national cohort study. Inflammatory Bowel Diseases. 2012;18(5):857-62. doi: 10.1002/ibd.21805.

28. Barros FC, Matijasevich A, Hallal PC, Horta BL, Barros AJ, Menezes AB, et al. Cesarean section and risk of obesity in childhood, adolescence, and early adulthood: evidence from 3 Brazilian birth cohorts. Am J Clin Nutr. 2012;95(2):465-70. doi: 10.3945/ajcn.111.026401. PubMed PMID: 22237058; PubMed Central PMCID: PMC3260073.

29. Black M, Bhattacharya S, Philip S, Norman JE, McLernon DJ. Planned Cesarean Delivery at Term and Adverse Outcomes in Childhood Health. Jama-J Am Med Assoc. 2015;314(21):2271-9. doi: 10.1001/jama.2015.16176. PubMed PMID: WOS:000365515700019.

30. Davidson R, Roberts SE, Wotton CJ, Goldacre MJ. Influence of maternal and perinatal factors on subsequent hospitalisation for asthma in children: evidence from the Oxford record linkage study. Bmc Pulm Med. 2010;10. doi: Artn 14

10.1186/1471-2466-10-14. PubMed PMID: WOS:000208592700014.

31. Eggesbø M, Botten G, Stigum H, Nafstad P, Magnus P. Is delivery by cesarean section a risk factor for food allergy? Journal of Allergy and Clinical Immunology. 2003;112(2):420-6. doi: 10.1067/mai.2003.1610.

32. Eggesbo M, Botten G, Stigum H, Samuelsen SO, Brunekreef B, Magnus P. Cesarean delivery and cow milk allergy/intolerance. Allergy. 2005;60(9):1172-3. doi: 10.1111/j.1398-9995.2005.00857.x. PubMed PMID: 16076303.

33. Goldani HA, Bettiol H, Barbieri MA, Silva AA, Agranonik M, Morais MB, et al. Cesarean delivery is associated with an increased risk of obesity in adulthood in a Brazilian birth cohort study. Am J Clin Nutr. 2011;93(6):1344-7. doi: 10.3945/ajcn.110.010033. PubMed PMID: 21508088.

34. Goldani MZ, Barbieri MA, Moura da Silva AA, Pereria Gutierrez MR, Bettiol H, Goldani HA. Cesarean section and increased body mass index in school children: two cohort studies from distinct socioeconomic background areas in Brazil. Nutrition Journal. 2013;12.

35. Huh SY, Rifas-Shiman SL, Zera CA, Edwards JW, Oken E, Weiss ST, et al. Delivery by caesarean section and risk of obesity in preschool age children: a prospective cohort study. Archives of disease in childhood. 2012;97(7):610-6. Epub 2012/05/25. doi: 10.1136/archdischild-2011-301141. PubMed PMID: 22623615; PubMed Central PMCID: PMC3784307.

36. Kero J, Gissler M, Minna-Maija G, Kero P, Koskinen P, Hemminki E, et al. Mode of Delivery and Asthma - Is There a Connection? Pediatr Res. 2002;52(1):6-11. doi: 10.1023/01.PDR.0000017262.01840.F0.

37. Li HT, Ye R, Pei L, Ren A, Zheng X, Liu JM. Caesarean delivery, caesarean delivery on maternal request and childhood overweight: a Chinese birth cohort study of 181 380 children. Pediatr Obes. 2014;9(1). Epub 2013 Mar 19.

38. Lin SL, Schooling CM, Leung GM. Mode of Delivery and Adiposity: Hong Kong's "Children of 1997" Birth Cohort. Am J Epidemiol. 2013;177:S8-S. PubMed PMID: WOS:000319870300031.

39. McKeever TM, Lewis SA, Smith C, Hubbard R. Mode of delivery and risk of developing allergic disease. Journal of Allergy and Clinical Immunology. 2002;109(5):800-2. doi: 10.1067/mai.2002.124046.

40. Magnus MC, Haberg SE, Stigum H, Nafstad P, London SJ, Vangen S, et al. Delivery by Cesarean section and early childhood respiratory symptoms and disorders: the Norwegian mother and child cohort study. Am J Epidemiol. 2011;174(11):1275-85. Epub 2011/11/01. doi: 10.1093/aje/kwr242. PubMed PMID: 22038100; PubMed Central PMCID: PMC3254156.

41. Maitra A, Sherriff A, Strachan D, Team AS, Henderson J. Mode of delivery is not associated with asthma or atopy in childhood. Clin Exp Allergy. 2004;34:1349-55.

42. Mamun AA, Sutharsan R, O'Callaghan M, Williams G, Najman J, McIntyre HD, et al. Cesarean delivery and the long-term risk of offspring obesity. Obstet Gynecol. 2013;122(6):1176-83. doi: 10.1097/AOG.0000000000000016. PubMed PMID: 24201680.

43. Menezes AM, Hallal PC, Matijasevich AM, Barros AJ, Horta BL, Araujo CL, et al. Caesarean sections and risk of wheezing in childhood and adolescence: data from two birth cohort studies in Brazil. Clin Exp Allergy. 2011;41(2):218-23. doi: 10.1111/j.1365-2222.2010.03611.x. PubMed PMID: 20840395; PubMed Central PMCID: PMC3505367.

44. Schooling CM, Mesquita DN, Barbieri MA, Goldani HAS, Cardoso VC, Goldani MZ, et al. Cesarean Section Is Associated with Increased Peripheral and Central Adiposity in Young Adulthood: Cohort Study. PLoS ONE. 2013;8(6):e66827. doi: 10.1371/journal.pone.0066827.

45. Negele K, Heinrick J, Borte M, von Berg A, Schaaf B, Lehmann I, et al. Mode of delivery and development of atopic disease during the first 2 years of life. Pediatric Allergy and Immunology. 2004;15:48-54.

46. Pei Z, Heinrich J, Fuertes E, Flexeder C, Hoffmann B, Lehmann I, et al. Cesarean delivery and risk of childhood obesity. J Pediatr. 2014;164(5):1068-73 e2. doi: 10.1016/j.jpeds.2013.12.044. PubMed PMID: 24508442.

47. Ponsonby AL, Catto-Smith AG, Pezic A, Dupuis S, Halliday J, Cameron D, et al. Association between early-life factors and risk of child-onset Crohn's disease among Victorian children born 1983-1998: a birth cohort study. Inflamm Bowel Dis. 2009;15(6):858-66. doi: 10.1002/ibd.20842. PubMed PMID: 19107784.

48. Pyrhonen K, Nayha S, Hiltunen L, Laara E. Caesarean section and allergic manifestations: insufficient evidence of association found in population-based study of children aged 1 to 4 years. Acta Paediatr. 2013;102(10):982-9. doi: 10.1111/apa.12342. PubMed PMID: 23826787.

49. Roberts SE, Wotton CJ, Williams JG, Griffith M, Goldacre MJ. Perinatal and early life risk factors for inflammatory bowel disease. World J Gastroenterol. 2011;17(6):743-9. doi: 10.3748/wjg.v17.i6.743. PubMed PMID: 21390144; PubMed Central PMCID: PMC3042652.

50. Roduit C, Scholtens S, de Jongste JC, Wijga AH, Gerritsen J, Postma DS, et al. Ashtma at 8 years of age in children born by caesarean section. Thorax. 2009;64:107-13. doi: 10.1136/thx.2008.100875.

51. Steur M, Smit HA, Schipper CMA, Scholtens S, Kerkhof M, de Jongste JC, et al. Predicting the risk of newborn children to become overweight later in childhood: the PIAMA birth cohort study. Int J Pediatr Obes. 2011;6:e170-e8.

52. Tollanes MC, Moster D, Daltveit AK, Irgens LM. Cesarean section and risk of severe childhood asthma: a population-based cohort study. J Pediatr. 2008;153(1):112-6. doi: 10.1016/j.jpeds.2008.01.029. PubMed PMID: 18571547.

53. van Berkel AC, den Dekker HT, Jaddoe VWV, Reiss IK, Gaillard R, Hofman A, et al. Mode of delivery and childhood fractional exhaled nitric oxide, interrupter resistance and asthma: the Generation R study. Pediatric Allergy and Immunology. 2015;26(4):330-6. doi: 10.1111/pai.12385. PubMed PMID: WOS:000355149800005.

54. van Nimwegen FA, Penders J, Stobberingh EE, Postma DS, Koppelman GH, Kerkhof M, et al. Mode and place of delivery, gastrointestinal microbiota, and their influence on asthma and atopy. J Allergy Clin Immunol. 2011;128(5):948-55 e1-3. doi: 10.1016/j.jaci.2011.07.027. PubMed PMID: 21872915.

55. Werner A, Ramlau-Hansen CH, Jeppesen SK, Thulstrup AM, Olsen J. Caesarean delivery and risk of developing asthma in the offspring. Acta Paediatr. 2007;96(4):595-6. doi: 10.1111/j.1651-2227.2006.00150.x. PubMed PMID: 17274805.

56. Xu B, Pekkanen J, Jarvelin MR. Obstetric Complications and Asthma in Childhood. Journal of Asthma. 2000;37(7):589-94.

57. Xu B, Pekkanen J, Hartikainen AL, Jarvelin MR. Caesarean section and risk of asthma and allergy in adulthood. J Allergy Clin Immunol. 2001;107(4):732-3. doi: 10.1067/mai.2001.113048. PubMed PMID: 11295666.

58. Bowman ZS SK, Silver RM. Cesarean Delivery and Risk for Subsequent Ectopic Pregnancy. American journal of perinatology 2015;32(9):815-20.

59. Daltveit AK, Tollanes MC, Pihlstrom H, Irgens LM. Cesarean Delivery and Subsequent Pregnancies. Obstet Gynecol. 2008;111:1327-34.

60. Downes KL HS, Sjaarda LA, et al. Previous prelabor or intrapartum cesarean delivery and risk of placenta previa. Am J Obstet Gynecol 2015;212(5):669 e1-6.

61. Galyean AM, Lagrew DC, Bush MC, Kurtzman JT. Previous cesarean section and the risk of postpartum maternal complications and adverse neonatal outcomes in future pregnancies. Journal of Perinatology. 2009;29:726-30.

62. Getahun D, Oyelese Y, Salihu HM, Ananth CV. Previous cesarean delivery and risks of placenta previa and placental abruption. Obstet Gynecol. 2006;107(4):771-8.

63. Gray R, Quigley MA, Hockley C, Kurinczuk JJ, Goldacre M, Brocklehurst P. Caesarean delivery and risk of stillbirth in subsequent pregnancy: a retrospective cohort study in an English population. BJOG. 2007;114(3):264-70. doi: 10.1111/j.1471-0528.2006.01249.x. PubMed PMID: 17261119.

64. Gurol-Urganci I, Cromwell DA, Edozien LC, Smith GC, Onwere C, Mahmood TA, et al. Risk of placenta previa in second birth after first birth cesarean section: a population-based study and meta-analysis. BMC Pregnancy Childbirth. 2011;11:95. doi: 10.1186/1471-2393-11-95. PubMed PMID: 22103697; PubMed Central PMCID: PMC3247856.

65. Hemminki E, Shelley J, Gissler M. Mode of delivery and problems in subsequent births: a register-based study from Finland. Am J Obstet Gynecol. 2005;193(1):169-77. doi: 10.1016/j.ajog.2004.11.007. PubMed PMID: 16021075.

66. Huang X, Lei J, Tan H, Walker M, Zhou J, Wen SW. Cesarean delivery for first pregnancy and neonatal morbidity and mortality in second pregnancy. European Journal of Obstetrics & Gynaecology and Reproductive Biology. 2011;158:204-8.

67. Jackson S, Fleege L, Fridman M, Gregory K, Zelop C, Olsen J. Morbidity following primary cesarean delivery in the Danish National Birth Cohort. Am J Obstet Gynecol. 2012;206(2):139 e1-5. doi: 10.1016/j.ajog.2011.09.023. PubMed PMID: 22051815.

68. Kennare R, Tucker G, Heard A, Chan A. Risks of Adverse Outcomes in the Next Birth After a First Cesarean Delivery. Obstet Gynecol. 2007;109:270-6.

69. Lydon-Rochelle M, Holt VL, Easterling TR, Martin DP. First-Birth Cesarean and Placental Abruption or Previa at Second Birth. Obstet Gynecol. 2001;97:765-9.

70. Moraitis AA, Oliver-Williams C, Wood AM, Fleming M, Pell JP, Smith GCS. Previous caesarean delivery and the risk of unexplained stillbirth: retrospective cohort study and meta-analysis. Bjog-Int J Obstet Gy. 2015;122(11):1467-74. doi: 10.1111/1471-0528.13461. PubMed PMID: WOS:000362752100009.

71. Osborne C, Ecker JL, Gauvreau K, Lieberman E. First birth cesarean and risk of antepartum fetal death in a subsequent pregnancy. J Midwifery Womens Health. 2012;57(1):12-7. doi: 10.1111/j.1542-2011.2011.00142.x. PubMed PMID: 22251907.

72. Rasmussen S, Albrechtsen S, Dalaker K. Obstetric history and the risk of placenta previa. Acta Obstet Gynecol Scand. 2000;79:502-7.

73. Salihu HM, Sharma PP, Kristensen S, Blot C, Alio AP, Ananth CV, et al. Risk of Stillbirth Following a Cesarean Delivery. Obstet Gynecol. 2006;107:383-90.

74. Salihu HM, Bowen CM, Wilson RE, Marty PJ. The impact of previous cesarean section on the success of future fetal programming pattern. Arch Gynecol Obstet. 2011;284(2):319-26. doi: 10.1007/s00404-010-1665-0. PubMed PMID: 20821225.

75. Smith GC, Pell JP, Dobbie R. Caesarean section and risk of unexplained stillbirth in subsequent pregnancy. Lancet. 2003;362(9398):1779-84. Epub 2003/12/05. doi: S0140673603148969 [pii]. PubMed PMID: 14654315.

76. Taylor LK, Simpson JM, Roberts CL, Olive EC, Henderson-Smart DJ. Risk of complications in a second pregnancy following caesarean section in the first pregnancy: a population-based study. MJA. 2005;183:515-9.

77. Wood SL, Chen S, Ross S, Sauve R. The risk of unexplained antepartum stillbirth in second pregnancies following caesarean section in the first pregnancy. BJOG: An International Journal of Obstetrics and Gynaecology. 2008;115(6):726-31. doi: 10.1111/j.1471-0528.2008.01705.x.

78. Yang Q, Wen SW, Oppenheimer L, Chen XK, Black D, Gao J, et al. Association of caesarean delivery for first birth with placenta praevia and placental abruption in second pregnancy. BJOG. 2007;114(5):609-13. doi: 10.1111/j.1471-0528.2007.01295.x. PubMed PMID: 17355267.
