# Supplementary material for: Long-term risks and benefits associated with cesarean delivery for mother, baby, and subsequent pregnancies: Systematic review and meta-analysis
Source: PLoS Med. 2018 Jan 23;15(1):e1002494. doi: 10.1371/journal.pmed.1002494 (PMC5779640; doi:10.1371/journal.pmed.1002494)
Supplement: S6 Table — (DOCX) [file pmed.1002494.s008.docx]

**S6 Table: Non-prespecified childhood outcomes after cesarean delivery compared to vaginal delivery**

| Outcome | Studies | Participants | Statistical Method | Effect Estimate |
| --- | --- | --- | --- | --- |
| Type 1 Diabetes [1] [2] [3] [4] [5] | 5 | 3357381 | Odds Ratio (M-H, Random, 95% CI) | 1.19 [1.05, 1.35] |
| Celiac disease [6] [1] | 2 | 1010079 | Odds Ratio (M-H, Random, 95%CI) | 0.66 [0.21, 2.08] |

S6 Table: Table showing analyses and effect estimates of non-prespecified outcomes in children delivered by cesarean section compared with children delivered by vaginal delivery.

**References**

1. Adlercreutz EH, Svensson J, Hansen D, Buschard K, Lernmark A, Mortensen HB, et al. Prevalence of celiac disease autoimmunity in children with type 1 diabetes: regional variations across the Oresund strait between Denmark and southernmost Sweden. Pediatr Diabetes. 2015;16(7):504-9. doi: 10.1111/pedi.12200. PubMed PMID: WOS:000362550400004.

2. Black M, Bhattacharya S, Philip S, Norman JE, McLernon DJ. Planned Cesarean Delivery at Term and Adverse Outcomes in Childhood Health. Jama-J Am Med Assoc. 2015;314(21):2271-9. doi: 10.1001/jama.2015.16176. PubMed PMID: WOS:000365515700019.

3. Cardwell CR, Carson DJ, Patterson CC. Parental age at delivery, birth order, birth weight and gestational age are associated with the risk of childhood Type 1 diabetes: a UK regional retrospective cohort study. Diabetic Med. 2005;22(2):200-6. doi: DOI 10.1111/j.1464-5491.2005.01369.x. PubMed PMID: WOS:000226475700014.

4. Algert CS, McElduff A, Morris JM, Roberts CL. Perinatal risk factors for early onset of Type 1 diabetes in a 2000-2005 birth cohort. Diabetic Med. 2009;26(12):1193-7. doi: 10.1111/j.1464-5491.2009.02878.x. PubMed PMID: WOS:000272161900002.

5. Stene LC, Magnus P, Lie RT, Sovik O, Joner G, Norwegian Childhood Diabetes Study G. No association between preeclampsia or cesarean section and incidence of type 1 diabetes among children: a large, population-based cohort study. Pediatr Res. 2003;54(4):487-90. doi: 10.1203/01.PDR.0000081301.25600.5D. PubMed PMID: 12815116.

6. Roberts SE, Williams JG, Meddings D, Davidson R, Goldacre MJ. Perinatal risk factors and coeliac disease in children and young adults: a record linkage study. Aliment Pharm Ther. 2009;29(2):222-31. doi: 10.1111/j.1365-2036.2008.03871.x. PubMed PMID: WOS:000261781900009.
