# Supplementary material for: Long-term risks and benefits associated with cesarean delivery for mother, baby, and subsequent pregnancies: Systematic review and meta-analysis
Source: PLoS Med. 2018 Jan 23;15(1):e1002494. doi: 10.1371/journal.pmed.1002494 (PMC5779640; doi:10.1371/journal.pmed.1002494)
Supplement: S7 Table — (DOCX) [file pmed.1002494.s009.docx]

**S7 Table: Summary of Associations and Numbers Needed to Treat for Benefit or Harm**

| ***Number of cesareans needed for benefit*** | | | | |
| --- | --- | --- | --- | --- |
| **Outcome** | **Incidence per 1000 vaginal deliveries** | **Absolute Risk Reduction (%)** | **Number needed to treat for BENEFIT** | **95% Confidence Intervals** |
| MATERNAL OUTCOMES | | | | |
| Urinary incontinence | 149 | 6.0 | 17 | 14,22 |
| Pelvic organ prolapse | 59.9 | 4.2 | 24 | 10,35 |
| CHILDHOOD OUTCOMES | | | | |
| Child has inflammatory bowel disease (up to age 35 yrs) | 3.4 | 0.1 | 1092 | 951,1404 |
| SUBSEQUENT PREGNANCY OUTCOMES | | | | |
| Postpartum hemorrhage | 54 | 1.5 | 69 | 42,390 |
| ***Number of cesareans needed for harm*** | | | | |
| **Outcome** | **Incidence per 1000 vaginal deliveries** | **Absolute Risk Increase (%)** | **Number needed to treat for HARM** | **95% Confidence Intervals** |
| MATERNAL OUTCOMES | | | | |
| Subfertility | 318 | 10.9 | 9 | 8,12 |
| CHILDHOOD OUTCOMES | | | | |
| Child has asthma (up to age 12 yrs) | 30.5 | 0.6 | 162 | 107,308 |
| Child has obesity (age 6-15 yrs) | 91 | 3.6 | 28 | 16,82 |
| SUBSEQUENT PREGNANCY OUTCOMES | | | | |
| Placenta previa | 2.8 | 0.2 | 494 | 420, 589 |
| Placenta accreta | 0.3 | 0.1 | 1770 | 617, 10780 |
| Placental abruption | 5 | 0.2 | 534 | 415, 751 |
| Uterine rupture | 0.1 | 0.2 | 538 | 224, 1340 |
| Miscarriage | 95.8 | 1.4 | 69 | 37, 386 |
| Stillbirth | 3.3 | 0.1 | 1144 | 773, 2059 |
| Hysterectomy | 0.2 | 0.1 | 1561 | 342, 74092 |
| Antepartum haemorrhage | 16.7 | 0.4 | 278 | 170, 678 |

S7 Table: Summary table showing the incidence of each outcome per 1000 vaginal deliveries, the absolute risk reduction or absolute risk increase and the number needed to treat for harm or benefit, based on the prevalence rates and risk ratios from the systematic review data. This table shows for example, that if the associations found in this review were known to be causal, 19 cesarean sections would be needed to prevent one patient developing urinary incontinence, and out of every 70 cesarean sections performed, one woman will have a miscarriage that would not have otherwise.
