# Supplementary figures and images for: Long-term risks and benefits associated with cesarean delivery for mother, baby, and subsequent pregnancies: Systematic review and meta-analysis
Source: PLoS Med. 2018 Jan 23;15(1):e1002494. doi: 10.1371/journal.pmed.1002494 (PMC5779640; doi:10.1371/journal.pmed.1002494)

**S1 Figure: Study flow diagram of maternal outcomes database search**


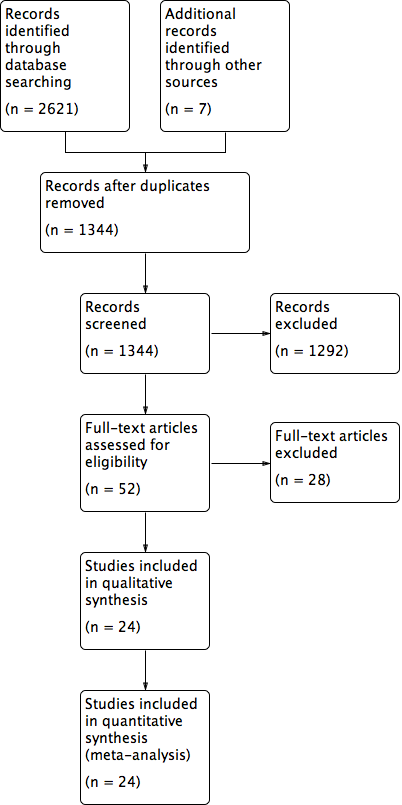

Supplement: S1 Fig — (DOCX) [file pmed.1002494.s010.docx]

**S2 Figure: Study flow diagram of childhood outcomes database search**


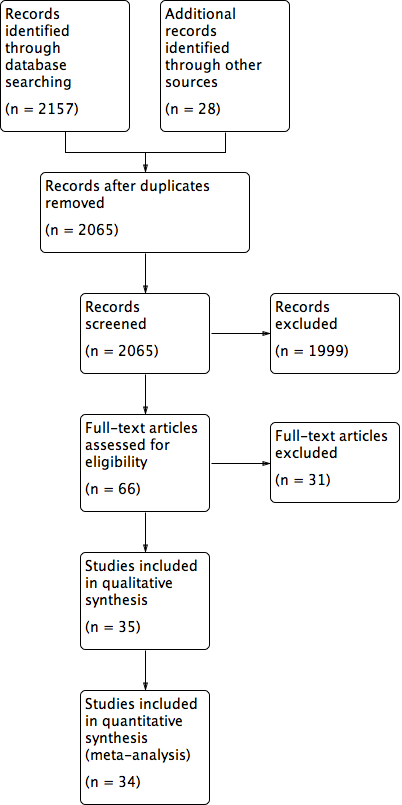

Supplement: S2 Fig — (DOCX) [file pmed.1002494.s011.docx]

**S3 Figure: Study flow diagram of subsequent pregnancy outcomes database search**


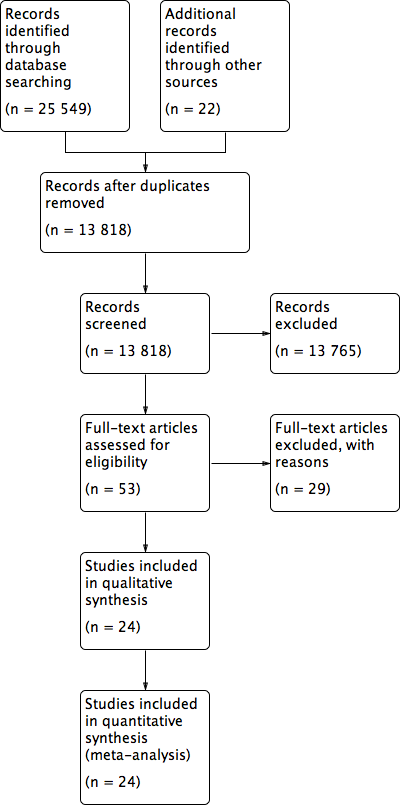

Supplement: S3 Fig — (DOCX) [file pmed.1002494.s012.docx]
