# Supplementary material for: Long-term risks and benefits associated with cesarean delivery for mother, baby, and subsequent pregnancies: Systematic review and meta-analysis
Source: PLoS Med. 2018 Jan 23;15(1):e1002494. doi: 10.1371/journal.pmed.1002494 (PMC5779640; doi:10.1371/journal.pmed.1002494)

S4 Figure: A random effects meta-analysis of urinary incontinence after cesarean delivery compared to vaginal delivery

Study or Subgroup	Odds Ratio M-H, Random, 95% CI
Abdel-fattah 2011	0.50 [0.37, 0.67]
Gartland 2012	0.42 [0.29, 0.59]
Gyhagen 2013b	0.64 [0.55, 0.73]
Liang 2013	0.56 [0.35, 0.92]
MacArthur 2011	0.80 [0.68, 0.95]
Rortveit 2003	0.59 [0.48, 0.73]
Schytt 2004	0.39 [0.27, 0.56]
Woolhouse 2012	0.53 [0.38, 0.73]
**Total (95% CI)**	**0.56 [0.47, 0.66]**
Total events	

Heterogeneity: $Tau^2 = 0.04$; $Chi^2 = 24.15$, df = 7 (P = 0.001); $I^2 = 71\%$

Test for overall effect: Z = 6.75 (P < 0.00001)

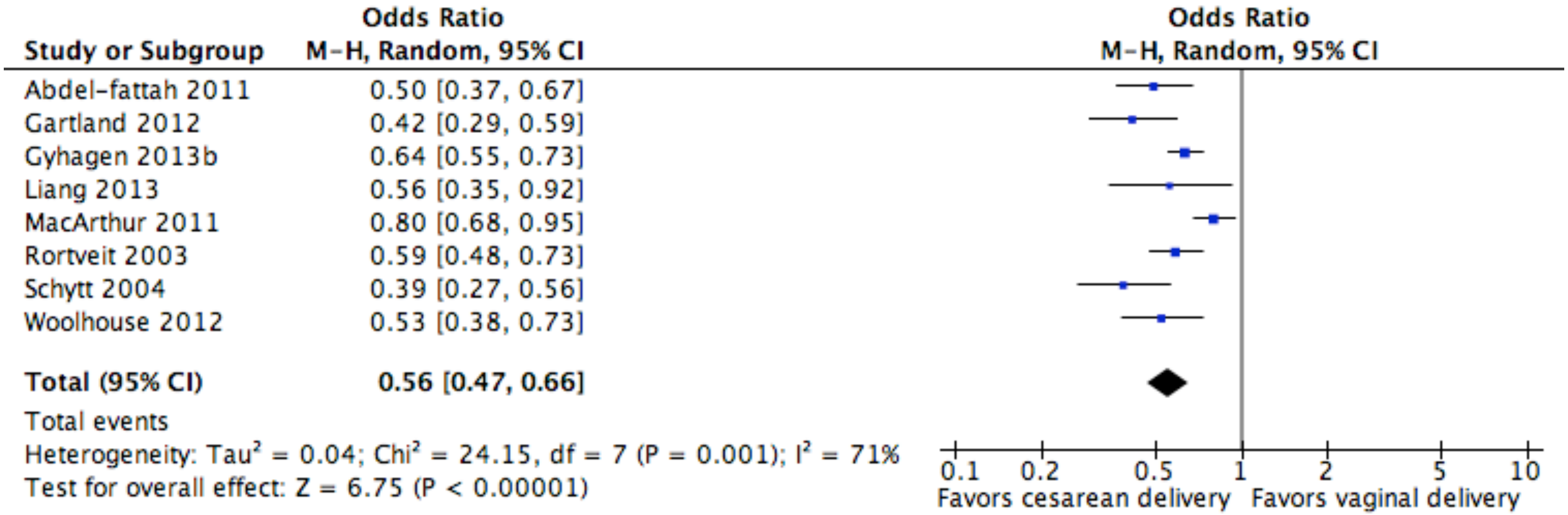

Supplement: S4 Fig — (PDF) [file pmed.1002494.s013.pdf]
