# Supplementary material for: Long-term risks and benefits associated with cesarean delivery for mother, baby, and subsequent pregnancies: Systematic review and meta-analysis
Source: PLoS Med. 2018 Jan 23;15(1):e1002494. doi: 10.1371/journal.pmed.1002494 (PMC5779640; doi:10.1371/journal.pmed.1002494)

S5 Figure: A random effects meta-analysis of pelvic organ prolapse after cesarean delivery compared to vaginal delivery

Study or Subgroup	Odds Ratio M-H, Random, 95% CI
Abdel-fattah 2011	0.22 [0.16, 0.30]
Gyhagen 2013a	0.38 [0.30, 0.49]
**Total (95% CI)**	**0.29 [0.17, 0.51]**
Total events	

Heterogeneity: $Tau^2 = 0.14$; $Chi^2 = 7.71$, df = 1 (P = 0.005); $I^2 = 87\%$
Test for overall effect: Z = 4.33 (P < 0.0001)

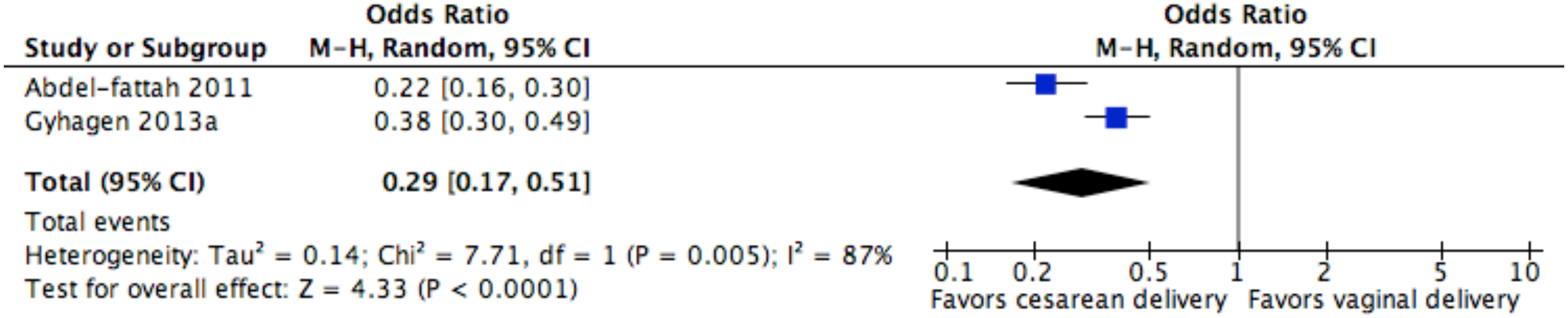

Supplement: S5 Fig — (PDF) [file pmed.1002494.s014.pdf]
