# Supplementary material for: Long-term risks and benefits associated with cesarean delivery for mother, baby, and subsequent pregnancies: Systematic review and meta-analysis
Source: PLoS Med. 2018 Jan 23;15(1):e1002494. doi: 10.1371/journal.pmed.1002494 (PMC5779640; doi:10.1371/journal.pmed.1002494)

S6 Figure: A random effects meta-analysis of fecal incontinence after cesarean delivery compared to vaginal delivery

Study or Subgroup	Odds Ratio M-H, Random, 95% CI	Odds Ratio M-H, Random, 95% CI
Abdel-fattah 2011	0.34 [0.12, 0.91]	
Brown 2012	0.83 [0.53, 1.31]	
Gyhagen 2015	1.46 [1.07, 1.98]	
MacArthur 2011	0.94 [0.73, 1.20]	
Woolhouse 2012	1.64 [1.02, 2.64]	
**Total (95% CI)**	**1.04 [0.73, 1.48]**	
Total events		

Heterogeneity: $Tau^2 = 0.11$; $Chi^2 = 14.50$, df = 4 (P = 0.006); $I^2 = 72\%$

Test for overall effect: Z = 0.22 (P = 0.82)

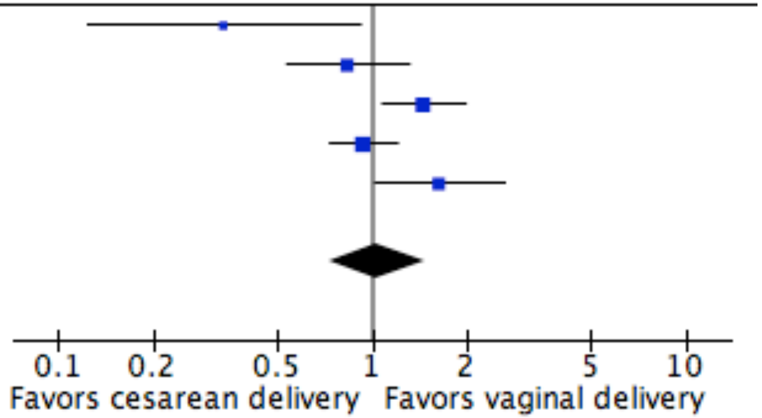

Supplement: S6 Fig — (PDF) [file pmed.1002494.s015.pdf]
