# Supplementary material for: Long-term risks and benefits associated with cesarean delivery for mother, baby, and subsequent pregnancies: Systematic review and meta-analysis
Source: PLoS Med. 2018 Jan 23;15(1):e1002494. doi: 10.1371/journal.pmed.1002494 (PMC5779640; doi:10.1371/journal.pmed.1002494)

S7 Figure: A fixed effects meta-analysis of pelvic pain after cesarean delivery compared to vaginal delivery

Study or Subgroup	Odds Ratio M–H, Fixed, 95% CI	Odds Ratio M–H, Fixed, 95% CI
Bjelland 2016	0.76 [0.53, 1.09]	
Woolhouse 2012	0.69 [0.39, 1.23]	
**Total (95% CI)**	**0.74 [0.54, 1.00]**	
Total events		
Heterogeneity: $Chi^2 = 0.07$, df = 1 (P = 0.80); $I^2 = 0\%$		
Test for overall effect: Z = 1.93 (P = 0.05)		

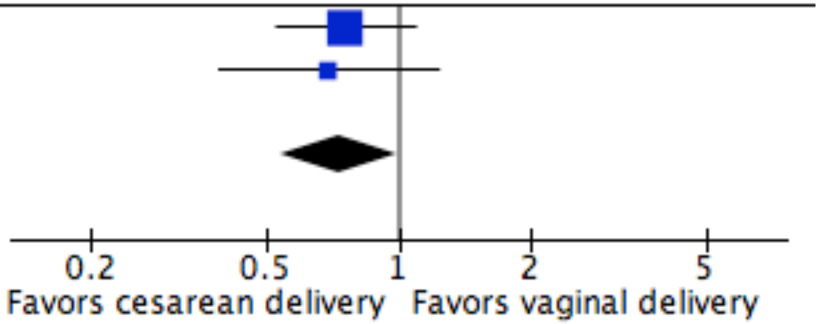

Supplement: S7 Fig — (PDF) [file pmed.1002494.s016.pdf]
