# Supplementary material for: Long-term risks and benefits associated with cesarean delivery for mother, baby, and subsequent pregnancies: Systematic review and meta-analysis
Source: PLoS Med. 2018 Jan 23;15(1):e1002494. doi: 10.1371/journal.pmed.1002494 (PMC5779640; doi:10.1371/journal.pmed.1002494)

S8 Figure: A random effects meta-analysis of no further pregnancy up to 28 years after cesarean delivery compared with vaginal delivery

Study or Subgroup	Odds Ratio M-H, Random, 95% CI
Elvander 2015	1.80 [1.78, 1.83]
Fussing-Clausen 2014	1.40 [1.39, 1.41]
Hall 1989	1.69 [1.54, 1.85]
Huttly 1990	1.39 [1.20, 1.62]
Kjerulff 2013	1.35 [1.30, 1.41]
Mollison 2005	1.35 [1.26, 1.46]
Murphy 2002	1.96 [1.01, 3.81]
O'Neill 2014	1.74 [1.72, 1.76]
Smith 2006	1.34 [1.29, 1.39]
Tollanes 2007a	2.23 [2.15, 2.31]
Tollanes 2007b	1.81 [1.77, 1.85]
**Total (95% CI)**	**1.60 [1.45, 1.76]**
Total events	

Heterogeneity: $Tau^2 = 0.02$; $Chi^2 = 1877.79$, df = 10 ($P < 0.00001$); $I^2 = 99\%$

Test for overall effect: Z = 9.56 ($P < 0.00001$)

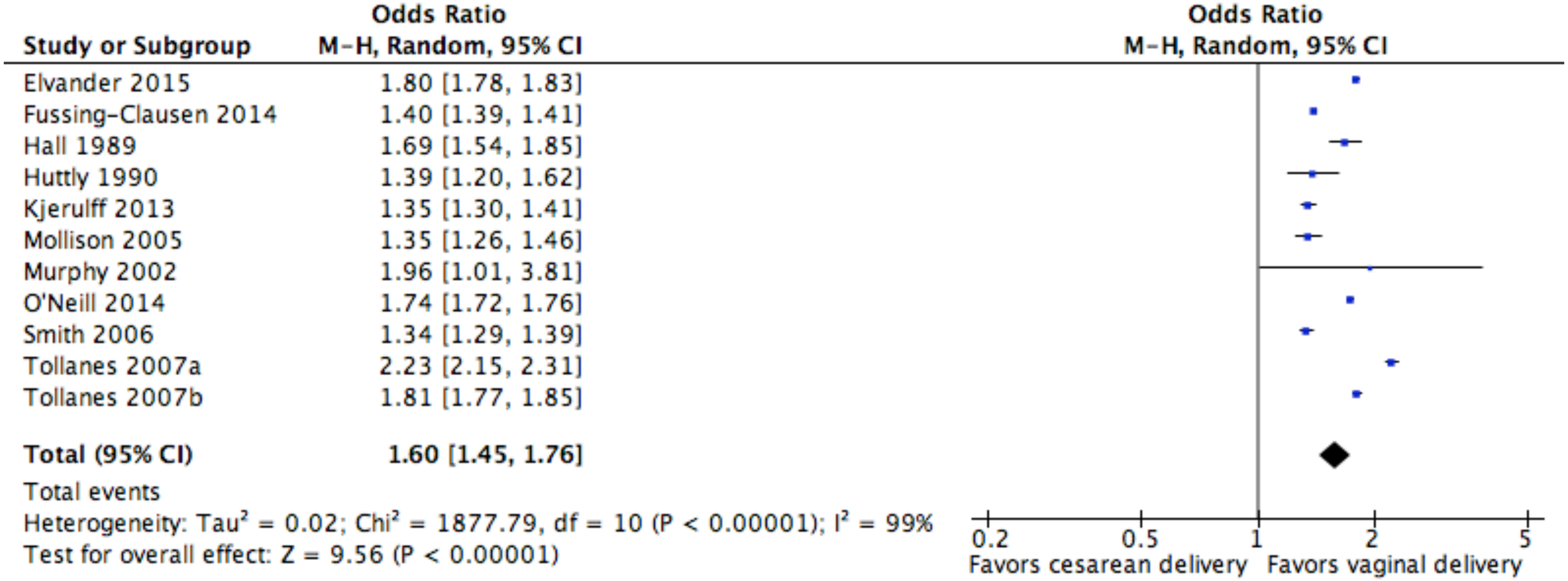

Supplement: S8 Fig — (PDF) [file pmed.1002494.s017.pdf]
