# Supplementary material for: Long-term risks and benefits associated with cesarean delivery for mother, baby, and subsequent pregnancies: Systematic review and meta-analysis
Source: PLoS Med. 2018 Jan 23;15(1):e1002494. doi: 10.1371/journal.pmed.1002494 (PMC5779640; doi:10.1371/journal.pmed.1002494)

**S9 Figure: A random effects meta-analysis of asthma in children up to 12 years old after cesarean delivery compared to vaginal delivery**

| Study or Subgroup | Odds Ratio M–H, Random, 95% CI |
|---|---|
| Almqvist 2012 | 1.33 [1.19, 1.50] |
| Black 2015 | 1.05 [1.00, 1.10] |
| Davidson 2010 | 1.20 [1.07, 1.35] |
| Kero 2002 | 1.27 [1.13, 1.42] |
| Magnus 2011 | 1.36 [1.22, 1.52] |
| Maitra 2004 | 1.16 [0.97, 1.39] |
| McKeever 2002 | 1.06 [0.98, 1.15] |
| Negele 2004a | 0.24 [0.03, 1.86] |
| Pyrhonen 2013 | 1.04 [0.65, 1.64] |
| Roduit 2009 | 1.92 [1.38, 2.67] |
| van Berkel 2015 | 1.10 [0.79, 1.54] |
| Van Nimwegen 2011 | 1.18 [0.69, 2.01] |
| Xu 2000 | 1.28 [0.94, 1.76] |
| **Total (95% CI)** | **1.21 [1.11, 1.32]** |
| Total events | |

Heterogeneity: $Tau^2 = 0.01$; $Chi^2 = 47.65$, df = 12 ($P < 0.00001$); $I^2 = 75\%$

Test for overall effect: $Z = 4.39$ ($P < 0.0001$)

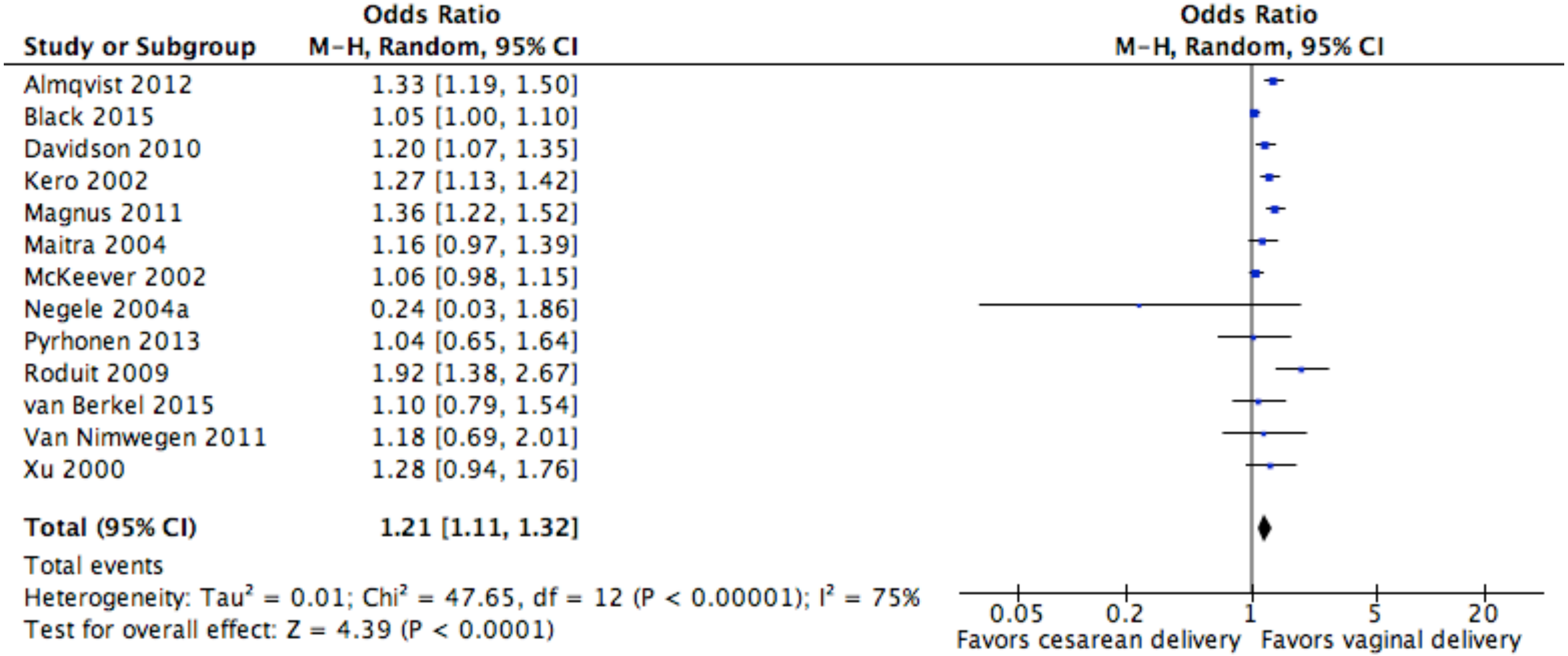

Supplement: S9 Fig — (PDF) [file pmed.1002494.s018.pdf]
