# Supplementary material for: Long-term risks and benefits associated with cesarean delivery for mother, baby, and subsequent pregnancies: Systematic review and meta-analysis
Source: PLoS Med. 2018 Jan 23;15(1):e1002494. doi: 10.1371/journal.pmed.1002494 (PMC5779640; doi:10.1371/journal.pmed.1002494)

S10 Figure: A random effects meta-analysis of asthma in adults after cesarean delivery compared to vaginal delivery

Study or Subgroup	Odds Ratio M-H, Random, 95% CI	Odds Ratio M-H, Random, 95% CI
Werner 2006	1.14 [0.91, 1.42]	
Xu 2001	3.31 [1.81, 6.05]	
**Total (95% CI)**	**1.87 [0.65, 5.32]**	
Total events		

Heterogeneity: $Tau^2 = 0.52$; $Chi^2 = 10.69$, df = 1 (P = 0.001); $I^2 = 91\%$
Test for overall effect: Z = 1.17 (P = 0.24)

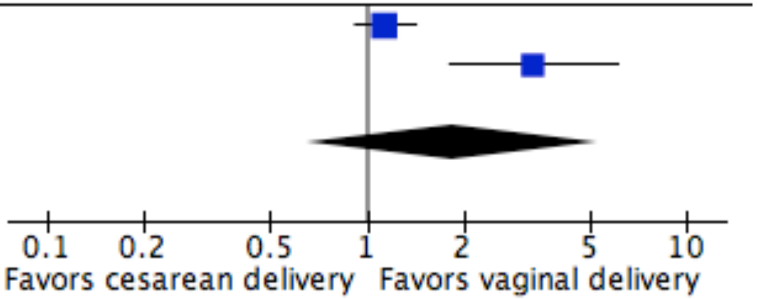

Supplement: S10 Fig — (PDF) [file pmed.1002494.s019.pdf]
