# Supplementary material for: Long-term risks and benefits associated with cesarean delivery for mother, baby, and subsequent pregnancies: Systematic review and meta-analysis
Source: PLoS Med. 2018 Jan 23;15(1):e1002494. doi: 10.1371/journal.pmed.1002494 (PMC5779640; doi:10.1371/journal.pmed.1002494)

S11 Figure: A random effects meta-analysis of wheeze in children up to 5 years old after cesarean delivery compared to vaginal delivery

Study or Subgroup	Odds Ratio M-H, Random, 95% CI
Magnus 2011	1.12 [1.05, 1.19]
Menezes 2010a	0.94 [0.82, 1.10]
Menezes 2010b	0.93 [0.82, 1.07]
Negele 2004a	1.38 [1.01, 1.88]
van Berkel 2015	1.38 [1.13, 1.68]
**Total (95% CI)**	**1.10 [0.96, 1.26]**
Total events	

Heterogeneity: $Tau^2 = 0.02$; $Chi^2 = 17.03$, df = 4 ($P = 0.002$); $I^2 = 77\%$

Test for overall effect: $Z = 1.41$ ($P = 0.16$)

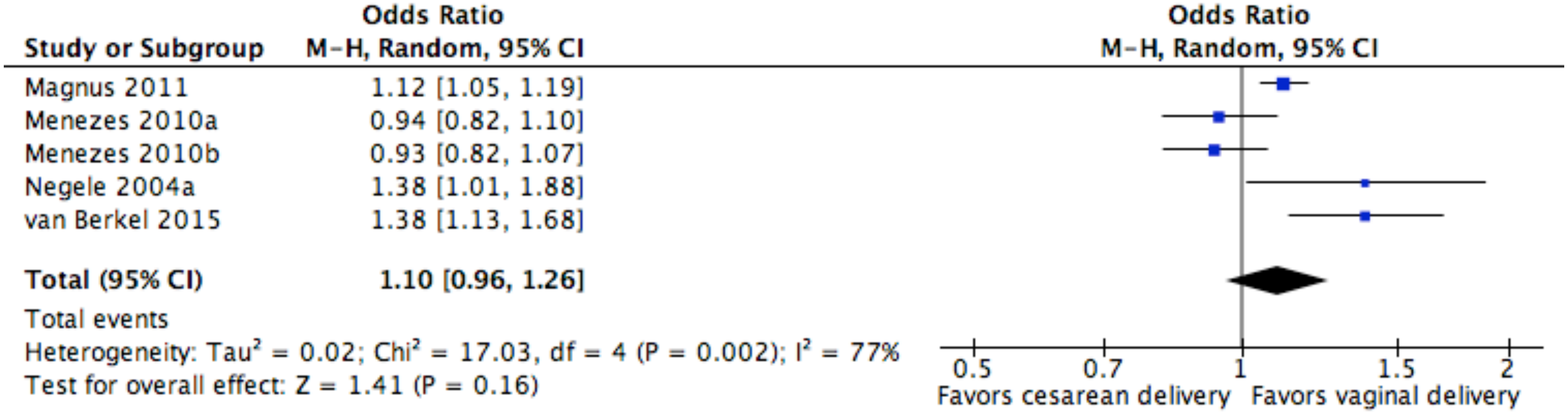

Supplement: S11 Fig — (PDF) [file pmed.1002494.s020.pdf]
