# Supplementary material for: Long-term risks and benefits associated with cesarean delivery for mother, baby, and subsequent pregnancies: Systematic review and meta-analysis
Source: PLoS Med. 2018 Jan 23;15(1):e1002494. doi: 10.1371/journal.pmed.1002494 (PMC5779640; doi:10.1371/journal.pmed.1002494)

S12 Figure: A fixed effects meta-analysis of wheeze in children 6-15 years old after cesarean delivery compared to vaginal delivery

Study or Subgroup	Odds Ratio M-H, Fixed, 95% CI
Maitra 2004	1.10 [0.87, 1.40]
Menezes 2010a	1.12 [0.94, 1.32]
Roduit 2009	1.36 [0.84, 2.21]
van Berkel 2015	1.42 [1.09, 1.83]
**Total (95% CI)**	**1.18 [1.05, 1.33]**
Total events	

Heterogeneity: $Chi^2 = 2.99$, df = 3 (P = 0.39); $I^2 = 0\%$

Test for overall effect: Z = 2.75 (P = 0.006)

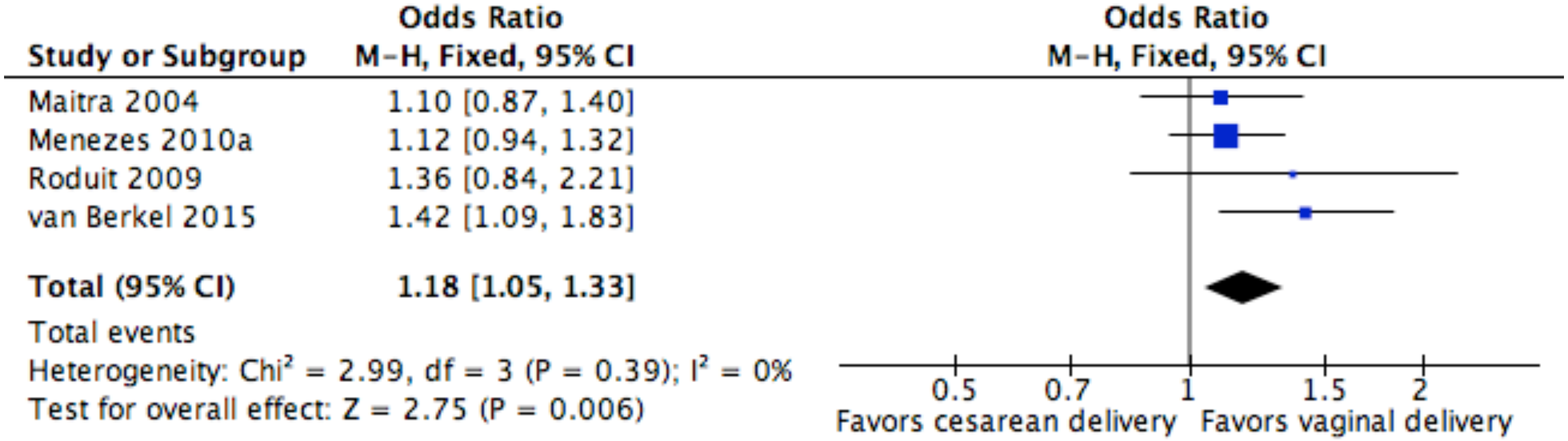

Supplement: S12 Fig — (PDF) [file pmed.1002494.s021.pdf]
