# Supplementary material for: Long-term risks and benefits associated with cesarean delivery for mother, baby, and subsequent pregnancies: Systematic review and meta-analysis
Source: PLoS Med. 2018 Jan 23;15(1):e1002494. doi: 10.1371/journal.pmed.1002494 (PMC5779640; doi:10.1371/journal.pmed.1002494)

S13 Figure: A random effects meta-analysis of hypersensitivity or allergy or dermatitis or atopy in children after cesarean delivery compared to vaginal delivery

Study or Subgroup	Odds Ratio M-H, Random, 95% CI
Eggesbo 2003b	3.15 [1.44, 6.89]
Eggesbo 2005	3.31 [0.85, 12.88]
Maitra 2004	1.08 [0.89, 1.32]
McKeever 2002	0.93 [0.78, 1.11]
Negele 2004b	1.27 [0.91, 1.78]
Pyrhonen 2013	1.16 [0.87, 1.57]
Roduit 2009	1.20 [0.83, 1.75]
Xu 2001	0.93 [0.60, 1.42]
**Total (95% CI)**	**1.15 [0.97, 1.36]**
Total events	

Heterogeneity: $Tau^2 = 0.03$; $Chi^2 = 14.54$, df = 7 (P = 0.04); $I^2 = 52\%$

Test for overall effect: Z = 1.56 (P = 0.12)

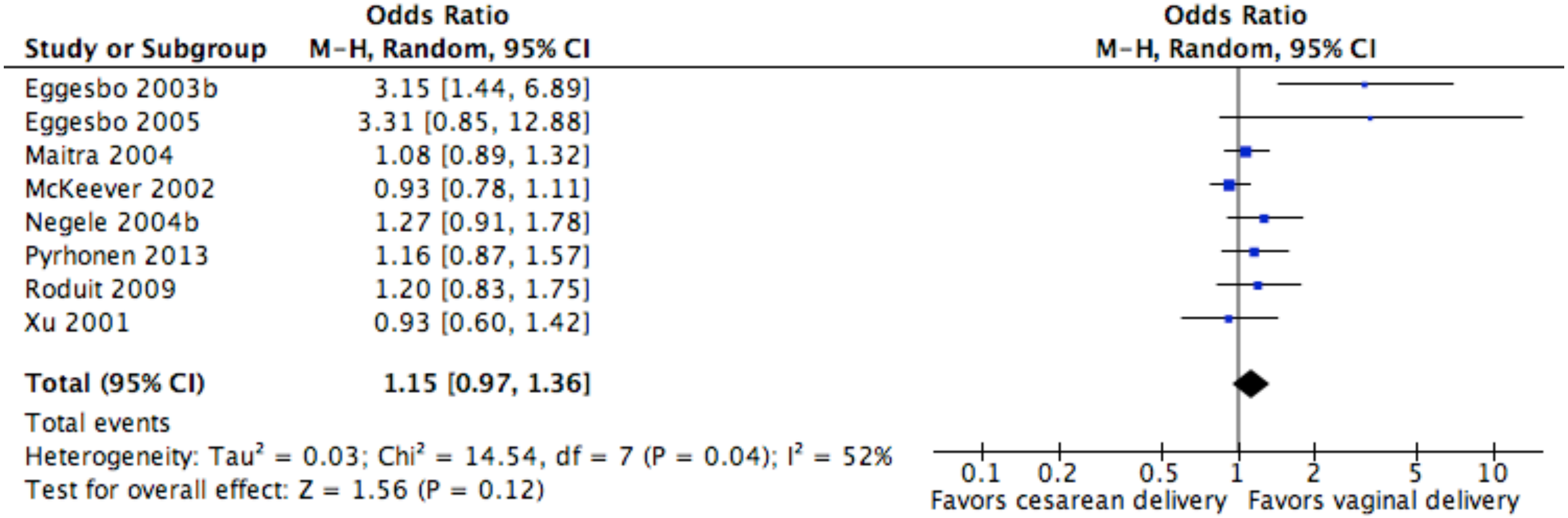

Supplement: S13 Fig — (PDF) [file pmed.1002494.s022.pdf]
