# Supplementary material for: Long-term risks and benefits associated with cesarean delivery for mother, baby, and subsequent pregnancies: Systematic review and meta-analysis
Source: PLoS Med. 2018 Jan 23;15(1):e1002494. doi: 10.1371/journal.pmed.1002494 (PMC5779640; doi:10.1371/journal.pmed.1002494)

S14 Figure: A random effects meta-analysis of children being overweight at 3-8 years old after cesarean delivery compared to vaginal delivery

Study or Subgroup	Odds Ratio M-H, Random, 95% CI	Odds Ratio M-H, Random, 95% CI
Li 2014	1.24 [1.19, 1.29]	
Lin 2013	1.02 [0.84, 1.25]	
Pei 2014	1.34 [0.89, 2.02]	
Steur 2011	1.70 [1.11, 2.61]	
**Total (95% CI)**	**1.22 [1.06, 1.41]**	
Total events		

Heterogeneity: $Tau^2 = 0.01$; $Chi^2 = 5.68$, $df = 3$ ($P = 0.13$); $I^2 = 47\%$

Test for overall effect: $Z = 2.69$ ($P = 0.007$)

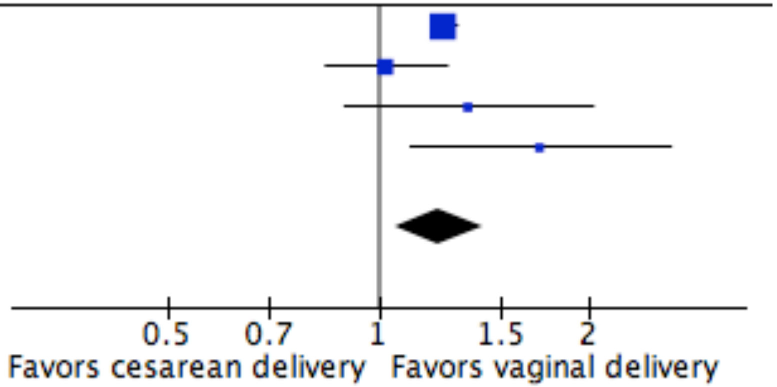

Supplement: S14 Fig — (PDF) [file pmed.1002494.s023.pdf]
