# Supplementary material for: Long-term risks and benefits associated with cesarean delivery for mother, baby, and subsequent pregnancies: Systematic review and meta-analysis
Source: PLoS Med. 2018 Jan 23;15(1):e1002494. doi: 10.1371/journal.pmed.1002494 (PMC5779640; doi:10.1371/journal.pmed.1002494)

S15 Figure: A random effects meta-analysis of obesity in children up to 5 years old after cesarean delivery compared to vaginal delivery

Study or Subgroup	Odds Ratio M–H, Random, 95% CI
Barros 2012a	1.50 [1.20, 1.87]
Barros 2012b	1.85 [1.27, 2.68]
Barros 2012c	1.62 [1.33, 1.96]
Black 2015	1.22 [1.08, 1.38]
Huh 2012	2.29 [1.53, 3.42]
Pei 2014	1.72 [1.18, 2.53]
**Total (95% CI)**	**1.59 [1.33, 1.90]**
Total events	

Heterogeneity: $Tau^2 = 0.03$; $Chi^2 = 15.78$, df = 5 (P = 0.008); $I^2 = 68\%$

Test for overall effect: Z = 5.05 (P < 0.00001)

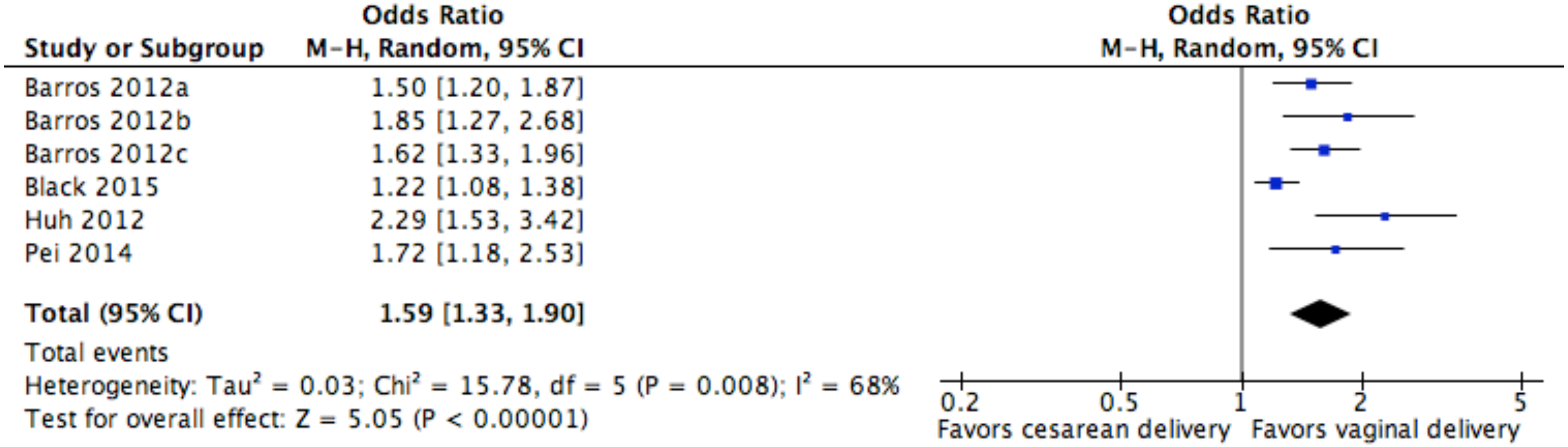

Supplement: S15 Fig — (PDF) [file pmed.1002494.s024.pdf]
