# Supplementary material for: Long-term risks and benefits associated with cesarean delivery for mother, baby, and subsequent pregnancies: Systematic review and meta-analysis
Source: PLoS Med. 2018 Jan 23;15(1):e1002494. doi: 10.1371/journal.pmed.1002494 (PMC5779640; doi:10.1371/journal.pmed.1002494)

S16 Figure: A random effects meta-analysis of obesity in children 6-15 years old after cesarean delivery compared to vaginal delivery

Study or Subgroup	Odds Ratio M-H, Random, 95% CI
Ajslev 2011	1.15 [1.03, 1.29]
Barros 2012b	1.52 [1.24, 1.85]
Goldani 2013a	1.88 [1.21, 2.92]
Goldani 2013b	2.53 [0.90, 7.06]
Pei 2014	1.50 [0.84, 2.68]
**Total (95% CI)**	**1.45 [1.15, 1.83]**
Total events	

Heterogeneity: $Tau^2 = 0.03$; $Chi^2 = 10.85$, df = 4 (P = 0.03); $I^2 = 63\%$

Test for overall effect: Z = 3.17 (P = 0.002)

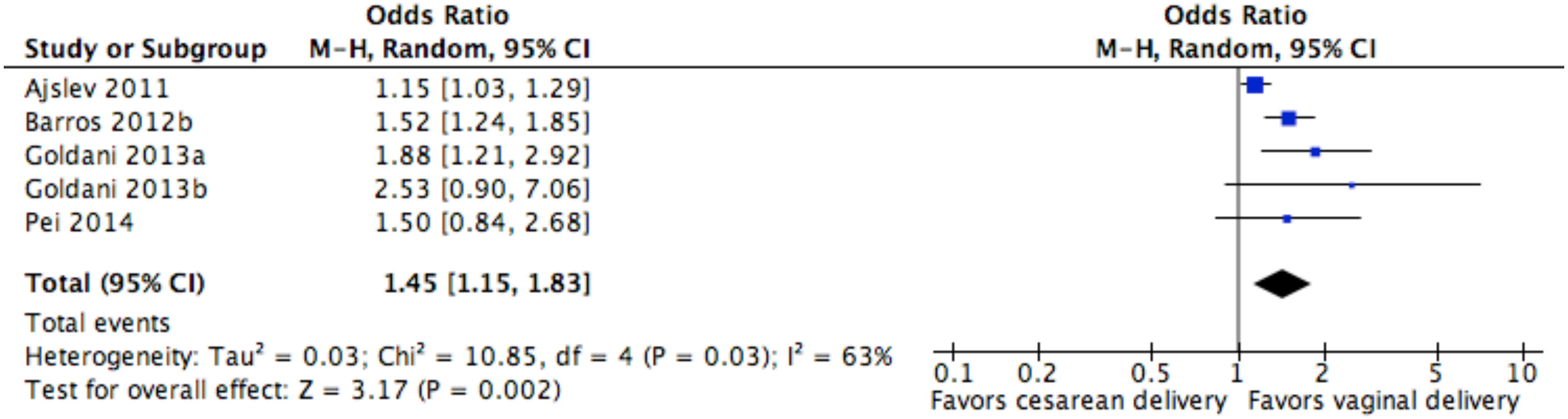

Supplement: S16 Fig — (PDF) [file pmed.1002494.s025.pdf]
