# Supplementary material for: Long-term risks and benefits associated with cesarean delivery for mother, baby, and subsequent pregnancies: Systematic review and meta-analysis
Source: PLoS Med. 2018 Jan 23;15(1):e1002494. doi: 10.1371/journal.pmed.1002494 (PMC5779640; doi:10.1371/journal.pmed.1002494)

S17 Figure: A fixed effects meta-analysis of adulthood obesity after cesarean delivery compared to vaginal delivery

Study or Subgroup	Odds Ratio M–H, Fixed, 95% CI
Barros 2012a	1.11 [0.88, 1.41]
Changzheng 2016	1.36 [1.24, 1.49]
Goldani 2011	1.54 [1.17, 2.03]
Mamun 2013	1.23 [0.88, 1.72]
Mesquita 2013	1.36 [1.12, 1.66]
**Total (95% CI)**	**1.34 [1.25, 1.44]**
Total events	

Heterogeneity: $Chi^2 = 3.76$, df = 4 (P = 0.44); $I^2 = 0\%$

Test for overall effect: Z = 7.90 (P < 0.00001)

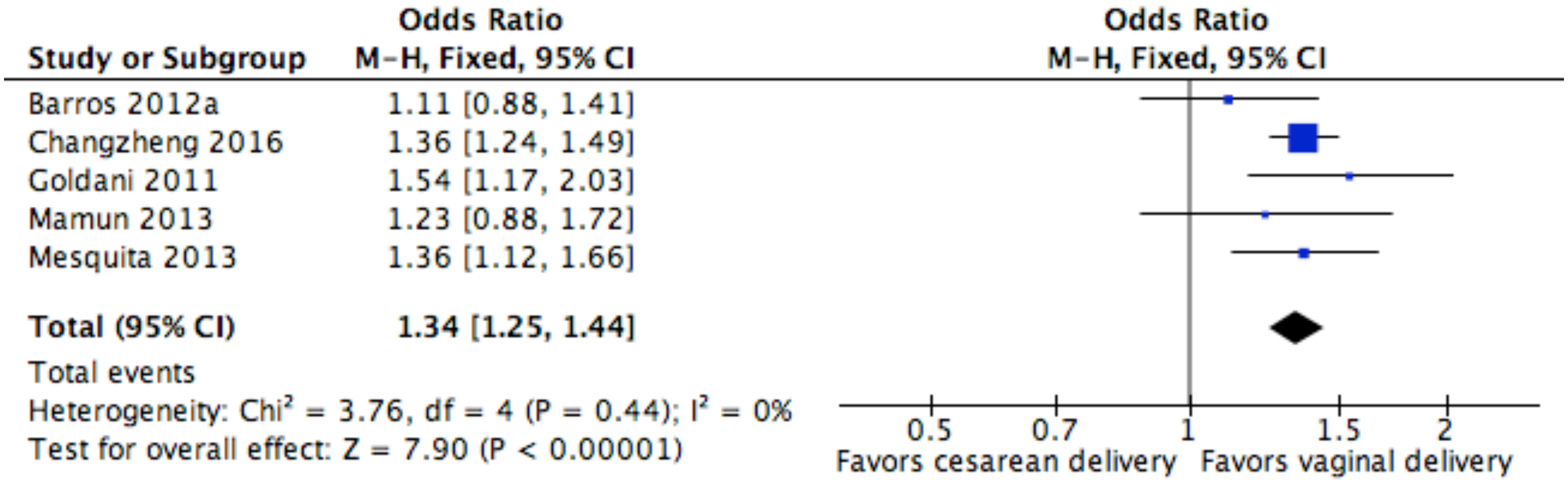

Supplement: S17 Fig — (PDF) [file pmed.1002494.s026.pdf]
