# Supplementary material for: Long-term risks and benefits associated with cesarean delivery for mother, baby, and subsequent pregnancies: Systematic review and meta-analysis
Source: PLoS Med. 2018 Jan 23;15(1):e1002494. doi: 10.1371/journal.pmed.1002494 (PMC5779640; doi:10.1371/journal.pmed.1002494)

S18 Figure: A fixed effects meta-analysis of inflammatory bowel disease in children and adults up to age 35 years after cesarean delivery compared to vaginal delivery

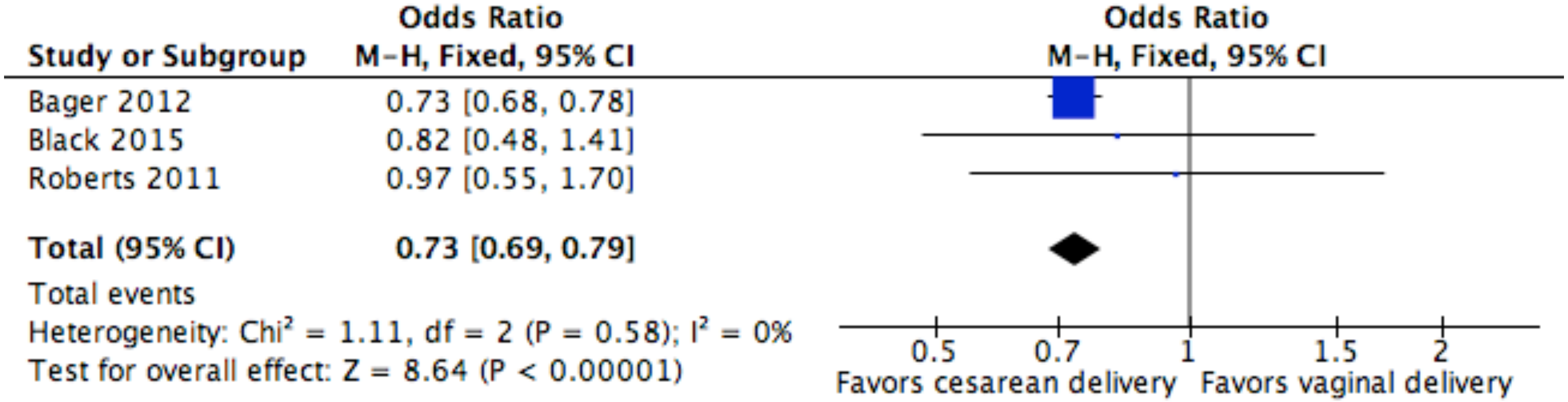

Supplement: S18 Fig — (PDF) [file pmed.1002494.s027.pdf]
