# Supplementary material for: Long-term risks and benefits associated with cesarean delivery for mother, baby, and subsequent pregnancies: Systematic review and meta-analysis
Source: PLoS Med. 2018 Jan 23;15(1):e1002494. doi: 10.1371/journal.pmed.1002494 (PMC5779640; doi:10.1371/journal.pmed.1002494)

S19 Figure: A fixed effects meta-analysis of perinatal death in pregnancy after cesarean delivery compared to pregnancy after vaginal delivery

Study or Subgroup	Odds Ratio M-H, Fixed, 95% CI
Hemminki 2005	0.98 [0.71, 1.34]
Kennare 2007	1.29 [0.94, 1.79]
**Total (95% CI)**	**1.11 [0.89, 1.39]**
Total events	

Heterogeneity: $Chi^2 = 1.51$, df = 1 (P = 0.22); $I^2 = 34\%$
Test for overall effect: Z = 0.93 (P = 0.35)

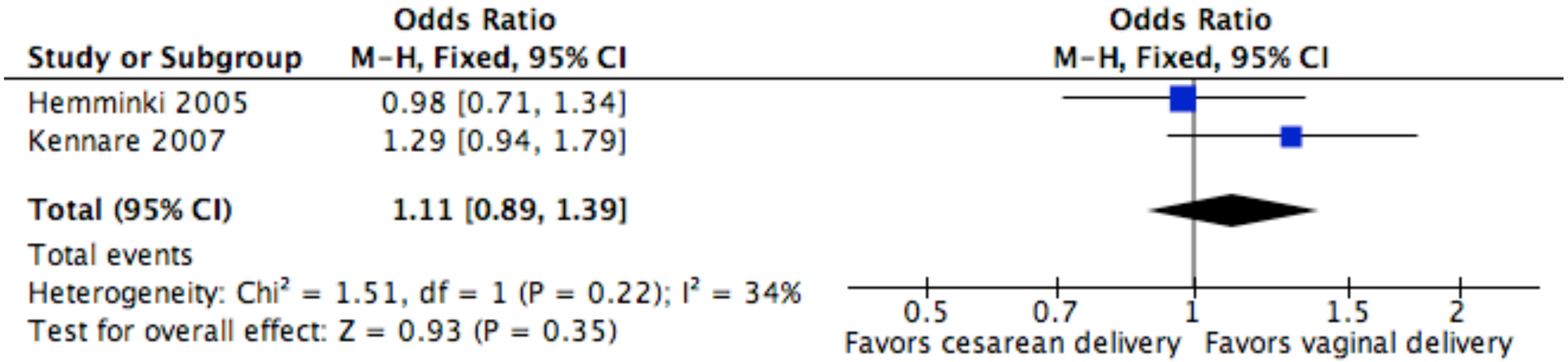

Supplement: S19 Fig — (PDF) [file pmed.1002494.s028.pdf]
