# Supplementary material for: Long-term risks and benefits associated with cesarean delivery for mother, baby, and subsequent pregnancies: Systematic review and meta-analysis
Source: PLoS Med. 2018 Jan 23;15(1):e1002494. doi: 10.1371/journal.pmed.1002494 (PMC5779640; doi:10.1371/journal.pmed.1002494)

S20 Figure: A random effects meta-analysis of placenta previa in pregnancy after cesarean delivery compared to pregnancy after vaginal delivery

Study or Subgroup	Odds Ratio M–H, Random, 95% CI
Daltveit 2008	1.64 [1.41, 1.91]
Downes 2015	2.04 [1.26, 3.30]
Getahun 2006	1.66 [1.42, 1.94]
Gurol–Urganci 2011	1.99 [1.82, 2.17]
Jackson 2012	1.84 [1.15, 2.95]
Kennare 2007	1.84 [1.45, 2.33]
Lydon–Rochelle 2001	1.47 [1.21, 1.79]
Rasmussen 2000	1.61 [1.28, 2.03]
Salihu 2006	2.00 [1.66, 2.41]
Yang 2007	1.66 [1.59, 1.72]
**Total (95% CI)**	**1.74 [1.62, 1.87]**
Total events	

Heterogeneity: $Tau^2 = 0.01$; $Chi^2 = 19.89$, $df = 9$ ($P = 0.02$); $I^2 = 55\%$

Test for overall effect: $Z = 14.97$ ($P < 0.00001$)

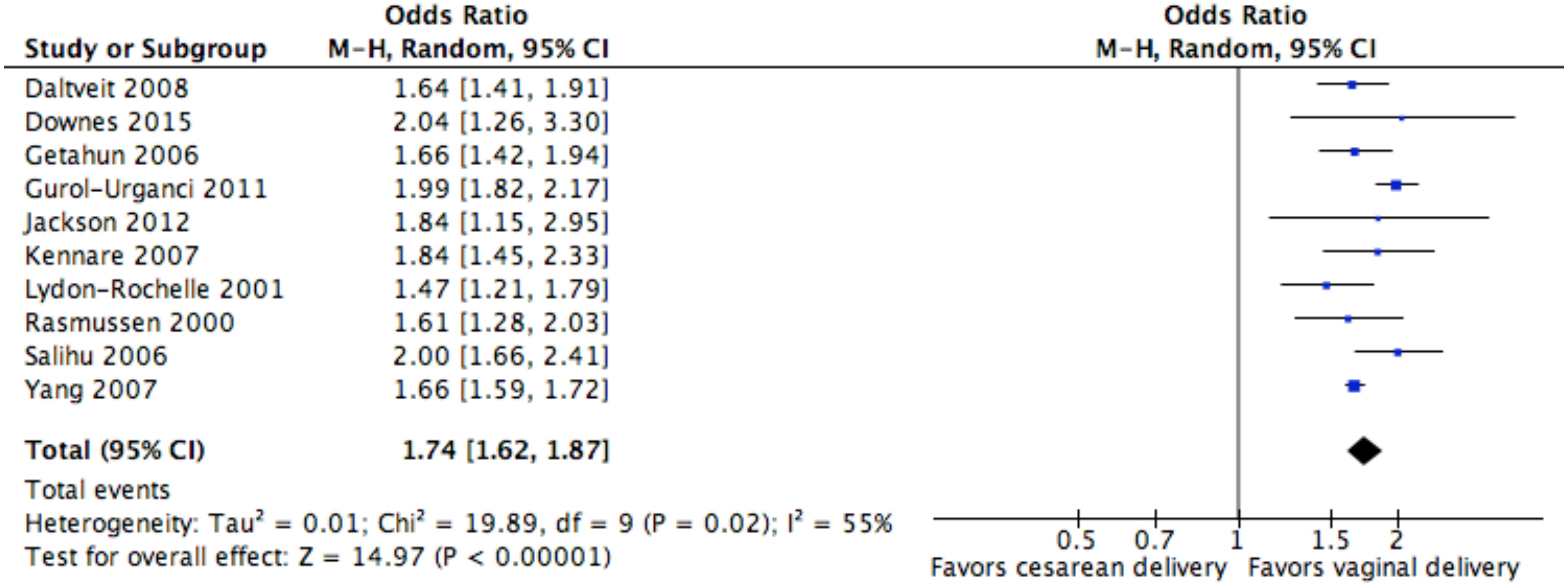

Supplement: S20 Fig — (PDF) [file pmed.1002494.s029.pdf]
