# Supplementary material for: Long-term risks and benefits associated with cesarean delivery for mother, baby, and subsequent pregnancies: Systematic review and meta-analysis
Source: PLoS Med. 2018 Jan 23;15(1):e1002494. doi: 10.1371/journal.pmed.1002494 (PMC5779640; doi:10.1371/journal.pmed.1002494)

S21 Figure: A random effects meta-analysis of placenta accreta in pregnancy after cesarean delivery compared to pregnancy after vaginal delivery

Study or Subgroup	Odds Ratio M-H, Random, 95% CI
Daltveit 2008	2.31 [1.62, 3.29]
Jackson 2012	2.28 [0.73, 7.07]
Kennare 2007	18.79 [2.26, 156.14]
**Total (95% CI)**	**2.95 [1.32, 6.60]**
Total events	

Heterogeneity: $Tau^2 = 0.25$; $Chi^2 = 3.76$, df = 2 (P = 0.15); $I^2 = 47\%$

Test for overall effect: Z = 2.64 (P = 0.008)

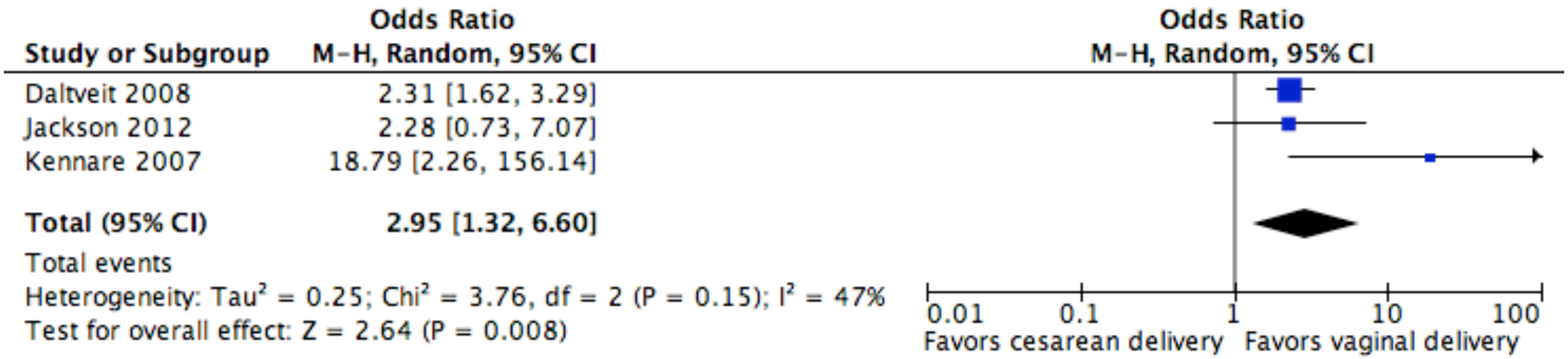

Supplement: S21 Fig — (PDF) [file pmed.1002494.s030.pdf]
