# Supplementary material for: Long-term risks and benefits associated with cesarean delivery for mother, baby, and subsequent pregnancies: Systematic review and meta-analysis
Source: PLoS Med. 2018 Jan 23;15(1):e1002494. doi: 10.1371/journal.pmed.1002494 (PMC5779640; doi:10.1371/journal.pmed.1002494)

S22 Figure: A random effects meta-analysis of placental abruption in pregnancy after cesarean delivery compared to pregnancy after vaginal delivery

Study or Subgroup	Odds Ratio M-H, Random, 95% CI
Getahun 2006	1.29 [1.14, 1.45]
Jackson 2012	2.16 [1.46, 3.19]
Kennare 2007	1.22 [0.92, 1.63]
Lydon-Rochelle 2001	1.26 [1.10, 1.44]
Salihu 2006	1.50 [1.30, 1.74]
Yang 2007	1.41 [1.36, 1.45]
**Total (95% CI)**	**1.38 [1.27, 1.49]**
Total events	

Heterogeneity: $Tau^2 = 0.00$; $Chi^2 = 10.76$, df = 5 (P = 0.06); $I^2 = 54\%$
Test for overall effect: Z = 7.90 (P < 0.00001)

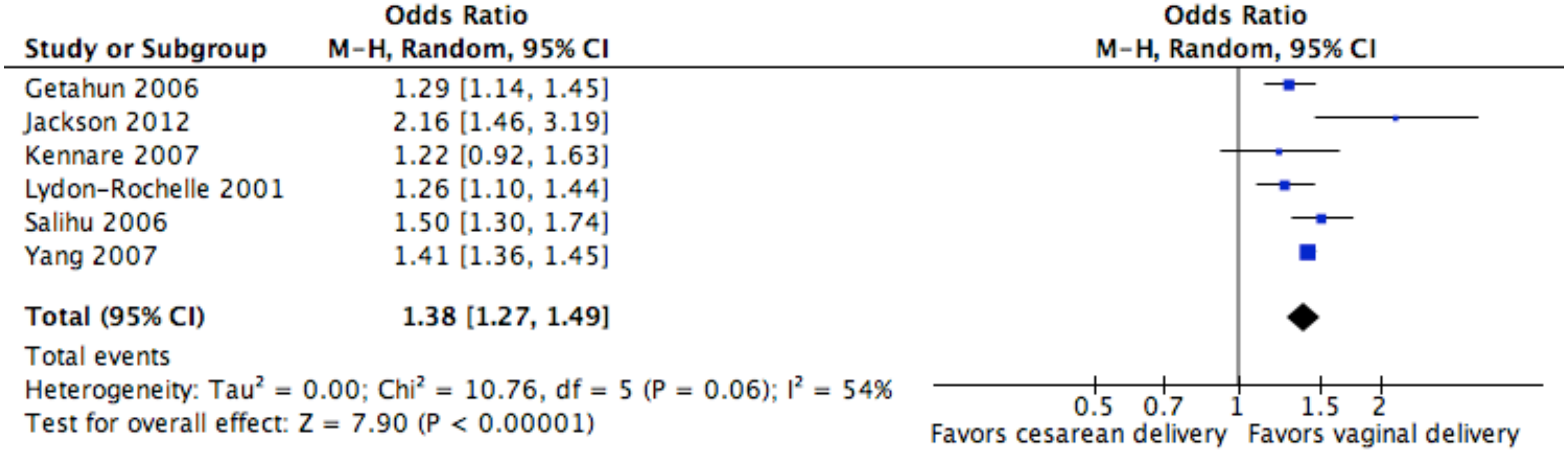

Supplement: S22 Fig — (PDF) [file pmed.1002494.s031.pdf]
