# Supplementary material for: Long-term risks and benefits associated with cesarean delivery for mother, baby, and subsequent pregnancies: Systematic review and meta-analysis
Source: PLoS Med. 2018 Jan 23;15(1):e1002494. doi: 10.1371/journal.pmed.1002494 (PMC5779640; doi:10.1371/journal.pmed.1002494)

S23 Figure: A random effects meta-analysis of uterine rupture in pregnancy after cesarean delivery compared to pregnancy after vaginal delivery

Study or Subgroup	Odds Ratio M-H, Random, 95% CI
Daltveit 2008	33.45 [22.83, 48.99]
Jackson 2012	43.51 [23.63, 80.09]
Kennare 2007	122.35 [7.39, 2026.56]
Taylor 2005	6.72 [2.91, 15.52]
**Total (95% CI)**	**25.81 [10.96, 60.76]**
Total events	

Heterogeneity: $Tau^2 = 0.52$; $Chi^2 = 14.99$, df = 3 (P = 0.002); $I^2 = 80\%$

Test for overall effect: Z = 7.44 (P < 0.00001)

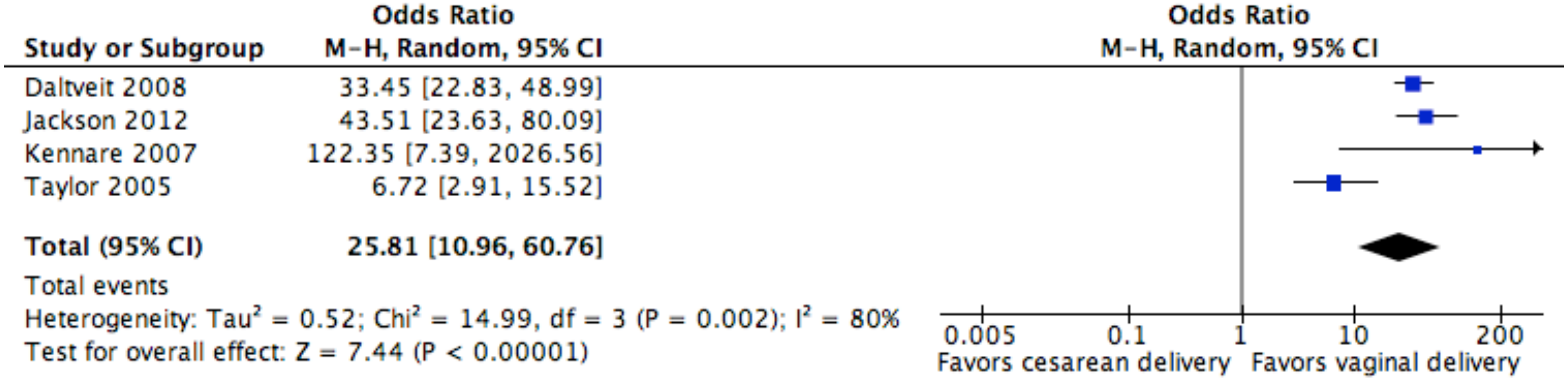

Supplement: S23 Fig — (PDF) [file pmed.1002494.s032.pdf]
