# Supplementary material for: Long-term risks and benefits associated with cesarean delivery for mother, baby, and subsequent pregnancies: Systematic review and meta-analysis
Source: PLoS Med. 2018 Jan 23;15(1):e1002494. doi: 10.1371/journal.pmed.1002494 (PMC5779640; doi:10.1371/journal.pmed.1002494)

S24 Figure: A random effects meta-analysis of miscarriage in pregnancy after cesarean delivery compared to pregnancy after vaginal delivery

Study or Subgroup	Odds Ratio M–H, Random, 95% CI
Hall 1989	1.32 [1.06, 1.65]
Jackson 2012	1.30 [1.19, 1.42]
Mollison 2005	1.06 [0.92, 1.23]
Smith 2006	1.07 [1.00, 1.15]
**Total (95% CI)**	**1.17 [1.03, 1.32]**
Total events	

Heterogeneity: $Tau^2 = 0.01$; $Chi^2 = 13.96$, df = 3 (P = 0.003); $I^2 = 79\%$

Test for overall effect: Z = 2.50 (P = 0.01)

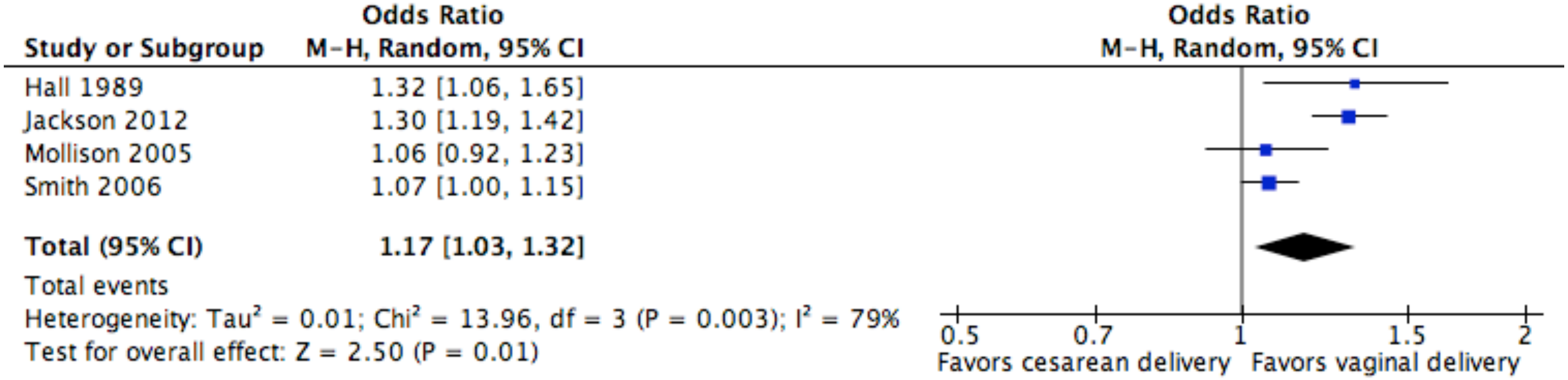

Supplement: S24 Fig — (PDF) [file pmed.1002494.s033.pdf]
