# Supplementary material for: Long-term risks and benefits associated with cesarean delivery for mother, baby, and subsequent pregnancies: Systematic review and meta-analysis
Source: PLoS Med. 2018 Jan 23;15(1):e1002494. doi: 10.1371/journal.pmed.1002494 (PMC5779640; doi:10.1371/journal.pmed.1002494)

S25 Figure: A fixed effects meta-analysis of ectopic pregnancy after cesarean delivery compared to pregnancy after vaginal delivery

Study or Subgroup	Odds Ratio M–H, Fixed, 95% CI
Bowman 2015	1.17 [0.97, 1.41]
Jackson 2012	1.22 [0.91, 1.65]
Mollison 2005	1.41 [0.89, 2.22]
**Total (95% CI)**	**1.21 [1.04, 1.40]**
Total events	

Heterogeneity: $Chi^2 = 0.55$, df = 2 (P = 0.76); $I^2 = 0\%$

Test for overall effect: Z = 2.43 (P = 0.02)

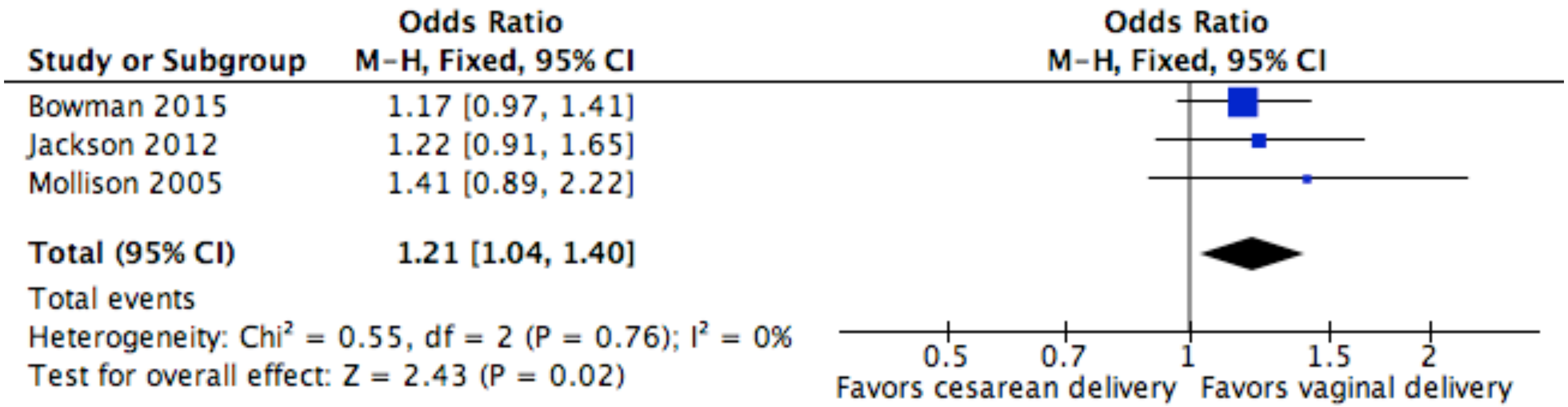

Supplement: S25 Fig — (PDF) [file pmed.1002494.s034.pdf]
