# Supplementary material for: Long-term risks and benefits associated with cesarean delivery for mother, baby, and subsequent pregnancies: Systematic review and meta-analysis
Source: PLoS Med. 2018 Jan 23;15(1):e1002494. doi: 10.1371/journal.pmed.1002494 (PMC5779640; doi:10.1371/journal.pmed.1002494)

S26 Figure: A fixed effects meta-analysis of stillbirth in pregnancy after cesarean delivery compared to pregnancy after vaginal delivery

Study or Subgroup	Odds Ratio M-H, Fixed, 95% CI
Gray 2007	1.34 [0.90, 1.99]
Jackson 2012	0.67 [0.24, 1.86]
Kennare 2007	1.43 [1.00, 2.06]
Moraitis 2015	1.36 [1.07, 1.73]
Osborne 2012	2.62 [1.10, 6.23]
Smith 2003	1.62 [1.24, 2.12]
Taylor 2005	1.11 [0.91, 1.36]
Wood 2008	1.17 [0.97, 1.41]
**Total (95% CI)**	**1.27 [1.15, 1.40]**
Total events	

Heterogeneity: $Chi^2 = 10.55$, $df = 7$ ($P = 0.16$); $I^2 = 34\%$

Test for overall effect: $Z = 4.66$ ($P < 0.00001$)

Odds Ratio
M-H, Fixed, 95% CI

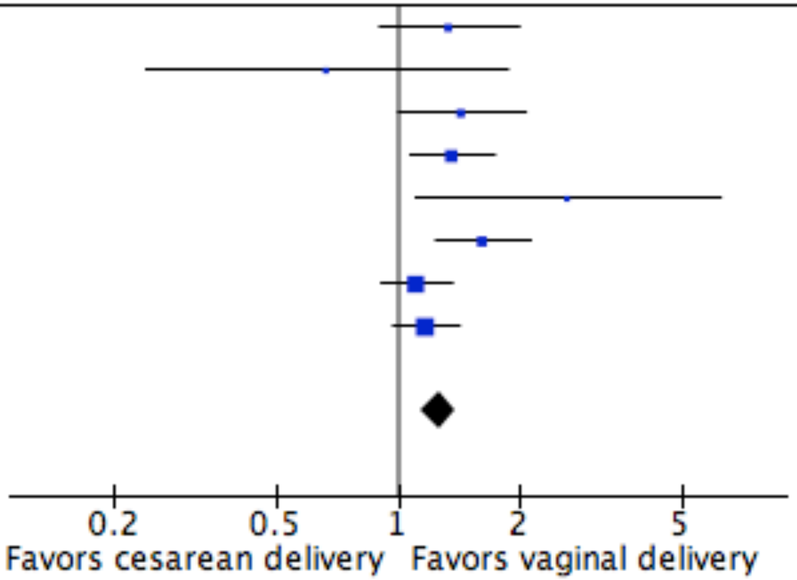

Supplement: S26 Fig — (PDF) [file pmed.1002494.s035.pdf]
