# Supplementary material for: Long-term risks and benefits associated with cesarean delivery for mother, baby, and subsequent pregnancies: Systematic review and meta-analysis
Source: PLoS Med. 2018 Jan 23;15(1):e1002494. doi: 10.1371/journal.pmed.1002494 (PMC5779640; doi:10.1371/journal.pmed.1002494)

S27 Figure: A random effects meta-analysis of hysterectomy in pregnancy after caesarean delivery compared to pregnancy after vaginal delivery

Study or Subgroup	Odds Ratio M-H, Random, 95% CI
Jackson 2012	8.55 [2.30, 31.86]
Taylor 2005	2.24 [1.17, 4.27]
**Total (95% CI)**	**3.85 [1.06, 14.02]**
Total events	

Heterogeneity: $Tau^2 = 0.62$; $Chi^2 = 3.22$, df = 1 (P = 0.07); $I^2 = 69\%$
Test for overall effect: Z = 2.05 (P = 0.04)

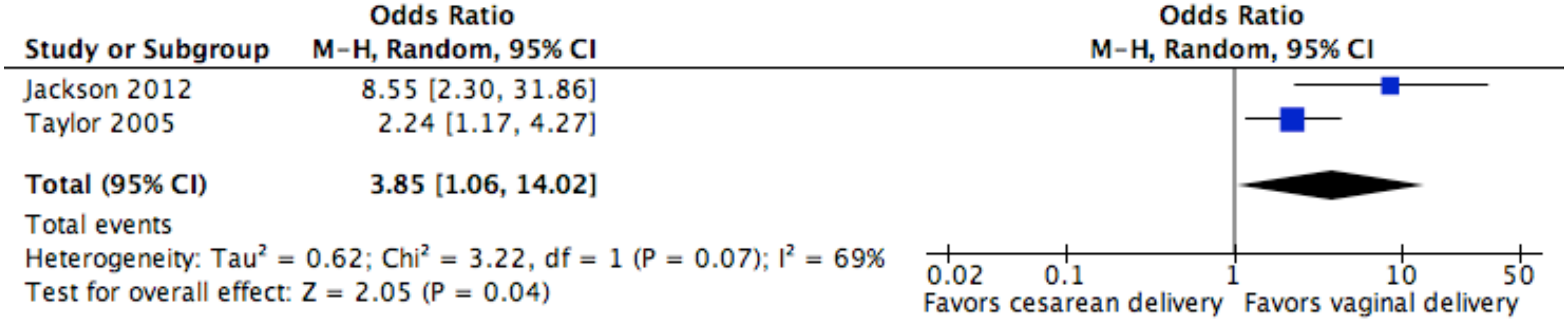

Supplement: S27 Fig — (PDF) [file pmed.1002494.s036.pdf]
