# Supplementary material for: Long-term risks and benefits associated with cesarean delivery for mother, baby, and subsequent pregnancies: Systematic review and meta-analysis
Source: PLoS Med. 2018 Jan 23;15(1):e1002494. doi: 10.1371/journal.pmed.1002494 (PMC5779640; doi:10.1371/journal.pmed.1002494)

S28 Figure: A fixed effects meta-analysis of having antepartum hemorrhage in pregnancy after cesarean delivery compared to pregnancy after vaginal delivery

Study or Subgroup	Odds Ratio M–H, Fixed, 95% CI
Hemminki 2005	1.24 [0.98, 1.56]
Kennare 2007	1.21 [1.06, 1.38]
**Total (95% CI)**	**1.22 [1.09, 1.36]**
Total events	
Heterogeneity: $Chi^2 = 0.03$, df = 1 (P = 0.87); $I^2 = 0\%$	
Test for overall effect: Z = 3.39 (P = 0.0007)	

Odds Ratio
M–H, Fixed, 95% CI

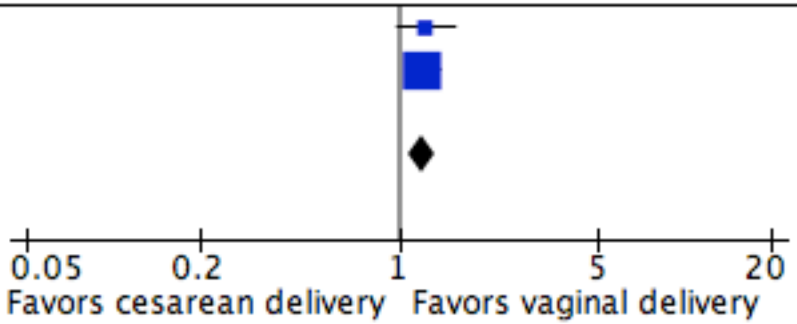

Supplement: S28 Fig — (PDF) [file pmed.1002494.s037.pdf]
