# Supplementary material for: Long-term risks and benefits associated with cesarean delivery for mother, baby, and subsequent pregnancies: Systematic review and meta-analysis
Source: PLoS Med. 2018 Jan 23;15(1):e1002494. doi: 10.1371/journal.pmed.1002494 (PMC5779640; doi:10.1371/journal.pmed.1002494)

S29 Figure: A random effects meta-analysis of postpartum hemorrhage in pregnancy after cesarean delivery compared to pregnancy after vaginal delivery

Study or Subgroup	Odds Ratio M-H, Random, 95% CI
Jackson 2012	0.84 [0.70, 1.00]
Taylor 2005	0.63 [0.59, 0.68]
**Total (95% CI)**	**0.72 [0.55, 0.95]**
Total events	

Heterogeneity: $Tau^2 = 0.04$; $Chi^2 = 8.33$, df = 1 (P = 0.004); $I^2 = 88\%$
Test for overall effect: Z = 2.33 (P = 0.02)

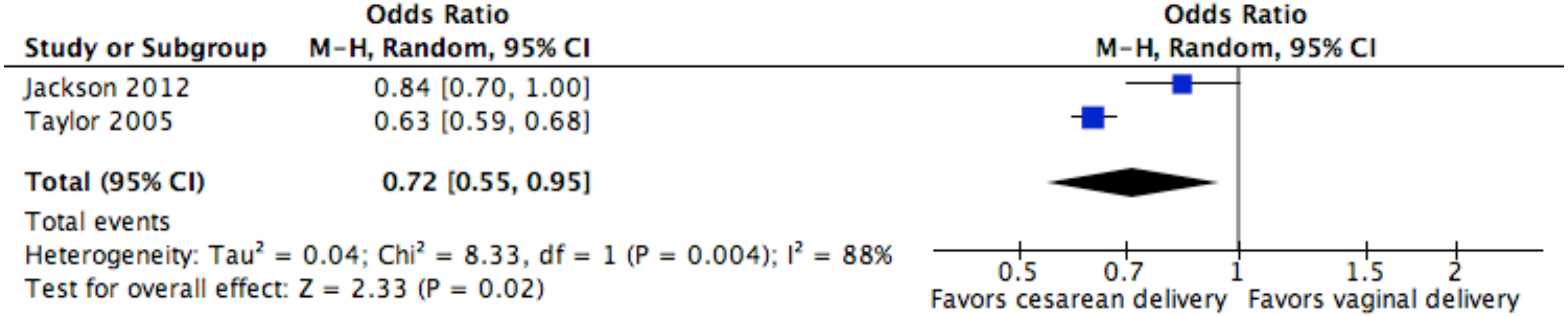

Supplement: S29 Fig — (PDF) [file pmed.1002494.s038.pdf]
