# Supplementary material for: Long-term risks and benefits associated with cesarean delivery for mother, baby, and subsequent pregnancies: Systematic review and meta-analysis
Source: PLoS Med. 2018 Jan 23;15(1):e1002494. doi: 10.1371/journal.pmed.1002494 (PMC5779640; doi:10.1371/journal.pmed.1002494)

S30 Figure: A random effects meta-analysis of preterm labor in pregnancy after cesarean delivery compared to pregnancy after vaginal delivery

Study or Subgroup	Odds Ratio M-H, Random, 95% CI
Hemminki 2005	1.33 [1.19, 1.48]
Huang 2011	1.00 [1.00, 1.01]
Jackson 2012	0.41 [0.23, 0.72]
Kennare 2007	1.22 [1.09, 1.35]
Salihu 2011	0.89 [0.88, 0.91]
Taylor 2005	1.18 [1.10, 1.26]
Wood 2008	1.09 [1.04, 1.15]
**Total (95% CI)**	**1.07 [0.99, 1.16]**
Total events	

Heterogeneity: $Tau^2 = 0.01$; $Chi^2 = 183.97$, df = 6 ($P < 0.00001$); $I^2 = 97\%$

Test for overall effect: Z = 1.79 (P = 0.07)

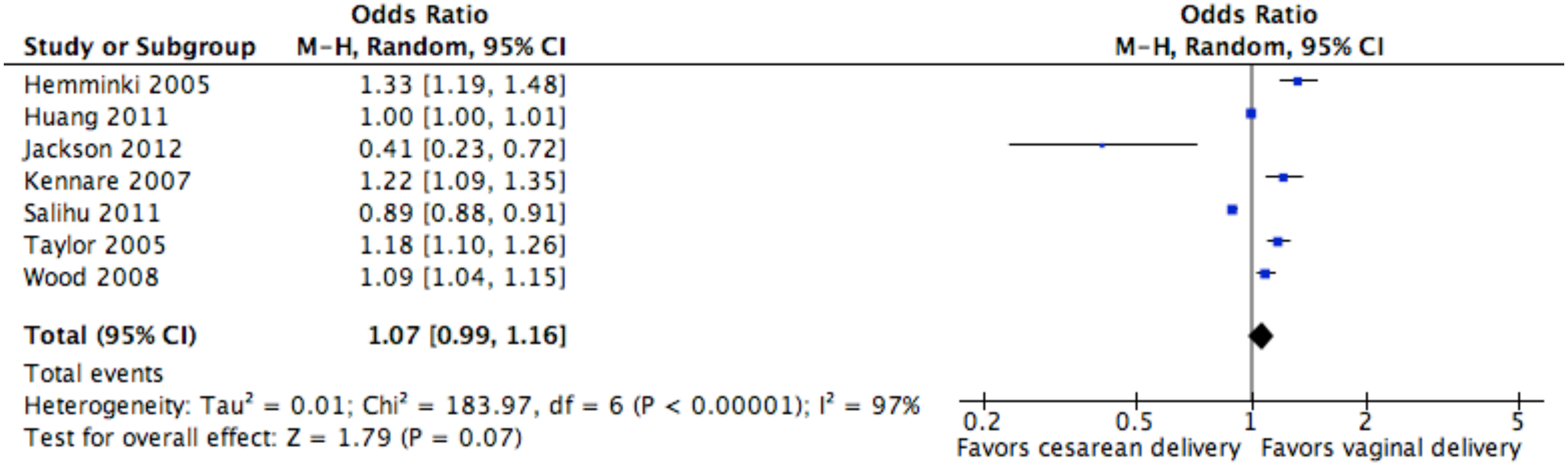

Supplement: S30 Fig — (PDF) [file pmed.1002494.s039.pdf]
