# Supplementary material for: Long-term risks and benefits associated with cesarean delivery for mother, baby, and subsequent pregnancies: Systematic review and meta-analysis
Source: PLoS Med. 2018 Jan 23;15(1):e1002494. doi: 10.1371/journal.pmed.1002494 (PMC5779640; doi:10.1371/journal.pmed.1002494)

S31 Figure: A random effects meta-analysis of having a small for gestational age baby in pregnancy after cesarean delivery compared to pregnancy after vaginal delivery

Study or Subgroup	Odds Ratio M-H, Random, 95% CI
Daltveit 2008	1.27 [1.24, 1.31]
Huang 2011	0.92 [0.91, 0.92]
Kennare 2007	1.04 [0.95, 1.14]
Salihu 2011	0.87 [0.85, 0.89]
Taylor 2005	0.97 [0.92, 1.02]
**Total (95% CI)**	**1.01 [0.89, 1.14]**
Total events	

Heterogeneity: $Tau^2 = 0.02$; $Chi^2 = 460.11$, df = 4 ($P < 0.00001$); $I^2 = 99\%$

Test for overall effect: Z = 0.08 (P = 0.93)

Odds Ratio
M-H, Random, 95% CI

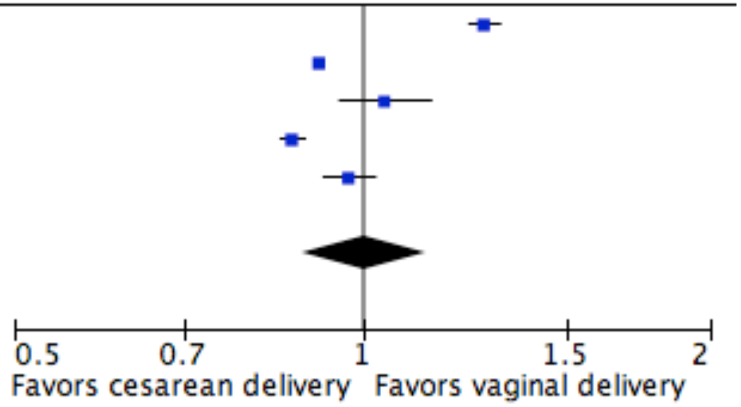

Supplement: S31 Fig — (PDF) [file pmed.1002494.s040.pdf]
