# Supplementary material for: Long-term risks and benefits associated with cesarean delivery for mother, baby, and subsequent pregnancies: Systematic review and meta-analysis
Source: PLoS Med. 2018 Jan 23;15(1):e1002494. doi: 10.1371/journal.pmed.1002494 (PMC5779640; doi:10.1371/journal.pmed.1002494)

S32 Figure: A random effects meta-analysis of having a baby with low birthweight (<2500g) in pregnancy after cesarean delivery compared to pregnancy after vaginal delivery

Study or Subgroup	Odds Ratio M-H, Random, 95% CI
Hemminki 2005	1.54 [1.35, 1.76]
Kennare 2007	1.26 [1.12, 1.42]
Salihu 2011	0.87 [0.85, 0.90]
Wood 2008	1.06 [1.00, 1.13]
**Total (95% CI)**	**1.15 [0.93, 1.43]**
Total events	

Heterogeneity: $Tau^2 = 0.05$; $Chi^2 = 115.55$, df = 3 ($P < 0.00001$); $I^2 = 97\%$

Test for overall effect: $Z = 1.27$ ($P = 0.20$)

Odds Ratio

M-H, Random, 95% CI

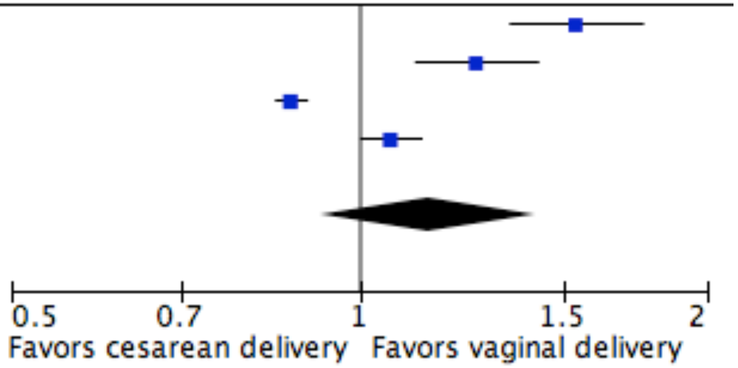

Supplement: S32 Fig — (PDF) [file pmed.1002494.s041.pdf]
