# Supplementary material for: Long-term risks and benefits associated with cesarean delivery for mother, baby, and subsequent pregnancies: Systematic review and meta-analysis
Source: PLoS Med. 2018 Jan 23;15(1):e1002494. doi: 10.1371/journal.pmed.1002494 (PMC5779640; doi:10.1371/journal.pmed.1002494)

S33 Figure: A fixed effects meta-analysis of neonatal death following a pregnancy after cesarean delivery compared to pregnancy after vaginal delivery

Study or Subgroup	Odds Ratio M-H, Fixed, 95% CI
Galyean 2009	1.49 [0.51, 4.36]
Huang 2011	1.01 [0.98, 1.05]
Kennare 2007	0.88 [0.42, 1.84]
Salihu 2011	1.01 [0.91, 1.13]
Taylor 2005	1.33 [0.94, 1.89]
**Total (95% CI)**	**1.01 [0.98, 1.05]**
Total events	

Heterogeneity: $Chi^2 = 2.95$, $df = 4$ ($P = 0.57$); $I^2 = 0\%$

Test for overall effect: $Z = 0.85$ ($P = 0.40$)

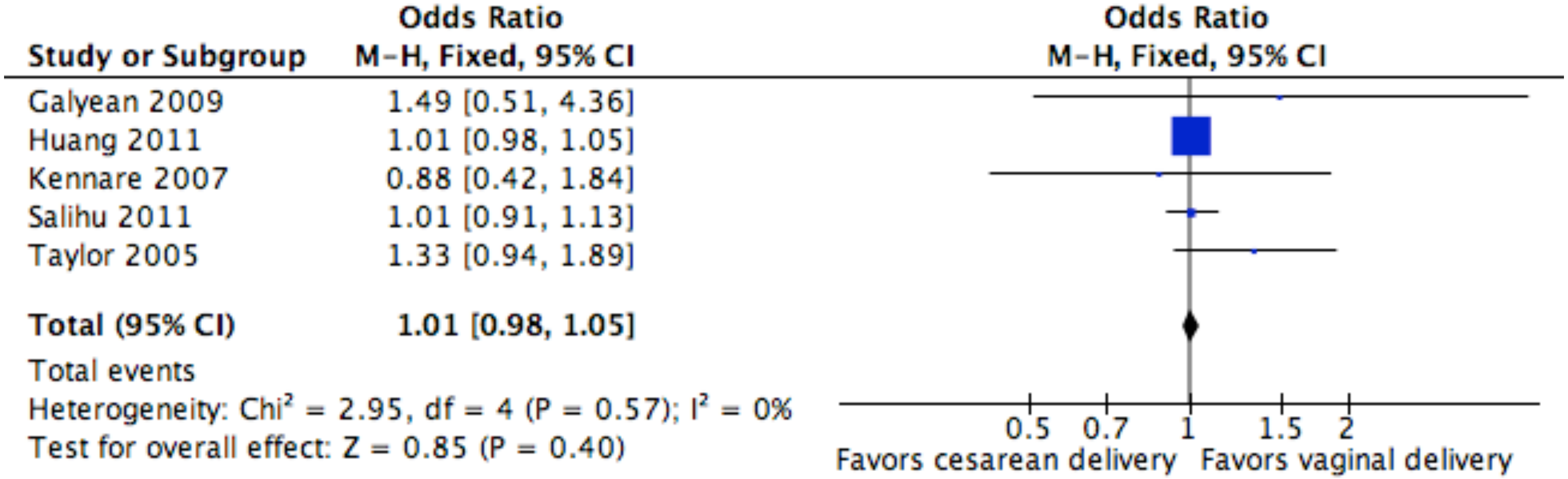

Supplement: S33 Fig — (PDF) [file pmed.1002494.s042.pdf]
